# Interactions between folate metabolism-related nutrients and polymorphisms on colorectal cancer risk: a case-control study in the Basque country

**DOI:** 10.1007/s00394-024-03371-5

**Published:** 2024-04-23

**Authors:** Sara Corchero-Palacios, Iker Alegria-Lertxundi, Marian M. de Pancorbo, Marta Arroyo-Izaga

**Affiliations:** 1 Department of Pharmacy and Food Sciences, Faculty of Pharmacy, University of the Basque Country UPV/EHU, Vitoria-Gasteiz (Araba/Álava), 01006 Spain; 2 Department of Z. and Cellular Biology A., Faculty of Pharmacy, University of the Basque Country UPV/EHU, Vitoria-Gasteiz (Araba/Álava), 01006 Spain; 3 BIOMICs Research Group, Microfluidics & BIOMICs Cluster, Lascaray Research Center, University of the Basque Country UPV/EHU, Bioaraba, BA04.03, 01006 Vitoria-Gasteiz (Araba/Álava), Spain

**Keywords:** Colorectal cancer, Risk factor, Nutrient, Genetic polymorphism, Gene-nutrient interaction, Case-control study

## Abstract

Folate-mediated one-carbon metabolism (FOCM) plays an important role in colorectal carcinogenesis. Previous studies have assessed the role of folate-mediated one-carbon metabolism (FOCM)-related gene-diet interaction in the aetiology of colorectal cancer (CRC), however, the results remained inconclusive. Thus, this study aimed to investigate dietary factors and genetic variants related to FOCM, as well as potential nutrient-gene and nutrient-lifestyle interactions, on CRC risk. This observational study included 229 patients diagnosed with CRC and 229 age- and sex-matched subjects as controls from a population-based bowel cancer screening program. Conditional logistic regression was used to calculate odds ratios (ORs) and 95% confidence intervals (95%CI) for CRC risk. A Bonferroni-corrected threshold of α = 0.005 was considered significant, and *P* values less than 0.05 were considered to be suggestive of an association. After Bonferroni correction, a high dietary intake of betaine was associated with a decreased risk of CRC in the adjusted model (OR, 95% CI: 0.21, 0.10–0.40, *P* < 0.001). Two SNPs, rs1476413 and rs17824591, exhibited significant gene-diet interactions with total choline ad vitamin B_12_ intakes, respectively, in adjusted models (total choline, tertile 3 vs. 1, OR, 95% CI: 0.25, 0.11–0.66, *P*_interaction_ = 0.012; vitamin B_12_, tertile 2 vs. tertile 1, OR, 95% CI: 2.48, 1.04-5.00, *P*_interaction_ = 0.003). These findings suggest that betaine intake and interactions between some dietary factors and variants in *MTHFR* and *MTHFD1* genes have an influence on CRC risk in the population studied. If these results are confirmed, specific nutritional intervention strategies could be designed.

**Supplementary Information:**

The online version contains supplementary material available at 10.1007/s00394-024-03371-5.

**Novelty and Impact – What´s new?**

Previous studies have assessed the role of folate-mediated one-carbon metabolism (FOCM)-related gene-diet interaction in the aetiology of colorectal cancer (CRC), however, the results remained inconclusive. Here, the authors investigate this type of interaction. The findings highlight the importance of interactions between total choline and vitamin B_12_ intakes, and variants in *MTHFR* and *MTHFD1* genes on CRC risk. If these results are confirmed, they may provide valuable risk stratification guidance for diet recommendations.

## Introduction

Colorectal cancer (CRC) is the third most frequent cancer and the second highest mortality in cancer patients worldwide [1]. In Spain, CRC is currently the most frequently diagnosed tumour, with 41,661 new cases (25,415 in men and 16,246 in women) detected in 2022^2^. This incidence is comparable to that found in high-risk zones of Occidental Europe, North America, Australia, and Japan [3]. 

Although screening for early detection of CRC is effective in decreasing trends in mortality rates, understanding the factors involved in daily life are CRC-diagnosed also important for a proactive approach to prevent this type of cancer [4]. The primary prevention for CRC is mostly associated with diet, lifestyle factors, and metabolic diseases. Regarding dietary factors, one-carbon metabolism (1CM)-related nutrients (such as folate, other B vitamins, methionine (Met), choline, and betaine) have been considered anticarcinogenic and chemotherapeutic agents in the 1 C metabolic network [5], whereas alcohol antagonizes 1CM, and its high consumption has been related to higher CRC risk [6]. In addition, the observed inverse association between folate status and CRC risk was further modified by genetic polymorphisms of the enzymes involved in folate metabolism, most notably methylene tetrahydrofolate reductase (MTHFR) [5]. 

However, not only the influence of polymorphisms but also the influences of 1CM-related nutrients on genetic polymorphisms in relation to interaction CRC risk remain largely unexplored. Most studies on this type of gene-diet interaction have focused on folate, B vitamin, and methionine intake [7]. To date, there are no studies in which the intake of choline and/or betaine has been evaluated. To better elucidate the role of genetic factors and environmental conditions, especially diet, on CRC risk, this study had a triple aim: (i) to investigate dietary factors (dietary methyl donors and dietary components that potentially modulate the bioavailability of methyl groups) and genetic variants in methyl metabolizing enzymes; (ii) to determine the potential nutrient-gene interactions; and (iii) to analyse the potential nutrient-lifestyle interactions, that is, interactions between the consumption of the dietary factors mentioned on the first objective and other lifestyle factors.

These aims refer to the CRC risk, in a sample of cases and controls, matched on age and sex, from the population-based bowel cancer screening program (BCSP) of the Osakidetza/Basque Health Service. In particular, the dietary factors investigated were: intakes of folate, vitamins B_2_, B_6,_ and B_12_, Met, choline, betaine, and ethanol; the genetic variants: DNA methyltransferases (*DNMT3B* and *DNMT1*), *MTHFR*, methylene tetrahydrofolate dehydrogenase 1 (*MTHFD1*), and *Met synthase reductase*; and the other lifestyle factors: physical exercise (PE), smoking, and alcohol consumption.

## Methods

### Study participants

Overall, this epidemiologic study is an observational analytic case-control study designed to address possible gene-diet interactions in relation to CRC. Participants in this study were recruited from among patients attending any of the three hospitals of the Osakidetza/Basque Health Service (Basurto, Galdakao, and Donostia) members of the Basque Country’s BCSP. To be eligible for this BCSP, the patients had to be aged between 50 and 69, asymptomatic for colorectal symptoms, and registered with the Osakidetza/Basque Health Service.

These inclusion criteria were applied to both case and control groups; that is, controls fulfilled the same eligibility criteria defined for the cases, except for the disease (outcome). The age- and sex-matched controls were patients with positive results (abnormal) for an immunochemical faecal occult blood test and negative colonoscopy results (normal). Recruitment and data collection through questionnaires were conducted between 2014 and 2016. The start date of the study was 2014 because the BCSP in the Basque Country reached the whole target population (approximately 586,700 people) at the beginning of this year.

The characteristics of the sampling and the cases (pathological staging, location of cancer, tumour grade, and treatments) have been described before [8]. Briefly, 72% were diagnosed with early-stage (I/II) CRC and 76% had a distal location of cancer. The total sample consisted of 308 cases who were diagnosed with CRC and 308 age- and sex-matched controls. However, in the present study, data from 229 CRC patients and 229 controls were analysed, since this is the sample from which biological samples and associated data were obtained. All participants had data on the main dietary factors (folate, vitamin B_2_, B_6_, B_12_, Met, choline, and betaine) and genetic variants that were included in the present study.

This study was conducted according to the guidelines laid down in the Declaration of Helsinki, and all procedures involving patients were approved by the Clinical Research Ethics Committee of the Basque Country (protocol code PI2011006, data of approval 03/23/2012; and PI2014042, data of approval 05/28/2014). Written informed consent was obtained from all the study participants.

### Dietary assessment

Diets were assessed using a short food frequency questionnaire (FFQ) that was a modified version of the Rodríguez et al. questionnaire [9]. This adaptation was validated with multiple 24-h recalls in the Basque general population [10] and CRC-diagnosed patients in a pilot of the present study [11]. It consisted of 67 items and requires the subjects to recall the number of times each food item was consumed either per week or per month. Moreover, the respondents could also record the consumption of other foods that were not included on the food list, as well as the use of dietetic products and nutritional supplements (generic and brand-name, dose, and frequency).

Once the completed FFQ was received, it was reviewed by a dietitian. Consumption frequencies were standardised to “per day” and multiplied by standard serving sizes (grams) [12]. For items that included several foods, each food’s contribution was estimated with weighting coefficients that were obtained from the usual consumption data [13]. All food items that were consumed were entered into DIAL 2.12 (2011 ALCE INGENIERIA), a type of dietary assessment software, to estimate energy intake (kcal/day), dietary fibre, and 1CM-related vitamins (B_2_, B_6_, folate, and B_12_). The intakes of other 1CM-related compounds, in particular, Met, total choline, and betaine were estimated using the United States Department of Agriculture (USDA) Food Data central database [14] and from the publication of Zeisel et al. [15].

### Assessment of covariates

Potential confounders of CRC risk were selected based on published evidence from European Prospective Investigation into Cancer and Nutrition (EPIC), the International Agency for Research on Cancer (IARC), and the World Cancer Research Fund (WCRF), which included: overweight or obesity based on self-reported body mass index (BMI), age, sex, PE (expressed as daily minutes of cycling/sports), smoking (never vs. past/currently, and intensity of smoking measured by the number of cigarettes smoked per day), and alcohol consumption (reported as grams of ethanol per day). In addition, the use of drugs related to decreasing CRC risk (antiplatelet, including non-steroidal anti-inflammatory drugs, and anticoagulants) [16] was recorded. These questions were taken from the Spanish Health Questionnaire [17]. BMI, estimated from self-reported height and weight, was classified according to the WHO criteria for those under 65 years of age [18] and according to the criteria proposed by Silva Rodrigues et al. for those 65 and older [19]. The characteristics of the sample is shown in the Supplementary Material (Table S1).

On the other hand, the FFQ used to assess dietary intake included specific questions about the frequency of intake of the following five major types of alcoholic beverages: beer, wine, cider, aperitif with alcohol, and liquor. These consumption frequencies were standardised to “per day” and multiplied by standard serving sizes (ml) [20]. The alcohol consumption data were expressed as grams of ethanol/day that were estimated with the software DIAL 2.12 (2011 ALCE INGENIERIA) and standard drink units [21]. We used the standard drink unit defined for Spain (one standard drink unit is the equivalent of 10 g of ethanol). With this information, the participants were categorised into those who did and did not meet the recommendations [20]. Those participants who did not meet the recommendations were categorised as “high-risk consumption.”

The differences in general characteristics (age, BMI, PE, smoking, and alcohol consumption, among others) between cases and controls were previously described [22]. Briefly, significant differences between cases and controls were found for smoking and weight status, with a higher percentage of cases with past or current smoking status and with overweight/obesity compared to controls (*P* < 0.01). However, no significant differences were found in alcohol consumption between cases and controls (*P* > 0.05).

Additionally, in both cases and controls, socioeconomic level, and health status (specifically health resource consumption) data were assessed with two indices that were obtained from the clinical databases developed by the Health Department of the Basque Government, namely the socioeconomic deprivation index (DI) and predictive risk modelling (PRM), respectively. The first one was estimated using the MEDEA project criteria, as has been described elsewhere, [23] and was divided into quintiles, with the first being the least disadvantaged and the fifth being the most disadvantaged. The PRM is an index that is based on Adjusted Clinical Groups [24] and Clinical Risk Groups [25]. This index combines information about diagnoses, prescriptions, previous costs, and the use of specific procedures. It can predict the use of health resources, and it was stratified into four levels: the first included participants with a risk of high health resource consumption, and the fourth included those with low health resource consumption. The differences in these two indices (DI and PRM) between cases and controls were previously described [22].

### Biological samples and genotyping

In this study, healthy tissues or saliva samples of cases and controls were collected and genotyped. Samples were provided by the Basque Biobank for Research-OEHUN (www.biobancovasco.org) and were processed following standard operating procedures with appropriate approval of the Ethical and Scientific Committees. DNA was extracted using AllPrep DNA / RNA kit (Qiagen) for paraffin-embedded tissue samples and AutoGenFlex Tissue DNA Extraction kit (Autogen) for mouthwash saliva samples and then was quantified with NanoDrop™ Spectrophotometer (ThermoFisher). Double-stranded DNA was quantified by fluorometry using the Quant-iT™ PicoGreen1 dsDNA Assay Kit (Invitrogen, CA) on a DTX 880 Multimode Detector (Beckman Coulter) to normalize DNA concentration.

After an updated summary of the published genetic variants in methyl metabolizing enzymes related to CRC risk, a total of ten single nucleotide polymorphisms (SNPs) were identified, in particular: *DNMT3B* (rs2424913, rs406193), [26] *DNMT1* (rs2228612), [27] *MTHFR* (rs1476413, rs1801131, rs1801133), [28, 29] *MTHFD1* (rs8003379, rs17824591) and *Met synthase reductase* (rs1801394, rs10380) [29]. These SNPs were organised in the context of the gene(s) at or near the locus and chromosome locus. The allelic discrimination was assessed using the MassARRAY1 System (Agena Bioscience) on CeGen-PRB2-ISCII (Nodo USC) following the procedure provided by the manufacturer. Quality control samples were included in the genotyping assays.

### Statistical analysis

The sample size was estimated to be 286 in each group (cases and controls) to detect an odds ratio (OR) of 2.0 with 80% power at a two-sided level of significance of 5%, under an exposure prevalence of 10%, using the Epidat 3.0 program [30].

Data were analysed using IBM SPSS Statistics for Windows, version 22.0 (IBM Corp., Armonk, NY, USA) and STATA 16.0 (StataCorp LP, Texas, USA). Categorical variables are shown as a percentage, and continuous variables are as the means and standard deviation. Normality was checked using the Kolmogorov–Smirnov–Lilliefors test and since all continuous variables followed a non-normal distribution, the Wilcoxon rank-sum test was used for two related means comparison. The Chi-square test was used to evaluate differences between categorical variables. When expected frequencies were lesser than five, Fisher´s exact test was used. Tests for associations and deviation from Hardy-Weinberg equilibrium were performed separately in cases and controls.

Conditional logistic regression was used to calculate ORs and 95% confidence intervals (95%CI) for CRC risk according to (i) tertiles (T) of dietary compound intake, (ii) dominant and recessive models of SNPs, (iii) nutrient-gene interactions, and (iii) nutrient-lifestyle interactions. The logistic regression models were applied to the total sample and the subgroup of cases with distal location (*n* = 178) and their respective controls (Tables S2, S7, and S8). When the sample size was small (≤ 10 per group), the conditional exact logistic regression model was applied. The logistic regression model could not be applied to the subgroup of cases with proximal location due to the (too small) sample size. The intake of dietary compounds was categorised into Ts based on the distribution in the control group, taking into account sex differences when they were significant. Specifically, different cutoff points were applied to estimate Ts in men and women when significant sex differences were identified, that is, in the case of folate and Met intake. The lowest T was used as the reference group. The most frequent genotype (homozygous) was considered the reference group to calculate ORs in a dominant model, and the most frequent genotype (homozygous) and the heterozygous genotype containing the risk allele were considered the reference group in the recessive model. The nutrient-gene interaction analyses were carried out using a dominant model for genotypes.

The analyses of logistic regression were done for the unadjusted (model I) and adjusted models (models II and III). Models II and III were adjusted for known risk factors for CRC: [31, 32] age, sex, BMI, PE level, smoking status, the intensity of smoking (in current and past smokers), socio-economic level (DI) and health status (PRM), energy intake, dietary fibre, ethanol intake, and antiplatelet and anticoagulants use. The reference categories were those that, according to the literature, have a lower CRC risk. For the BMI variable, normal weight was considered as the reference category, and underweight was included as a separate category. We included participants with missing data for the covariates as a separate category.

Quantitative covariates such as intensity of smoking (cigarettes/day) were dichotomised by mean or median, according to the normality test. We used the cut-off of Romaguera et al. [33] to create two PE levels expressed in min/day of cycling/sports: sedentary-light (< 15 min/day) and moderate-vigorous (≥ 15 min/day). Age was dichotomised using the same age ranges that were used in the sample selection process (50–59 years old vs. 60–69 years old). Qualitative ones, such as DI and PRM were dichotomised considering the distribution of frequencies to obtain similar sample sizes for each category (DI, quintile 1–3 vs. quintile 4–5; PRM, level 3–4 vs. level 1–2). Energy, dietary fibre, and ethanol intake were included as quantitative variables in the adjusted models.

In model II, dietary compound intake or SNPs were included separately, whereas model III was only used in the analysis of dietary compound intake and, in this case, all the compounds were included at the same time. In addition, to study the possible association between the intake of betaine and total choline, and the CRC risk, model II (adjusted model) of the regression analysis was also applied, including folate as covariate (these data are shown in text form in the Results section). The significance level was corrected using a Bonferroni correction by dividing the standard *P* value (two-tailed) (α = 0.05) by the total number of SNPs analysed (*n* = 10), assuming alpha was equal to 0.005 (α = 0.05/10).

## Results

### Colorectal cancer risk according to nutrient intake

The intakes of folate, vitamins B_2_ and B_6_, Met, choline, and betaine were significantly higher in controls than those in cases (*P* < 0.001), whereas the consumption of vitamin B_12_ and alcohol was higher in cases than controls (*P* < 0.001) (Table 1). The ORs for CRC risk by the intake of nutrients are presented in Table 2. The adjusted ORs for CRC risk decreased with higher intakes of choline and betaine (*P* < 0.005). Although in the case of choline, the association was only significant in the unadjusted model. These results were confirmed in the subgroup of cases with distal tumour location (Table S2). In the total sample, after further adjustment for folate, moderate intake of betaine remained associated with a reduced risk of CRC (Model II including folate as a covariate, OR_T2 vs. T1_ = 0.36, 95% CI: 0.20–0.65, *P* = 0.001; OR_T3 vs. T1_ = 0.20, 95% CI: 0.09–0.40, *P* < 0.01).

**Table 1:** Daily intake of nutrients and alcohol in cases and control studied

Nutrient and ethanol intake, mean (SD)	Cases (*n* = 229)	Controls (*n* = 229)	*P* ^a^
Folate, µg/day	266.5 (81.6)	270.9 (77.8)	**< 0.001**
Vitamin B_2_, mg/day	1.5 (0.5)	1.6 (0.5)	**< 0.001**
Vitamin B_6_, mg/day	1.8 (0.5)	1.9 (0.6)	**< 0.001**
B_12_, µg/day	5.0 (1.8)	4.9 (1.6)	**< 0.001**
Met, mg/day	1783.0 (655.9)	1884.0 (756.9)	**< 0.001**
Total choline, mg/day	136.6 (87.3)	165.2 (86.9)	**< 0.001**
Betaine, mg/day	111.5 (54.8)	149.6 (61.5)	**< 0.001**
Ethanol, g/day	8.3 (8.1)	7.4 (8.9)	**< 0.001**

Abbreviations: Met, methionine; SD, standard deviation

^a^Wilcoxon test. A value of *P* < 0.005 was considered significant after the Bonferroni correction (assuming alpha was equal to 0.005, α = 0.05/10). Significant results are highlighted in bold

**Table 2:** Colorectal cancer risk according to nutrient intake

Nutrient intake^a^		Model I^b^		Model II^c^		Model III^d^	
	Cases/Controls, n	OR (95% CI)	*P* ^e^	OR (95% CI)	*P* ^e^	OR (95% CI)	*P* ^e^
Folate							
T1 T2 T3	81/83 80/71 68/75	1.00 1.15 (0.74–1.79) 0.94 (0.61–1.46)	- 0.539 0.791	1.00 1.21 (0.64–2.33) 1.33 (0.54–3.22)	- 0.563 0.640	1.00 1.11 (0.49–2.54) 1.82 (0.60–5.57)	- 0.792 0.299
Vitamin B_2_							
T1 T2 T3	85/83 91/71 53/75	1.00 1.24 (0.81–1.89) 0.64 (0.39–1.04)	- 0.318 0.072	1.00 0.93 (0.51–1.70) 0.45 (0.20–0.98)	**-** 0.798 0.048	1.00 1.19 (0.51–2.72) 0.56 (0.18–1.77)	- 0.704 0.324
Vitamin B_6_							
T1 T2 T3	68/67 99/85 62/77	1.00 1.11 (0.72–1.70) 0.77 (0.46–1.26)	- 0.639 0.297	1.00 0.76 (0.39–1.60) 0.53 (0.24–1.23)	- 0.477 0.141	1.00 0.61 (0.25–1.60) 0.60 (0.19–2.21)	- 0.310 0.454
Vitamin B_12_							
T1 T2 T3	70/73 74/80 85/76	1.00 0.95 (0.60–1.48) 1.21 (0.76–1.91)	- 0.809 0.429	1.00 0.95 (0.52–1.83) 1.15 (0.51–2.72)	- 0.895 0.699	1.00 0.85 (0.40–1.88) 1.73 (0.63–4.79)	- 0.686 0.287
Met							
T1 T2 T3	72/76 89/78 68/75	1.00 1.23 (0.77–1.95) 0.96 (0.60–1.53)	- 0.383 0.864	1.00 1.13 (0.62–2.11) 0.51 (0.24–0.99)	- 0.690 0.049	1.00 0.96 (0.47-2.00) 0.42 (0.22–0.92)	**-** 0.922 0.026
Choline							
T1 T2 T3	105/75 75/77 49/77	1.00 0.72 (0.46–1.11) 0.44 (0.27–0.72)	- 0.134 **0.001**	1.00 0.60 (0.30–0.95) 0.53 (0.29–1.06)	- 0.030 0.060	1.00 0.64 (0.32–1.25) 0.84 (0.38–1.84)	- 0.183 0.656
Betaine							
T1 T2 T3	150/77 41/75 38/77	1.00 0.27 (0.17–0.46) 0.28 (0.17–0.46)	- **< 0.001** **< 0.001**	1.00 0.30 (0.18–0.64) 0.21 (0.10–0.45)	- **0.001** **< 0.001**	1.00 0.31 (0.14–0.72) 0.21 (0.10–0.40)	- **0.003** **< 0.001**

Abbreviations: CI, confidence interval; Met, methionine; OR, odds ratio; T, tertile

^a^Nutrient intake was categorised into tertiles based on the distribution in the control group, taking into account sex differences when they were significant. Specifically, different cutoff points were applied to estimate tertiles in men and women when significant sex differences were identified. Tertiles of nutrient intake: folate (µg/day), for males, T1 ≤ 220.0, T2 220.1–289.0, T3 > 289.0, and females, T1 ≤ 245.0, T2 245.1–300.0, T3: > 300.0; vitamin B_2_ (mg/day), T1 ≤ 1.3, T2 1.4–1.7, T3 > 1.7; vitamin B_6_ (mg/day), T1 ≤ 1.5, T2 1.6-2.0, T3 > 2.0; vitamin B_12_ (µg/day), T1 ≤ 3.9, T2 4.0-5.3, T3 > 5.3; Met (mg/day), for males, T1 ≤ 1324.0, T2 1324.1–1985.0, T3 > 1985.0, and females, T1 ≤ 1564.0, T2 1564.1–2623.0, T3 > 2623.0; choline (mg/day), T1 ≤ 114.0, T2 114.1–190.0, T3 > 190.0; betaine (mg/day), T1 ≤ 119.0, T2 119.1–165.0, T3 > 165.0

^b^Model I, analysis was performed using crude conditional logistic regression

^c^Model II, analyses were performed using conditional logistic regression analysis adjusted for the following variables (reference categories are underlined): sex (women; men) age (50–59 y old, 60–69 y old), BMI (normal weight, overweight/obesity), physical exercise (< 15 min/day of cycling/sports, ≥ 15 min/day), smoking status (never, past/currently: smoker: ≤ 15 cigarettes/day, > 15 cigarettes/day), Deprivation Index (quintile 1–3, quintile 4–5), Predictive Risk Modelling (level 1–2, level 3–4), energy intake (kcal/day), dietary fibre (g/day), ethanol intake (g/day), antiplatelet (including non-steroidal anti-inflammatory drugs) and anticoagulants use (dichotomised variable, yes vs. no), including nutrients separately; participants with missing data for the confounding variables were included as a separate category for these variables

^d^Model III, model II including all the nutrients analysed

^e^A value of *P* < 0.005 was considered significant after the Bonferroni correction (assuming alpha was equal to 0.005, α = 0.05/10). Significant results are highlighted in bold

### Colorectal cancer risk according to polymorphism genotypes

The distribution of genotypes at SNPs selected in the CRC group and in the control group that deviated from the Hardy-Weinberg equilibrium is shown in the Supplementary Material (Table S3). The SNP that was not following the Hardy-Weinberg equilibrium in controls was rs1801394 (*P* < 0.05), however, this SNP showed no significance when the conservative Bonferroni method is used. None of the genotype frequencies for the SNPs analysed reached statistically significant differences between cases and controls, after the Bonferroni correction.

Supplementary Table S4 presents the associations between genotype variants and CRC risk. No significant association was found between the genotype for any SNP analysed and CRC risk, except a decreased risk of CRC among those with rs2424913-TT variant (OR_TT vs. CC_ = 0.56, 95% CI: 0.33–0.96, *P* < 0.05), even though this association was not significant after the Bonferroni correction.

### Colorectal cancer risk according to nutrient-gene interactions

Associations between SNP genotypes and CRC risk, stratified by dietary factors in unadjusted and adjusted models are shown in Supplementary Tables S5 and S6, respectively. A summary of all observed associations between folate metabolism-related nutrients and SNPs on CRC risk is provided in Table 3. In the unadjusted model, the rs1476413-CC genotype was associated with a decreased risk of CRC among individuals with high total choline intake (OR _T3 vs. T1_ = 0.29, 95% CI: 0.15–0.55) (*P*_interaction_ = 0.002). This result was also confirmed in the adjusted model, although in this case, *P*-value did not remain significant after applying the Bonferroni correction *(P*_interaction_ = 0.012). Moreover, in the adjusted model, the rs17824591-GG genotype was associated with an increased risk of CRC among individuals with moderate vitamin B_12_ intake (OR _T2 vs. T1_ = 2.48, 95% CI: 1.04-5.00) (*P*_interaction_ = 0.003). Although, in the unadjusted model, these results were not confirmed in the subgroup of cases with distal tumour location (Table S7); in the adjusted model, the rs1476413-CC genotype was associated with a decreased risk of CRC among subjects with high betaine intake (OR _T3 vs. T1_ = 0.22, 95% CI: 0.09–0.49) (*P*_interaction_ = 0.004) (Table S8).

**Table 3:** Summary of observed associations between nutrients and SNPs on colorectal cancer risk

Model	Genes	SNP ID (rs), genotypes	Nutrients (tertile)	CRC risk (P_interaction_^a^)
Unadjusted^b^	*MTHFR* (Chr 1)	rs1476413-CC	Choline (T3)	↓ (**0.002**)
		rs1801131-TT	Choline (T3)	↓ (0.019)
Adjusted^c^	*MTHFR* (Chr 1)	rs1476413-CC	Choline (T3)	↓ (0.012)
	*MTHFD1* (Chr 14)	rs17824591-GG	Vitamin B_12_ (T2)	↑ (**0.003**)

Abbreviations: C, cytosine; Chr, chromosome; CRC, colorectal cancer; G, guanine; *MTHFD*, methylene tetrahydrofolate dehydrogenase; *MTHFR*, methylene tetrahydrofolate reductase; rs, reference single nucleotide polymorphism; SNP, single nucleotide polymorphism; T, thymine; T1, first tertile; T2, second tertile; T3, third tertile

Tertiles of nutrient intake: vitamin B_12_ (µg/day), T1 ≤ 3.9, T2 4.0-5.3, T3 > 5.3; total choline (mg/day), T1 ≤ 114.0, T2 114.1–190.0, T3 > 190.0; betaine (mg/day), T1 ≤ 119.0, T2 119.1–165.0, T3 > 165.0. ↑, increased risk; ↓, decreased risk

^a^A value of *P* < 0.005 was considered significant after the Bonferroni correction. Significant results are highlighted in bold

^b^Analysis was performed using crude conditional logistic regression

^c^Analyses were performed using conditional logistic regression analysis adjusted for the following variables (reference categories are underlined): sex (women; men) age (50–59 y old, 60–69 y old), BMI (normal weight, overweight/obesity), physical exercise (< 15 min/day of cycling/sports, ≥ 15 min/day), smoking status (never, past/currently: smoker: ≤ 15 cigarettes/day, > 15 cigarettes/day), Deprivation Index (quintile 1–3, quintile 4–5), Predictive Risk Modelling (level 1–2, level 3–4), energy intake (kcal/day), dietary fibre (g/day), ethanol intake (g/day), antiplatelet (including non-steroidal anti-inflammatory drugs) and anticoagulants use (dichotomised variable, yes vs. no), including SNPs separately; participants with missing data for the confounding variables were included as a separate category for these variables

Additionally, even if the remaining combination of SNPs and nutrient intakes did not show any significant interaction for CRC risk, the following variants were associated with a decreased risk of CRC among individuals with moderate-high betaine and/or total choline intake, in both the unadjusted and adjusted models (Tables S5 and S6): *DNMT3B* (rs2424913, rs406193), *DNMT1* (rs2228612), *MTHFR* (rs1801131, rs1801133), *MTHFD1* (rs8003379, rs17824591), and *Met synthase reductase* (rs1801394, rs10380). These results were confirmed in the subgroup of cases with distal tumour location (Table S7 and S8).

### Colorectal cancer risk according to nutrient-lifestyle interactions

On the other hand, the combined effects of nutrient intake and lifestyle factors (PE, smoking, and alcohol consumption) on CRC risk were also examined. Individuals who reported both a low and a moderate-high level of PE (OR _T3 vs. T1_ = 0.27, 95% CI: 0.13–0.66; OR _T3 vs. T1_ = 0.12, 95% CI: 0.07–0.44, respectively) and a low or no alcohol consumption (OR _T3 vs. T1_ = 0.34, 95% CI: 0.23–0.61) and had high betaine intake showed a low CRC risk, even if no significant interactions were found (Table 4).

**Table 4:** Associations between lifestyle factors and colorectal cancer risk, stratified by dietary factors (adjusted model)^a^

Lifestyle factors, stratified by dietary factors	Nutrient intake									*P*_interaction_^b^
	T1			T2			T3			
	Cases/ Controls, n	OR (95%CI)^a^	*P* ^2^	Cases/ Controls, n	OR (95%CI)^a^	*P* ^2^	Cases/ Controls, n	OR (95%CI)^a^	*P* ^2^	
Folate										
Physical exercise										
≥15 min/day <15 min/day	32/20 49/63	1.00 0.89 (0.34–2.22)	0.760	26/15 54/56	1.45 (0.44–4.78) 0.95 (0.33–2.28)	0.522 0.916	21/17 47/58	1.63 (0.42–6.49) 1.07 (0.34–3.41)	0.498 0.892	0.938
Smoking status										
Never Ever	23/35 58/48	1.00 1.42 (0.31–3.48)	0.464	22/25 58/46	1.33 (0.42–3.99) 1.51 (0.60–3.81)	0.539 0.391	24/29 44/46	1.56 (0.46–5.16) 1.81 (0.62–5.54)	0.483 0.322	0.233
Alcohol consumption^c^										
Abstemious/low risk High risk	70/73 11/10	1.00 1.01 (0.31–3.30)	0.987	68/63 12/8	1.34 (0.66–2.66) 0.80 (0.24–2.65)	0.450 0.650	61/68 7/7	1.35 (0.53–3.35) 1.32 (0.35–6.01)	0.543 0.660	0.891
Vitamin B_2_										
Physical exercise										
≥15 min/day <15 min/day	34/21 51/62	1.00 0.72 (0.33–1.61)	0.389	31/17 60/54	0.83 (0.29–2.52) 0.69 (0.30–1.60)	0.719 0.350	14/14 39/61	0.55 (0.16–1.89) 0.25 (0.10–0.90)	0.298 0.031	0.836
Smoking status										
Never Ever	28/33 57/50	1.00 0.70 (0.32–1.55)	0.337	22/29 69/42	0.34 (0.14–0.98) 1.03 (0.44–2.41)	0.049 0.975	19/27 34/48	0.36 (0.11–1.01) 0.35 (0.13-1.00)	0.051 0.050	0.400
Alcohol consumption^c^										
Abstemious/low risk High risk	75/74 10/9	1.00 0.61 (0.18–2.23)	0.438	76/64 15/7	0.81 (0.30–1.47) 1.93 (0.53–8.32)	0.512 0.422	48/66 5/9	0.47 (0.20–1.12) 0.20 (0.09–0.79)	0.073 0.023	0.350
Vitamin B_6_										
Physical exercise										
≥15 min/day <15 min/day	28/16 40/51	1.00 0.65 (0.23–1.62)	0.359	34/22 65/63	0.65 (0.26–1.91) 0.55 (0.20–1.46)	0.432 0.233	17/14 45/63	0.48 (0.07–2.03) 0.37 (0.14–1.09)	0.291 0.070	0.885
Smoking status										
Never Ever	16/28 52/39	1.00 1.02 (0.41–2.64)	0.964	33/29 66/56	0.75 (0.25–2.14) 0.83 (0.32–2.25)	0.613 0.712	20/32 42/45	0.38 (0.12–1.21) 0.68 (0.22–2.10)	0.105 0.479	0.299
Alcohol consumption^c^										
Abstemious/low risk High risk	62/60 6/7	1.00 0.57 (0.15–2.30)	0401	82/76 17/9	0.70 (0.37–1.40) 1.10 (0.32–3.60)	0.301 0.885	55/7 7/9	0.50 (0.20–1.27) 0.33 (0.12–1.30)	0.187 0.103	0.389
Vitamin B_12_										
Physical exercise										
≥15 min/day <15 min/day	27/16 43/57	1.00 0.41 (0.12–1.09)	0.060	25/19 49/61	0.41 (0.12–1.33) 0.51 (0.19–1.33)	0.135 0.166	27/17 58/59	0.70 (0.20–2.09) 0.61 (0.18–2.05)	0.419 0.409	0.720
Smoking status										
Never Ever	21/26 49/47	1.00 1.09 (0.43–2.77)	0.890	23/35 51/45	0.73 (0.29–2.02) 1.11 (0.43–2.76)	0.539 0.869	25/28 60/48	0.99 (0.31–3.20) 1.22 (0.41–3.65)	0.991 0.712	0.829
Alcohol consumption^c^										
Abstemious/low risk High risk	63/65 7/8	1.00 0.52 (0.13–1.81)	0.282	61/74 13/6	0.88 (0.46–1.59) 1.70 (0.44–6.67)	0.577 0.459	75/65 10/11	1.10 (0.47–2.67) 1.02 (0.22–2.71)	0.802 0.912	0.353
Met										
Physical exercise										
≥15 min/day <15 min/day	20/18 52/58	1.00 1.16 (0.43–3.09)	0.701	36/20 53/58	1.34 (0.48–3.71) 1.21 (0.47–3.31)	0.572 0.680	23/14 45/61	0.90 (0.20–3.80) 0.52 (0.18–1.33)	0.878 0.177	0.740
Smoking status										
Never Ever	21/31 51/45	1.00 1.08 (0.44–2.60)	0.879	31/31 58/47	1.20 (0.44–3.18) 1.25 (0.46–3.15)	0.755 0.643	17/27 51/48	0.41 (0.16–1.10) 0.65 (0.27–1.73)	0.079 0.369	0.477
Alcohol consumption^c^										
Abstemious/low risk High risk	63/68 9/8	1.00 0.95 (0.32–3.45)	0.925	75/71 14/7	1.12 (0.60–2.23) 1.10 (0.29–3.37)	0.707 0.822	61/65 7/10	0.49 (0.30–1.09) 0.42 (0.10–1.73)	0.080 0.221	0.798
Choline										
Physical exercise										
≥15 min/day <15 min/day	37/13 68/62	1.00 0.60 (0.20–1.81)	0.364	29/20 46/57	0.59 (0.16–1.80) 0.32 (0.13–0.88)	0.330 0.029	13/19 36/58	0.25 (0.10–1.13) 0.40 (0.12–1.08)	0.070 0.069	0.710
Smoking status										
Never Ever	28/31 77/44	1.00 1.16 (0.53–2.69)	0.678	23/30 52/47	0.49 (0.17–1.54) 0.74 (0.39–1.72)	0.232 0.450	18/28 31/49	0.60 (0.22–1.52) 0.66 (0.21–1.56)	0.251 0.269	0.998
Alcohol consumption^c^										
Abstemious/low risk High risk	92/72 13/3	1.00 2.31 (0.53–11.97)	0.361	65/64 10/13	0.66 (0.36–1.16) 0.27 (0.11–0.82)	0.163 0.021	42/68 7/9	0.61 (0.26–1.05) 0.79 (0.21–2.89)	0.077 0.730	0.290
Betaine										
Physical exercise^c^										
≥15 min/day <15 min/day	57/15 93/62	1.00 0.76 (0.33–1.78)	0.508	13/18 28/57	0.48 (0.15–1.69) 0.22 (0.09–0.55)	0.242 **0.002**	9/19 29/58	0.12 (0.07–0.44) 0.27 (0.13–0.66)	**0.002** **0.004**	0.218
Smoking status^c^										
Never Ever	45/30 105/47	1.00 1.51 (0.66–3.48)	0.330	10/32 31/43	0.27 (0.09–0.83) 0.56 (0.22–1.46)	0.022 0.235	14/27 24/50	0.50 (0.18–1.38) 0.26 (0.10–0.66)	0.184 0.005	0.351
Alcohol consumption^c^										
Abstemious/low risk High risk	131/72 19/5	1.00 1.62 (0.37–6.61)	0.558	37/65 4/10	0.41 (0.20–0.78) 0.36 (0.08–0.93)	0.009 0.044	6/13 32/64	0.34 (0.23–0.61) 0.31 (0.10–0.98)	**0.001** 0.048	0.298

Abbreviations: CI, confidence interval; Met, methionine; OR, odds ratio; T1, first tertile; T2, second tertile; T3, third tertile

Tertiles of nutrient intake: folate (µg/day), for males, T1 ≤ 220.0, T2 220.1–289.0, T3 > 289.1, and females, T1 ≤ 245.0, T2 245.1–300.0, T3: > 300.0; vitamin B_2_ (mg/day), T1 ≤ 1.3, T2 1.4–1.7, T3 > 1.70; vitamin B_6_ (mg/day), T1 ≤ 1.5, T2 1.6-2.0, T3 > 2.0; vitamin B_12_ (µg/day), T1 ≤ 3.9, T2 4.0-5.3, T3 > 5.3; Met (mg/day), for males, T1 ≤ 1324.0, T2 1324.1–1985.0, T3 > 1985.0, and females, T1 ≤ 1564.0, T2 1564.1–2623.0, T3 > 2623.0; choline (mg/day), T1 ≤ 114.0, T2 114.1–190.0, T3 > 190.0; betaine (mg/day), T1 ≤ 119.0, T2 119.1–165.0, T3 > 165.0

^a^Analyses were performed using conditional logistic regression analysis adjusted for the following variables (reference categories are underlined): sex (women; men) age (50–59 y old, 60–69 y old), BMI (normal weight, overweight/obesity), physical exercise (< 15 min/day of cycling/sports, ≥ 15 min/day), smoking status (never, past/currently: smoker: ≤ 15 cigarettes/day, > 15 cigarettes/day), Deprivation Index (quintile 1–3, quintile 4–5), Predictive Risk Modelling (level 1–2, level 3–4), energy intake (kcal/day), dietary fibre (g/day), ethanol intake (g/day), antiplatelet (including non-steroidal anti-inflammatory drugs) and anticoagulants use (dichotomised variable, yes vs. no), including lifestyle factors separately; participants with missing data for the confounding variables were included as a separate category for these variables. In the analyses of the variables physical exercise, smoking status, and alcohol consumption these same variables were excluded as an adjustment variable

^*b*^A value of *P* < 0.005 was considered significant after the Bonferroni correction. Significant results are highlighted in bold

^c^Conditional exact logistic regression

## Discussion

The present study aimed to determine the association between dietary factors and genetic variants related to the FOCM and CRC risk, as well as possible nutrient-gene interactions. Moreover, the combined effects of nutrient intake and lifestyle factors (PE, smoking, and alcohol consumption) on CRC risk were also examined. Our results suggest that betaine intake and interactions between some dietary factors and variants in *MTHFR* and *MTHFD1* genes have an influence on CRC risk in the population studied. The results were confirmed in the subgroup with distal tumour location. On the other hand, no significant interactions were observed between nutrient intake and lifestyle factors on CRC risk.

As we mentioned in the introduction section, to date, few epidemiologic studies have examined the association between betaine and cancer risk, and those who have studied this possible relationship have obtained inconsistent results. Several researchers found an inverse association between betaine intake and breast cancer risk [34]. However, other studies found no evidence that higher intakes of this nutrient reduced the risk of breast cancer [35]. Some studies have reported that a higher intake of betaine was associated with a reduced risk of lung cancer [36], whereas no association was found for epithelial ovarian cancer [37]. It has been suggested that the underlying mechanisms by which a high intake of betaine would reduce the risk of some cancers would be similar to those of folate. Betaine can donate the methyl group to homocysteine as does folate, although the donation of the methyl group by betaine is limited to the liver and the kidney [34]. A high intake of betaine could help prevent the adverse effect resulting from hypomethylation of DNA or restore DNA repair mechanism, and therefore, would lead to reduced cancer risk [36].

Inconsistent results were also observed on the relationship between betaine intake and CRC risk. The Health Professionals Follow-up Study conducted in the United States [38] and an investigation carried out in a Chinese population [39] have examined this possible association and in both studies, no association was found. This result has been attributed, in part, to the fact that the levels of this nutrient would not be critical in folate-nourished populations, because folate and choline metabolic pathways are highly interrelated, and betaine is derived from choline and increase in response to a higher choline intake [40].

Our study, like another case-control study, where plasma betaine was analysed, [41] confirmed the inverse association between betaine intake and CRC risk, even among subjects with an average total folate intake below population recommendations and below the intakes recorded in other studies, such as the Health Professionals Follow-up Study mentioned above, [38] with a total folate intake from diet and supplements of 479–858 µg/day in the total sample. In the present study, the average folate intake was 270.9 µg/day among controls and therefore the intake level of folate was not very high.

Nevertheless, the few published on the association between betaine intake and CRC risk are confusing. Neither the Health Professionals Follow-up Study [38] nor the one carried out by Lu et al. [39] found significant associations for betaine intakes. These discrepancies could be due to differences in the characteristics of participants, the intake and status of other nutrients involved in FOCM, and the design type. In summary, in the Health Professionals Follow-up Study, [38] the participants were US male health professionals aged 40 to 75 years, and in Lu et al.´s study, [39], Chinese males and females aged 30 to 75 years. The folate intake in the total sample of Lee et al.´ study [38] was of 479–858 µg/day, whereas, in the control sample of Lu et al.´s study [39] was 240.3 µg/d. Lee et al.´ study [38] is a prospective cohort study, whereas, Lu et al.´s study [39] is a case-control study.

Regarding the possible associations between genetic variants related to the FOCM and CRC risk, in the current study, a genotype in *DNMT3B* (rs2424913) was related to CRC risk, even though this relationship was not significant after the Bonferroni correction. Other authors did not find a close correlation between this genetic variant and the development of CRC among the Chinese population [42]. As regards potential nutrient-gene interactions on CRC risk, rs1476413, and rs17824591 exhibited significant nutrient-gene interactions with total choline and vitamin B_12_ intakes, respectively. These findings suggest that these variants in *MTHFR* and *MTHFD1* genes interact with dietary factors to modify the risk for CRC. It should be noted that the mechanism by which choline intake/status affects DNA integrity is not entirely clear but may be through effects on mitochondrial membrane integrity and oxidative stress [43]. Regarding vitamin B_12_, the underlying mechanism by which a high intake of this vitamin would reduce CRC risk could be related to tumour methylation, as other authors have pointed out in colon cancer [44].

In another case-control study, however, no interactions were found between the *MTHFR* rs1476413 SNP and dietary factors (including folate and Met) on CRC risk, although other *MTHFR* and *MTHFD1* SNPs exhibited gene-diet interactions with Met intake [28]. The lack of data on the possible interaction between the aforementioned SNPs, and their interaction with betaine, makes it difficult to compare our results to other studies.

Most studies assessing *MTHFR* and CRC risk have focused on the rs1801133 SNP. The variant allele in this SNP causes an increase in thermolability of the MTHFR enzyme [45] which is associated with decreased plasma folate and increased plasma homocysteine [46]. The potential influence of MTHFR activity on DNA methylation and the availability of uridylates and thymidylates for DNA synthesis and repair makes *MTHFR* an attractive candidate for a cancer-predisposing gene. Even if other studies found an association between this SNP and the CRC risk and interaction with Met, [42] in the present study no significant association was found between these factors. Furthermore, few studies have assessed polymorphisms in *MTHFD1* in relation to risk for CRC [7, 42]. Nevertheless, those SNPs (rs2295638, rs2236225) in which they found interaction with nutrients, specifically with Met [7, 42, 47] were not any of those analysed in the present study.

In any case, even if in the present study no more nutrient-gene interactions were detected, individuals with moderate-high betaine and/or total choline intakes showed a decreased risk, for all genotypes analysed, both in the total sample and in the subgroup with distal tumour location. Finally, concerning interactions between nutrient intakes and lifestyle factors, although no interactions were found, individuals who reported low or no alcohol consumption, and had moderate-high levels of PE and high betaine intake showed the lowest risk of CRC. Even though no data have been found in the literature on these interactions, our findings agree with previous studies about the interaction effects of folate status and lifestyle factors on CRC risk [48].

The main strength of this study compared to other previous published [49] is that colonoscopy was used as a diagnosis criterion to identify both cases and controls to avoid false positives and negatives. To our knowledge, to date, only one other study of the association between diet and CRC risk has been published, in which it was confirmed that controls were free of the disease through colonoscopy [50]. Another strength is the fact that information is provided based on a standardised protocol including not only dietary factors but also other possible determinants of CRC such as health determinants and weight status among others. However, some limitations should be mentioned. First, recall bias is also of concern in case-control studies. Second, the small sample size makes it difficult to detect possible associations and nutrient-gene and nutrient-lifestyle interactions and disease risk, since some genotypes and categories according to lifestyle factors showed low frequencies in our population.

Another disadvantage of the small sample size is that they can produce false-positive results; to avoid it, the Bonferroni correction was used. Third, self-reported data could be subject to measurement errors and the problem of food omissions due to memory failure and under-reporting of unhealthy habits among disease subjects. However, previous validation studies indicate that the self-reported dietary information is reported with sufficient accuracy for use in epidemiology analysis [51]. Fourth, although the FFQ used to collect information on dietary intake in the present study has been validated among people who lived in the same region, this validation did not include specific nutrients such as betaine or total choline, but it included the main food sources of these methyl-donors. So, the possible measurement errors are most likely non-differential and thus do not explain the inverse associations observed in our study.

Fifth, even though data on lifestyles (including diet) were recorded retrospectively—that is, the questions referred to behaviours before participating in the BCSP—it should be also noted that dietary changes are usually modest after participating in the BCSP due to a lack of information and personalised advice [52]. In addition, adults generally maintain relatively stable eating habits for a long time [53]. Therefore, the results of this study are unlikely to be greatly affected by potential changes in eating. Sixth, although we have adjusted for several confounding factors, some residual confounding may result from the misclassification of those variables and confounding by unmeasured variables. Finally, to avoid selection bias of controls, we obtained controls from the same BCSP and in the same period as cases. It should also worth noting that we have compared our results with those of the Health Professionals Follow-up Study, [38] that only includes male participants.

In conclusion, the present study suggests that high betaine intake is associated with decreased risk of CRC among the population studied. Moreover, our results support the existing hypothesis of genetic-nutrient interactions in colorectal carcinogenesis. Total choline and vitamin B_12_ intake, and the SNPs rs1476413 and rs17824591 may be related, respectively, to CRC risk in this population. High total choline intake together with the *MTHFR* rs1476413-CC genotype reduces CRC risk, whereas moderate vitamin B_12_ intake together with the *MTHFD1* rs17824591-GG genotype increases CRC risk.

Further studies are necessary to confirm these associations and understand in depth their role in colorectal carcinogenesis, including participants under 50 years old. Understanding the interaction between nutrition and genetic variation can be useful to distinguish between individuals who will and who will not benefit from diet intervention strategies.

## Electronic supplementary material

Below is the link to the electronic supplementary material.

Supplementary Material 1

## Data Availability

The data that support the findings of this study are available from the corresponding author upon reasonable request.

## Abbreviations

1CM: One-carbon metabolism
BCSP: Population-based bowel cancer screening program
CI: Confidence interval
CRC: Colorectal cancer
DI: Deprivation index
DNMT: DNA methyltransferases
FFQ: Food frequency questionnaire
FOCM: Folate-mediated one-carbon metabolism
Met: Methionine
MTHFD1: Methylene tetrahydrofolate dehydrogenase 1
MTHFR: Methylene tetrahydrofolate reductase
OR: Odds ratio
PE: Physical exercise
PRM: Predictive risk modelling
SD: Standard deviation
SNP: Single nucleotide polymorphism
T: Tertile
