# Supplementary material for: Interactions between folate metabolism-related nutrients and polymorphisms on colorectal cancer risk: a case-control study in the Basque country
Source: Eur J Nutr. 2024 Apr 23;63(5):1681–93. doi: 10.1007/s00394-024-03371-5 (PMC11329606; doi:10.1007/s00394-024-03371-5)
Supplement: Supplementary file 1 — Supplementary Material 1 [file 394_2024_3371_MOESM1_ESM.docx]

**Interactions between folate metabolism-related nutrients and polymorphisms on colorectal cancer risk: a case-control study in the Basque Country**

Sara Corchero-Palacios, Iker Alegria-Lertxundi, Marian M. de Pancorbo, Marta Arroyo-Izaga*

*Correspondence: Marta Arroyo-Izaga, BIOMICs Research Group, Microfluidics & BIOMICs Cluster, Department of Pharmacy and Food Sciences, University of the Basque Country UPV/EHU, Bioaraba, BA04.03, 01006 Vitoria-Gasteiz, Araba/Álava, Spain. [marta.arroyo@ehu.eus](mailto:marta.arroyo@ehu.eus)

**Supplementary Table S1** General characteristics of the sample studied.

| %^a^ or mean(SD) | Cases  (n=308) | Controls  (n=308) | *P* |
| --- | --- | --- | --- |
| Sex, % of men | 66.2 | 66.2 |  |
| Age, years, mean(SD) | 61.5(5.2) | 61.1(5.5) | 0.093 |
| BMI, kg/m^2^, mean (SD) | 27.5(4.4) | 26.8(4.4) | 0.049 |
| Ov/Ob^b^, % | 67.5 | 58.1 | 0.015 |
| Underweight, % | 6.5 | 7.8 | 0.185 |
| Physical activity level, % |  |  |  |
| < 15 min/day of cycling/sports | 79.2 | 65.9 |  |
| ≥ 15 min/day of cycling/sports | 20.8 | 34.1 | **<0.001** |
| Smoking status, % |  |  |  |
| Never | 28.6 | 39.1 |  |
| Past/current | 71.2 | 60.9 | **0.004** |
| Smoking intensity, % |  |  |  |
| ≤ 15 cigarettes/day | 50.7 | 33.1 |  |
| > 15 cigarettes/day | 49.3 | 66.9 | **0.004** |
| Ethanol intake, g/day, mean(SD) | 8.3(8.1) | 7.4(8.9) | **<0.001** |
| Drugs, %  Antiplatelet (including non-steroidal anti-inflammatory drugs) and anticoagulants | 19.8 | 15.0 | 0.085 |
| DI, % |  |  |  |
| Q_1-3_ | 71.4 | 68.9 |  |
| Q_4-5_ | 28.6 | 31.1 | **<0.001** |
| PRM, % |  |  |  |
| L_1-2_ | 15.7 | 13.5 |  |
| L_3-4_ | 84.3 | 86.5 | **<0.001** |
| Cancer staging^c^, % |  |  |  |
| I | 57.1 |  |  |
| II | 14.9 |  |  |
| III | 24.0 |  |  |
| IV | 3.9 |  |  |
| Cancer location^d^, % |  |  |  |
| Distal | 76.0 |  |  |
| Proximal | 24.0 |  |  |
| Tumour degree of differentiation^e^, % |  |  |  |
| Low-grade | 94.7 |  |  |
| High-grade | 5.3 |  |  |
| Tumour treatment, % |  |  |  |
| Surgical resection | 73.7 |  |  |
| Chemotherapy | 34.1 |  |  |
| Chemotherapy and radiation | 6.8 |  |  |

Abbreviations: BMI, body mass index; DI, deprivation index; L, level; Ov/Ob, overweight/obesity; PRM, predictive risk modelling; Q, quintile; SD, standard deviation.

^a^Valid percentages.

^b^BMI was classified according to the WHO criteria for those under 65 years of age (World Health Organization. *Obesity: preventing and managing the global epidemic: report of a WHO consultation, 2000*. Accessed December 5, 2019. <https://apps.who.int/iris/handle/10665/42330>) and according to the criteria proposed by Silva Rodrigues *et al.* for those 65 and older (Silva Rodrigues RA, Martinez Espinosa M, Duarte Melo C, Rodrigues Perracini M, Rezende Fett WC, Fett CA. New values anthropometry for classification of nutritional status in the elderly. *J Nutr Health Aging.* 2014;18(7):655-661. <https://doi.org/10.1007/s12603-014-0451-2>).

^c^Edge SB, Compton CC. The American Joint Committee on Cancer: the 7th edition of the AJCC cancer staging manual and the future of TNM. *Ann Surg Oncol.* 2010;17:1471-1474. <https://doi.org/10.1245/s10434-010-0985-4>.

^d^Distal, tumours located in the while cecum, ascending, and hepatic flexure; Proximal, tumours located in the splenic flexure, descending colon, and sigmoid colon of the colon.

^e^Low-grade cancer, cancer cells were well or moderately differentiated; High-grade cancer, cancer cells were poorly differentiated, anaplastic, or undifferentiated.

**Supplementary Table S2** Colorectal cancer risk according to nutrient intake in the distal colorectal cancer patients.

| Nutrient intake^a^ |  | Model I^b^ | | Model II^c^ | | Model III^d^ | |
| --- | --- | --- | --- | --- | --- | --- | --- |
|  | Cases/Controls, n | OR (95% CI) | *P*^e^ | OR (95% CI) | *P*^e^ | OR (95% CI) | *P*^e^ |
| Folate  T1  T2  T3 | 62/66  64/51  52/61 | 1.00  1.30(0.79-2.34)  0.93(0.57-1.52) | –  0.296  0.775 | 1.00  1.37(0.61-2.88)  1.03(0.36-2.79) | –  0.451  0.978 | 1.00  1.20(0.44-3.21)  1.53(0.40-5.79) | –  0.752  0.601 |
| Vitamin B_2_  T1  T2  T3 | 63/64  73/54  42/60 | 1.00  1.35(0.83-2.18)  0.66(0.38-1.15) | –  0.224  0.144 | 1.00  0.99(0.49-1.98)  0.31(0.10-0.90) | –  0.980  0.025 | 1.00  0.90(0.33-2.42)  0.39(0.11-1.59) | –  0.805  0.169 |
| Vitamin B_6_  T1  T2  T3 | 51/54  78/64  49/60 | 1.00  1.24(0.76-2.02)  0.85(0.48-1.49) | –  0.399  0.566 | 1.00  0.90(0.40-1.89)  0.54(0.22-1.55) | –  0.689  0.198 | 1.00  0.77(0.25-2.40)  0.63(0.13-2.98) | –  0.650  0.546 |
| Vitamin B_12_  T1  T2  T3 | 49/62  63/60  66/56 | 1.00  1.33(0.80-2.22)  1.55(0.89-2.67) | –  0.277  0.119 | 1.00  1.63(0.74-3.18)  1.29(0.45-3.24) | –  0.207  0.572 | 1.00  1.01(0.50-2.72)  1.44(0.44-4.78) | –  0.976  0.502 |
| Met  T1  T2  T3 | 54/59  71/63  53/56 | 1.00  1.25(0.74-2.11)  1.06(0.62-1.82) | –  0.407  0.839 | 1.00  1.33(0.70-2.70)  0.67(0.32-1.41) | –  0.426  0.162 | 1.00  0.95(0.68-2.00)  0.44(0.19-1.26) | –  0.901  0.125 |
| Choline  T1  T2  T3 | 84/59  56/61  38/58 | 1.00  0.65(0.39-1.07)  0.45(0.26-0.78) | –  0.091  **0.004** | 1.00  0.39(0.25-1.01)  0.60(0.31-1.34) | –  0.050  0.199 | 1.00  0.62(0.32-1.39)  0.96(0.42-2.16) | –  0.299  0.899 |
| Betaine  T1  T2  T3 | 114/55  32/60  32/60 | 1.00  0.23(0.13-0.43)  0.25(0.14-0.45) | –  **<0.001**  **<0.001** | 1.00  0.35(0.25-0.72)  0.23(0.12-0.51) | –  **0.001**  **0.001** | 1.00  0.30(0.13-0.75)  0.25(0.10-0.52) | –  **0.006**  **<0.001** |

Abbreviations: CI, confidence interval; Met, methionine; OR, odds ratio; T, tertile.

^a^Nutrient intake was categorised into tertiles based on the distribution in the control group (only those controls matched with distal colorectal cancer patients), taking into account sex differences when they were significant. Specifically, different cutoff points were applied to estimate tertiles in men and women when significant sex differences were identified. Tertiles of nutrient intake: folate (µg/day), for males, T1 < 219.0, T2 219.0- 288.0, T3 > 288.0, and females, T1 < 245.0, T2 245.0-300.0, T3: > 300.0; vitamin B_2_ (mg/day), T1 < 1.2, T2 1.2-1.6, T3 >1.6; vitamin B_6_ (mg/day), T1 <1.4, T2 1.4-2.1, T3 > 2.1; vitamin B_12_ (µg/day), T1 ≤3.8, T2 3.9-5.3, T3 >5.3; Met (mg/day), for males, T1 < 1322.0, T2 1322.0-1986.0, T3 > 1986.0, and females, T1 < 1565.0, T2 1565.0-2623.0, T3 >2623.0; choline (mg/day), T1 <114.0, T2 114.0-188.0, T3 >188.0; betaine (mg/day), T1 <117.0, T3 117.0-162.0, T3 >162.0.

^b^Model I, analysis was performed using crude conditional logistic regression.

^c^Model II, analyses were performed using conditional logistic regression analysis adjusted for the following variables (reference categories are underlined): sex (women, men) age (50-59 y old, 60-69 y old), BMI (normal weight, overweight/obesity), physical exercise (< 15 min/day of cycling/sports, ≥15 min/day), smoking status (never, past/currently: smoker: ≤ 15 cigarettes/day, > 15 cigarettes/day), Deprivation Index (quintile 1-3, quintile 4-5), Predictive Risk Modelling (level 1-2, level 3-4), energy intake (kcal/day), dietary fibre (g/day), ethanol intake (g/day), antiplatelet (including non-steroidal anti-inflammatory drugs) and anticoagulants use (dichotomised variable, yes vs no), including nutrients separately; participants with missing data for the confounding variables were included as a separate category for these variables.

^d^Model III, model II including all the nutrients analysed.

^e^A value of *P* < 0.005 was considered significant after the Bonferroni correction (assuming alpha was equal to 0.005, α = 0.05/10). Significant results are highlighted in bold.

**Supplementary Table S3** Genotype distribution and Hardy-Weinberg equilibrium of SNPs related with the activity of folate-dependent enzymes.

| SNP ID (rs),  genotypes | Controls  n(%)^a^ | HWE  *P*^b^ | Cases  n(%)^a^ | HWE  *P*^b^ | Diff.^c^  *P*^b^ |
| --- | --- | --- | --- | --- | --- |
| rs2424913     CC     CT     TT | 91(39.7)  97(42.4)  41(17.9) | 0.095 | 111(48.5)  91(39.7)  27(11.8) | 0.216 | 0.082 |
| rs406193     CC     CT     TT | 163(71.2)  56(24.5)  10(4.4) | 0.078 | 163(71.8)  58(25.6)  6(2.6) | 0.759 | 0.648 |
| rs2228612     TT     TC     CC | 211(92.5)  17(7.5)  0(0.0) | 0.559 | 201(88.2)  27(11.8)  0(0.0) | 0.342 | 0.153 |
| rs1476413     CC     CT     TT | 124(54.4)  84(36.8)  20(8.8) | 0.294 | 126(55.0)  90(39.3)  13(5.7) | 0.555 | 0.435 |
| rs1801131     TT     TG     GG | 109(47.8)  100(43.9)  19(8.3) | 0.555 | 117(51.3)  92(40.4)  19(8.3) | 0.879 | 0.722 |
| rs1801133     CC     CT     TT | 83(36.2)  114(49.8)  32(14.0) | 0.471 | 92(40.4)  103(45.2)  33(14.4) | 0.633 | 0.591 |
| rs8003379     AA     AC     CC | 125(54.6)  89(38.9)  15(6.6) | 0.875 | 127(55.9)  90(39.6)  10(4.4) | 0.229 | 0.634 |
| rs17824591     GG     GA     AA | 134(58.5)  81(35.4)  14(6.1) | 0.708 | 140(61.1)  82(35.8)  7(3.1) | 0.222 | 0.302 |
| rs1801394     GG     GA     AA | 66 (28.8)  99 (43.2)  64 (27.9) | 0.041 | 63 (27.6)  104 (45.6)  61 (26.8) | 0.186 | 0.887 |
| rs10380     CC     CT     TT | 179 (78.2)  49 (21.4)  1 (0.4) | 0.219 | 175 (76.8)  50 (21.9)  3 (1.3) | 0.787 | 0.693 |

Abbreviations: A, adenine; C, cytosine; G, guanine; HWE, Hardy-Weinberg equilibrium; rs, reference single nucleotide polymorphism; SNP, single nucleotide polymorphism; T, thymine.

^a^Valid percentages.

^b^A value of *P* < 0.005 was considered significant after the Bonferroni correction (assuming alpha was equal to 0.005, α = 0.05/10).

^c^Differences in genotype distribution between cases and controls (Fisher’s exact test).

**Supplementary Table S4** Association between polymorphism genotypes and colorectal cancer risk.

| SNP ID (rs),  genotypes | Model I^a^ | | | Model II^b^ | |
| --- | --- | --- | --- | --- | --- |
|  | Cases /  Controls, n | OR(95% CI) | *P^c^* | OR(95% CI) | *P*^c^ |
| rs2424913  Codominant     CC     CT     TT     Dominant     CC     CT+TT     Recessive     CC+CT     TT | 111/91  91/97  27/41    111/91  118/138    202/188  27/41 | 1.00  0.76(0.51–1.14)  0.56(0.33–0.96)    1.00  0.70(0.48–1.02)  1.00  0.64(0.39–1.06) | -  0.189  0.037    -  0.060    -  0.083 | 1.00  0.84(0.48–1.45)  0.55(0.25–1.15)    1.00  0.74(0.45–1.27)    1.00  0.60(0.30–1.22) | -  0.515  0.104    -  0.262    -  0.140 |
| rs406193     Codominant^d^     CC     CT  TT  Dominant  CC  CT+TT  Recessive^d^  CC+CT  TT | 163/163  58/56  6/10    163/163  64/66    221/219  6/10 | 1.00  1.04(0.68–1.59)  0.63(0.23–1.68)    1.00  0.96(0.63–1.45)    1.00  0.58(0.23–1.62) | -  0.912  0.333    -  0.833    -  0.332 | 1.00  1.18(0.63–2.11)  0.66(0.19–2.62)    1.00  1.09(0.62–1.90)    1.00  0.61(0.18–2.50) | -  0.608  0.542    -  0.788    1.00  0.490 |
| rs2228612     Codominant     TT     TC  CC  Dominant  TT  TC+CC  Recessive  TT+TC  CC | 201/211  27/17  0/0    201/211  27/17    228/228  0/0 | 1.00  1.71(0.89–3.31)  -    1.00  1.71(0.89–3.31)    1.00(omitted) | -  0.109  -    -  0.109 | 1.00  1.28(0.52–3.00)  -    1.00  1.27(0.56–2.99)    1.00(omitted) | -  0.601  -    -  0.599 |
| rs1476413     Codominant     CC     CT  TT  Dominant  CC  CT+TT  Recessive  CC+CT  TT | 126/124  90/84  13/20    126/124  103/104    216/208  13/20 | 1.00  1.06(0.70–1.62)  0.65(0.31–1.38)    1.00  0.98(0.65–1.46)    1.00  0.63(0.31–1.30) | -  0.779  0.261    -  0.918    -  0.213 | 1.00  1.24(0.69–2.23)  0.55(0.29–1.53)    1.00  1.09(0.63–1.87)    1.00  0.57(0.29–1.31) | -  0.480  0.272    -  0.275    -  0.180 |
| rs1801131     Codominant     TT     TG  GG  Dominant  TT  TG+GG  Recessive  TT+TG  GG | 117/109  92/100  19/19    117/109  111/119    209/209  19/19 | 1.00  0.84(0.56–1.26)  0.91(0.44–1.89)    1.00  0.85(0.58–1.26)  1.00  1.00(0.50–2.00) | -  0.809  0.412    -  0.429    -  1.000 | 1.00  0.72(0.43–1.25)  1.18(0.41–3.00)    1.00  0.81(0.44–1.25)    1.00  1.35(0.65–3.60) | -  0.219  0.720    -  0.300    -  0.560 |
| rs1801133     Codominant     CC     CT     TT  Dominant  CC  CT+TT  Recessive  CC+CT  TT | 92/83  103/114  33/32    136/146  92/83    195/197  33/32 | 1.00  0.83(0.56–1.22)  0.95(0.53–1.72)    1.00  0.85(0.58–1.24)    1.00  1.08(0.63–1.84) | -  0.338  0.871    -  0.389    -  0.786 | 1.00  0.72(0.44–1.20)  0.76(0.35–1.63)    1.00  0.73(0.45–1.22)    1.00  0.94(0.49–1.90) | -  0.199  0.470    -  0.201    -  0.877 |
| rs8003379     Codominant^d^     AA     AC  CC  Dominant  AA  AC+CC  Recessive^d^  AA+AC  CC | 127/125  90/89  10/15    127/125  100/104    217/214  10/15 | 1.00  0.99(0.68–1.45)  0.65(0.25–1.43)    1.00  0.93(0.64–1.35)    1.00  0.67(0.32–1.45) | -  0.919  0.313    -  0.706    -  0.293 | 1.00  0.90(0.72–1.73)  0.45(0.17–1.78)    1.00  0.90(0.54–1.48)    1.00  0.51(0.15–1.80) | -  0.912  0.280    -  0.670    -  0.280 |
| rs17824591     Codominant^d^     GG     GA  AA  Dominant  GG  GA+AA  Recessive^d^  GG+GA  AA | 140/134  82/81  7/14    140/134  89/95    222/215  7/14 | 1.00  0.97(0.65–1.43)  0.51(0.21–1.27)    1.00  0.89(0.60–1.31)    1.00  0.54(0.22–1.20) | -  0.832  0.140    -  0.549    -  0.140 | 1.00  1.09(0.62–1.89)  0.78(0.25–2.38)    1.00  1.07(0.64–1.79)    1.00  0.69(0.27–2.18) | -  0.651  0.650    -  0.805    -  0.505 |
| rs1801394     Codominant     GG     GA  AA  Dominant  GG  GA+AA  Recessive  GG+GA  AA | 63/64  104/99  61/66    63/66  165/163    167/165  61/64 | 1.00  1.11(0.72–1.71)  1.00(0.60–1.64)    1.00  1.07(0.71–1.60)    1.00  0.93(0.62–1.42) | -  0.990  0.651    -  0.756    -  0.751 | 1.00  1.34(0.72–2.30)  0.95(0.47-2.00)    1.00  1.21(0.66–2.22)    1.00  0.78(0.47–1.35) | -  0.345  0.901    -  0.555    -  0.367 |
| rs10380     Codominant     CC     CT  TT  Dominant  CC  CT+TT  Recessive^d^  CC+CT  TT | 175/179  50/49  1/3    175/179  53/50    225/228  3/1 | 1.00  1.08(0.69–1.70)  2.98(0.33–29.93)    1.00  1.08(0.69–1.70)    1.00  2.98(0.40–27.93) | -  0.799  0.345    -  0.729    -  0.298 | 1.00  1.26(0.70–2.23)  11.67(0.47–376.93)    1.00  1.24(0.69–2.32)    1.00  9.80(0.44–297.92) | -  0.527  0.154    -  0.470    -  0.180 |

^a^Model I, analysis was performed using crude conditional logistic regression.

^b^Model II, analyses were performed using conditional logistic regression analysis adjusted for the following variables (reference categories are underlined): sex (women, men) age (50–59 y old, 60–69 y old), BMI (normal weight, overweight/obesity), physical exercise (< 15 min/day of cycling/sports, ≥15 min/day), smoking status (never, past/currently: smoker: ≤ 15 cigarettes/day, > 15 cigarettes/day), Deprivation Index (quintile 1–3, quintile 4–5), Predictive Risk Modelling (level 1–2, level 3–4), energy intake (kcal/day), dietary fibre (g/day), alcohol intake (g/day), antiplatelet (including non-steroidal anti-inflammatory drugs) and anticoagulants use (dichotomised variable, yes vs no), including SNPs separately; participants with missing data for the confounding variables were included as a separate category for these variables.

^c^A value of *P* < 0.005 was considered significant after the Bonferroni correction (assuming alpha was equal to 0.005, α = 0.05/10). Significant results are highlighted in bold.

^d^Conditional exact logistic regression.

**Supplementary Table S5** Associations between SNP genotypes and colorectal cancer risk, stratified by dietary factors (unadjusted model).

| Genes,  SNP ID (rs),  genotypes^a^,  stratified by  dietary factors | Model I^b^ | | | | | | | | | *P*_interaction_^c^ |
| --- | --- | --- | --- | --- | --- | --- | --- | --- | --- | --- |
|  | T1^d^ | | | T2^d^ | | | T3^d^ | | |  |
|  | Cases / Controls, n | OR(95%CI) | *P^c^* | Cases / Controls, n | OR(95%CI) | *P^c^* | Cases / Controls, n | OR(95%CI) | *P^c^* |  |

| *DNMT3B*(Chr 20) | | | | | | | | | | |
| --- | --- | --- | --- | --- | --- | --- | --- | --- | --- | --- |
| rs2424913 |  |  |  |  |  |  |  |  |  |  |
| Folate |  |  |  |  |  |  |  |  |  | 0.131 |
| CC | 36/36 | 1.00 |  | 49/28 | 1.58(0.84–2.96) | 0.157 | 26/27 | 0.88(0.42–1.81) | 0.718 |  |
| CT + TT | 45/47 | 0.90(0.48–1.69) | 0.740 | 31/43 | 0.67(0.33–1.33) | 0.253 | 42/48 | 0.84(0.45–1.56) | 0.579 |  |
| Vitamin B_2_ |  |  |  |  |  |  |  |  |  | 0.937 |
| CC | 44/36 | 1.00 |  | 42/27 | 1.16(0.60–2.23) | 0.655 | 25/28 | 0.65(0.30–1.39) | 0.266 |  |
| CT + TT | 41/47 | 0.69(0.36–1.32) | 0.261 | 49/44 | 0.88(0.47–1.63) | 0.682 | 28/47 | 0.47(0.24–0.91) | 0.026 |  |
| Vitamin B_6_ |  |  |  |  |  |  |  |  |  | 0.333 |
| CC | 32/33 | 1.00 |  | 52/33 | 1.48(0.78–2.80) | 0.228 | 27/25 | 1.05(0.48–2.30) | 0.910 |  |
| CT + TT | 36/34 | 1.06(0.52–2.16) | 0.877 | 47/52 | 0.90(0.48–1.68) | 0.732 | 35/52 | 0.67(0.34–1.32) | 0.251 |  |
| Vitamin B_12_ |  |  |  |  |  |  |  |  |  | 0.727 |
| CC | 33/32 | 1.00 |  | 36/30 | 1.17(0.60–2.28) | 0.642 | 42/29 | 1.48(0.71–3.06) | 0.292 |  |
| CT + TT | 37/41 | 0.91(0.45–1.84) | 0.793 | 38/50 | 0.75(0.40–1.42) | 0.380 | 43/47 | 0.91(0.46–1.78) | 0.782 |  |
| Met |  |  |  |  |  |  |  |  |  | 0.724 |
| CC | 31/30 | 1.00 |  | 44/31 | 1.40(0.71–2.76) | 0.333 | 36/30 | 1.17(0.58–2.35) | 0.655 |  |
| CT + TT | 41/46 | 0.87(0.45–1.69) | 0.683 | 45/47 | 0.91(0.46–1.78) | 0.778 | 32/45 | 0.72(0.37–1.40) | 0.334 |  |
| Choline |  |  |  |  |  |  |  |  |  | 0.400 |
| CC | 52/30 | 1.00 |  | 37/26 | 0.77(0.38–1.54) | 0.462 | 22/35 | 0.33(0.15–0.70) | **0.004** |  |
| CT + TT | 53/45 | 0.62(0.32–1.18) | 0.144 | 38/51 | 0.43(0.22–0.82) | 0.011 | 27/42 | 0.34(0.17–0.69) | **0.003** |  |
| Betaine |  |  |  |  |  |  |  |  |  | 0.059 |
| CC | 73/26 | 1.00 |  | 23/27 | 0.30(0.14–0.67) | **0.003** | 15/38 | 0.13(0.06–0.29) | **< 0.001** |  |
| CT + TT | 77/51 | 0.55(0.30–1.00) | 0.051 | 18/48 | 0.13(0.06–0.27) | **< 0.001** | 23/39 | 0.27(0.14–0.54) | **< 0.001** |  |
| rs406193 |  |  |  |  |  |  |  |  |  |  |
| Folate |  |  |  |  |  |  |  |  |  | 0.355 |
| CC | 59/57 | 1.00 |  | 58/49 | 1.12(0.65–1.92) | 0.689 | 46/57 | 0.78(0.46–1.33) | 0.369 |  |
| CT + TT | 21/26 | 0.77(0.38–1.56) | 0.472 | 21/22 | 0.93(0.46–1.86) | 0.835 | 22/18 | 1.22(0.58–2.57) | 0.603 |  |
| Vitamin B_2_ |  |  |  |  |  |  |  |  |  | 0.400 |
| CC | 61/56 | 1.00 |  | 64/55 | 1.02(0.61–1.72) | 0.926 | 38/52 | 0.60(0.33–1.09) | 0.095 |  |
| CT + TT | 22/27 | 0.74(0.38–1.43) | 0.368 | 27/16 | 1.53(0.73–3.19) | 0.258 | 15/23 | 0.55(0.25–1.19) | 0.128 |  |
| Vitamin B_6_ |  |  |  |  |  |  |  |  |  | 0.292 |
| CC | 51/45 | 1.00 |  | 67/64 | 0.90(0.53–1.51) | 0.679 | 45/54 | 0.73(0.40–1.32) | 0.295 |  |
| CT + TT | 16/22 | 0.69(0.33–1.43) | 0.314 | 31/21 | 1.21(0.62–2.36) | 0.582 | 17/23 | 0.67(0.32–1.43) | 0.304 |  |
| Vitamin B_12_ |  |  |  |  |  |  |  |  |  | 0.536 |
| CC | 52/51 | 1.00 |  | 57/59 | 0.91(0.54–1.55) | 0.727 | 54/53 | 0.97(0.53–1.75) | 0.908 |  |
| CT + TT | 17/22 | 0.74(0.35–1.57) | 0.430 | 17/21 | 0.73(0.34–1.60) | 0.436 | 30/23 | 1.25(0.63–2.45) | 0.525 |  |
| Met |  |  |  |  |  |  |  |  |  | 0.435 |
| CC | 54/56 | 1.00 |  | 63/51 | 1.29(0.72–2.29) | 0.390 | 46/56 | 0.87(0.49–1.52) | 0.617 |  |
| CT + TT | 17/20 | 0.91(0.43–1.91) | 0.806 | 25/27 | 0.91(0.44–1.85) | 0.788 | 22/19 | 1.18(0.57–2.46) | 0.650 |  |
| Choline |  |  |  |  |  |  |  |  |  | 0.902 |
| CC | 74/51 | 1.00 |  | 55/58 | 0.67(0.40–1.12) | 0.123 | 34/54 | 0.45(0.26–0.78) | **0.004** |  |
| CT + TT | 30/24 | 0.91(0.48–1.74) | 0.782 | 19/19 | 0.73(0.36–1.50) | 0.397 | 15/23 | 0.38(0.16–0.88) | 0.023 |  |
| Betaine^e^ |  |  |  |  |  |  |  |  |  | 0.272 |
| CC | 107/59 | 1.00 |  | 25/50 | 0.30(0.17–0.55) | **< 0.001** | 31/54 | 0.33(0.21–0.66) | **< 0.001** |  |
| CT + TT | 41/18 | 1.18(0.61–2.30) | 0.670 | 16/25 | 0.32(0.16–0.69) | **0.003** | 7/23 | 0.19(0.10–0.57) | **< 0.001** |  |
| *DNMT1* (Chr 19) | | | | | | | | | | |
| rs2228612 |  |  |  |  |  |  |  |  |  |  |
| Folate^e^ |  |  |  |  |  |  |  |  |  | 0.567 |
| TT | 71/77 | 1.00 |  | 69/66 | 1.13(0.70–1.79) | 0.636 | 61/68 | 1.04(0.65–1.63) | 0.903 |  |
| TC + CC | 9/6 | 1.78(0.69–4.98) | 0.340 | 11/4 | 3.70(0.99–13.59) | 0.051 | 7/7 | 1.10(0.35–3.15) | 0.882 |  |
| Vitamin B_2_^e^ |  |  |  |  |  |  |  |  |  | 0.783 |
| TT | 75/77 | 1.00 |  | 79/64 | 1.28(0.83–1.92) | 0.321 | 47/70 | 0.61(0.40–1.09) | 0.086 |  |
| TC + CC | 9/6 | 1.73(0.54–5.16) | 0.333 | 12/7 | 1.54(0.70–4.28) | 0.332 | 6/4 | 1.70(0.45–6.50) | 0.460 |  |
| Vitamin B_6_^e^ |  |  |  |  |  |  |  |  |  | 0.332 |
| TT | 58/63 | 1.00 |  | 89/76 | 1.22(0.80–1.93) | 0.341 | 54/72 | 0.80(0.45–1.36) | 0.380 |  |
| TC + CC | 9/4 | 2.69(0.78–8.68) | 0.142 | 10/9 | 1.14(0.43–3.10) | 0.797 | 8/4 | 2.27(0.59–8.01) | 0.217 |  |
| Vitamin B_12_^e^ |  |  |  |  |  |  |  |  |  | 0.098 |
| TT | 61/66 | 1.00 |  | 62/77 | 0.89(0.54–1.45) | 0.608 | 78/68 | 1.24(0.78–2.01) | 0.417 |  |
| TC + CC | 8/7 | 1.17(0.40–3.33) | 0.801 | 12/3 | 4.02(1.10–14.68) | 0.032 | 7/7 | 1.19(0.38–3.70) | 0.803 |  |
| Met^e^ |  |  |  |  |  |  |  |  |  | 0.293 |
| TT | 59/68 | 1.00 |  | 80/70 | 1.30(0.78–2.12) | 0.351 | 62/73 | 1.01(0.60–1.60) | 0.949 |  |
| TC + CC | 12/7 | 1.90(0.70–5.20) | 0.221 | 9/8 | 1.40(0.52–3.76) | 0.530 | 6/2 | 3.13(0.65–16.23) | 0.160 |  |
| Choline^e^ |  |  |  |  |  |  |  |  |  | 0.280 |
| TT | 93/71 | 1.00 |  | 62/70 | 0.80(0.44–1.12) | 0.156 | 46/70 | 0.45(0.29–0.77) | **0.003** |  |
| TC + CC | 11/3 | 4.80(1.05–23.45) | 0.046 | 13/7 | 1.73(0.61–4.56) | 0.301 | 3/7 | 0.33(0.08–1.30) | 0.105 |  |
| Betaine^e^ |  |  |  |  |  |  |  |  |  | 0.889 |
| TT | 134/72 | 1.00 |  | 36/69 | 0.26(0.14–0.46) | **< 0.001** | 31/70 | 0.30(0.16–0.44) | **< 0.001** |  |
| TC + CC | 15/5 | 1.45(0.44–4.64) | 0.572 | 5/5 | 0.62(0.16–2.61) | 0.497 | 7/7 | 0.63(0.23–1.94) | 0.440 |  |
| *MTHFR* (Chr 1) | | | | | | | | | | |
| rs1476413 |  |  |  |  |  |  |  |  |  |  |
| Folate |  |  |  |  |  |  |  |  |  | 0.924 |
| CC | 46/45 | 1.00 |  | 49/44 | 1.10(0.63–1.92) | 0.740 | 31/35 | 0.91(0.49–1.68) | 0.756 |  |
| CT + TT | 35/38 | 0.92(0.48–1.73) | 0.784 | 31/27 | 1.16(0.58–2.30) | 0.674 | 37/39 | 0.95(0.50–1.80) | 0.874 |  |
| Vitamin B_2_ |  |  |  |  |  |  |  |  |  | 0.482 |
| CC | 51/43 | 1.00 |  | 46/39 | 0.95(0.51–1.79) | 0.883 | 29/42 | 0.54(0.28–1.04) | 0.067 |  |
| CT + TT | 34/40 | 0.75(0.39–1.45) | 0.398 | 45/32 | 1.19(0.64–2.21) | 0.583 | 24/32 | 0.61(0.31–1.22) | 0.165 |  |
| Vitamin B_6_ |  |  |  |  |  |  |  |  |  | 0.745 |
| CC | 40/36 | 1.00 |  | 52/47 | 0.95(0.51–1.77) | 0.878 | 34/41 | 0.69(0.35–1.36) | 0.282 |  |
| CT + TT | 28/31 | 0.83(0.40–1.75) | 0.628 | 47/37 | 1.11(0.59–2.10) | 0.736 | 28/36 | 0.69(0.34–1.41) | 0.311 |  |
| Vitamin B_12_ |  |  |  |  |  |  |  |  |  | 0.570 |
| CC | 38/34 | 1.00 |  | 41/45 | 0.81(0.43–1.56) | 0.536 | 47/45 | 0.92(0.48–1.78) | 0.814 |  |
| CT + TT | 32/39 | 0.74(0.38–1.46) | 0.387 | 33/34 | 0.86(0.44–1.69) | 0.663 | 38/31 | 1.11(0.55–2.21) | 0.778 |  |
| Met |  |  |  |  |  |  |  |  |  | 0.243 |
| CC | 36/45 | 1.00 |  | 50/37 | 1.65(0.89–3.09) | 0.114 | 40/42 | 1.20(0.65–2.21) | 0.565 |  |
| CT + TT | 36/30 | 1.46(0.76–2.81) | 0.254 | 39/41 | 1.11(0.57–2.16) | 0.767 | 28/33 | 1.06(0.51–2.17) | 0.880 |  |
| Choline |  |  |  |  |  |  |  |  |  | **0.002** |
| CC | 66/44 | 1.00 |  | 42/31 | 0.99(0.54–1.80) | 0.966 | 18/49 | 0.29(0.15–0.55) | **< 0.001** |  |
| CT + TT | 39/31 | 0.87(0.46–1.64) | 0.674 | 33/45 | 0.50(0.26–0.95) | 0.036 | 31/28 | 0.71(0.35–1.44) | 0.346 |  |
| Betaine |  |  |  |  |  |  |  |  |  | 0.348 |
| CC | 87/39 | 1.00 |  | 20/41 | 0.22(0.10–0.46) | **< 0.001** | 19/44 | 0.24(0.12–0.47) | **< 0.001** |  |
| CT + TT | 63/38 | 0.85(0.44–1.64) | 0.623 | 21/34 | 0.31(0.16–0.63) | **0.001** | 19/32 | 0.30(0.14–0.62) | **0.001** |  |
| rs1801131 |  |  |  |  |  |  |  |  |  |  |
| Folate |  |  |  |  |  |  |  |  |  | 0.226 |
| TT | 44/43 | 1.00 |  | 48/33 | 1.32(0.73–2.38) | 0.361 | 25/33 | 0.77(0.39–1.51) | 0.442 |  |
| TG + GG | 36/40 | 0.88(0.47–1.64) | 0.689 | 32/37 | 0.83(0.43–1.59) | 0.577 | 43/42 | 1.02(0.55–1.92) | 0.940 |  |
| Vitamin B_2_ |  |  |  |  |  |  |  |  |  | 0.722 |
| TT | 46/40 | 1.00 |  | 45/31 | 1.30(0.69–2.45) | 0.421 | 26/38 | 0.59(0.30–1.16) | 0.124 |  |
| TG + GG | 38/43 | 0.80(0.43–1.50) | 0.489 | 46/40 | 0.99(0.55–1.78) | 0.966 | 27/36 | 0.62(0.32–1.22) | 0.165 |  |
| Vitamin B_6_ |  |  |  |  |  |  |  |  |  | 0.642 |
| TT | 38/33 | 1.00 |  | 49/37 | 1.13(0.60–2.11) | 0.702 | 30/39 | 0.64(0.32–1.29) | 0.213 |  |
| TG + GG | 29/34 | 0.76(0.38–1.55) | 0.455 | 50/48 | 0.89(0.48–1.63) | 0.695 | 32/37 | 0.75(0.38–1.50) | 0.416 |  |
| Vitamin B_12_ |  |  |  |  |  |  |  |  |  | 0.938 |
| TT | 36/35 | 1.00 |  | 38/37 | 1.05(0.53–2.09) | 0.892 | 43/37 | 1.12(0.58–2.17) | 0.726 |  |
| TG + GG | 33/38 | 0.85(0.43–1.67) | 0.633 | 36/43 | 0.82(0.44–1.53) | 0.536 | 42/38 | 1.09(0.56–2.14) | 0.797 |  |
| Met |  |  |  |  |  |  |  |  |  | 0.102 |
| TT | 32/42 | 1.00 |  | 47/34 | 1.77(0.91–3.44) | 0.093 | 38/33 | 1.49(0.75–2.97) | 0.251 |  |
| TG + GG | 39/33 | 1.48(0.75–2.92) | 0.254 | 42/44 | 1.22(0.61–2.43) | 0.578 | 30/42 | 0.96(0.48–1.90) | 0.907 |  |
| Choline |  |  |  |  |  |  |  |  |  | 0.019 |
| TT | 61/40 | 1.00 |  | 39/28 | 0.97(0.52–1.82) | 0.923 | 17/41 | 0.28(0.14–0.57) | **< 0.001** |  |
| TG + GG | 43/34 | 0.78(0.41–1.46) | 0.434 | 36/49 | 0.44(0.23–0.84) | 0.013 | 32/36 | 0.52(0.27–1.02) | 0.058 |  |
| Betaine |  |  |  |  |  |  |  |  |  | 0.401 |
| TT | 78/32 | 1.00 |  | 19/38 | 0.21(0.10–0.45) | **< 0.001** | 20/39 | 0.26(0.13–0.53) | **< 0.001** |  |
| TG + GG | 71/45 | 0.71(0.38–1.34) | 0.294 | 22/36 | 0.27(0.13–0.55) | **< 0.001** | 18/38 | 0.22(0.10–0.46) | **< 0.001** |  |
| rs1801133 |  |  |  |  |  |  |  |  |  |  |
| Folate |  |  |  |  |  |  |  |  |  | 0.488 |
| CC | 33/32 | 1.00 |  | 25/23 | 1.02(0.46–2.26) | 0.955 | 34/28 | 1.25(0.61–2.54) | 0.542 |  |
| CT + TT | 48/51 | 0.94(0.49–1.80) | 0.856 | 54/48 | 1.10(0.57–2.12) | 0.771 | 34/47 | 0.72(0.37–1.40) | 0.330 |  |
| Vitamin B_2_ |  |  |  |  |  |  |  |  |  | 0.696 |
| CC | 36/29 | 1.00 |  | 39/32 | 0.93(0.46–1.87) | 0.842 | 17/22 | 0.53(0.22–1.24) | 0.143 |  |
| CT + TT | 49/54 | 0.69(0.36–1.34) | 0.275 | 51/39 | 1.02(0.55–1.90) | 0.951 | 36/53 | 0.52(0.27–1.02) | 0.058 |  |
| Vitamin B_6_ |  |  |  |  |  |  |  |  |  | 0.640 |
| CC | 28/21 | 1.00 |  | 44/38 | 0.82(0.40–1.65) | 0.575 | 20/24 | 0.57(0.24–1.37) | 0.211 |  |
| CT + TT | 40/46 | 0.62(0.29–1.29) | 0.200 | 54/47 | 0.82(0.42–1.61) | 0.562 | 42/53 | 0.57(0.28–1.17) | 0.127 |  |
| Vitamin B_12_ |  |  |  |  |  |  |  |  |  | 0.529 |
| CC | 34/27 | 1.00 |  | 30/34 | 0.69(0.34–1.39) | 0.294 | 28/22 | 1.03(0.49–2.16) | 0.937 |  |
| CT + TT | 36/46 | 0.61(0.31–1.19) | 0.145 | 43/46 | 0.74(0.39–1.39) | 0.345 | 57/54 | 0.90(0.49–1.66) | 0.732 |  |
| Met |  |  |  |  |  |  |  |  |  | 0.259 |
| CC | 33/27 | 1.00 |  | 31/32 | 0.80(0.39–1.66) | 0.556 | 28/24 | 0.98(0.47–2.06) | 0.960 |  |
| CT + TT | 39/49 | 0.67(0.35–1.28) | 0.225 | 57/46 | 1.03(0.53–1.99) | 0.929 | 40/51 | 0.66(0.34–1.27) | 0.213 |  |
| Choline |  |  |  |  |  |  |  |  |  | 0.203 |
| CC | 44/27 | 1.00 |  | 25/31 | 0.49(0.23–1.04) | 0.063 | 23/25 | 0.49(0.21–1.12) | 0.090 |  |
| CT + TT | 61/48 | 0.75(0.39–1.45) | 0.397 | 49/46 | 0.66(0.35–1.24) | 0.198 | 26/52 | 0.31(0.16–0.63) | **0.001** |  |
| Betaine |  |  |  |  |  |  |  |  |  | 0.595 |
| CC | 62/28 | 1.00 |  | 17/25 | 0.33(0.15–0.74) | 0.007 | 13/30 | 0.22(0.10–0.51) | **< 0.001** |  |
| CT + TT | 87/49 | 0.79(0.44–1.43) | 0.442 | 24/50 | 0.20(0.10–0.42) | **< 0.001** | 25/47 | 0.25(0.12–0.49) | **< 0.001** |  |
| *MTHFD1*(Chr 14) | | | | | | | | | | |
| rs8003379 |  |  |  |  |  |  |  |  |  |  |
| Folate |  |  |  |  |  |  |  |  |  | 0.117 |
| AA | 44/53 | 1.00 |  | 48/33 | 1.74(0.94–3.21) | 0.076 | 35/39 | 1.13(0.61–2.08) | 0.691 |  |
| AC + CC | 36/30 | 1.38(0.73–2.63) | 0.323 | 31/38 | 0.97(0.52–1.83) | 0.934 | 33/36 | 1.07(0.60–1.91) | 0.826 |  |
| Vitamin B_2_ |  |  |  |  |  |  |  |  |  | 0.265 |
| AA | 43/50 | 1.00 |  | 56/38 | 1.65(0.93–2.94) | 0.087 | 28/37 | 0.83(0.41–1.66) | 0.595 |  |
| AC + CC | 41/33 | 1.43(0.77–2.64) | 0.253 | 35/33 | 1.23(0.65–2.33) | 0.521 | 24/38 | 0.69(0.36–1.32) | 0.264 |  |
| Vitamin B_6_ |  |  |  |  |  |  |  |  |  | 0.387 |
| AA | 34/39 | 1.00 |  | 56/45 | 1.36(0.74–2.48) | 0.319 | 37/41 | 1.02(0.52–2.01) | 0.958 |  |
| AC + CC | 34/28 | 1.40(0.70–2.82) | 0.341 | 42/40 | 1.16(0.62–2.16) | 0.636 | 24/36 | 0.74(0.36–1.49) | 0.393 |  |
| Vitamin B_12_ |  |  |  |  |  |  |  |  |  | 0.556 |
| AA | 40/44 | 1.00 |  | 36/41 | 0.98(0.52–1.84) | 0.946 | 51/40 | 1.38(0.77–2.45) | 0.278 |  |
| AC + CC | 29/29 | 1.07(0.55–2.07) | 0.840 | 38/39 | 1.05(0.58–1.91) | 0.877 | 33/36 | 1.01(0.53–1.93) | 0.983 |  |
| Met |  |  |  |  |  |  |  |  |  | 0.782 |
| AA | 37/42 | 1.00 |  | 49/40 | 1.37(0.74–2.54) | 0.322 | 41/43 | 1.09(0.58–2.04) | 0.789 |  |
| AC + CC | 34/34 | 1.11(0.57–2.13) | 0.762 | 39/38 | 1.13(0.61–2.10) | 0.705 | 27/32 | 0.94(0.47–1.87) | 0.854 |  |
| Choline |  |  |  |  |  |  |  |  |  | 0.102 |
| AA | 57/43 | 1.00 |  | 37/43 | 0.69(0.38–1.24) | 0.216 | 33/39 | 0.62(0.33–1.17) | 0.141 |  |
| AC + CC | 47/32 | 1.14(0.62–2.07) | 0.678 | 38/34 | 0.87(0.46–1.63) | 0.655 | 15/38 | 0.31(0.15–0.63) | **0.001** |  |
| Betaine^e^ |  |  |  |  |  |  |  |  |  | 0.083 |
| AA | 78/43 | 1.00 |  | 20/39 | 0.30(0.17–0.62) | **0.002** | 29/43 | 0.46(0.25–0.90) | 0.020 |  |
| AC + CC | 70/34 | 1.36(0.74–2.45) | 0.322 | 21/36 | 0.36(0.18–0.74) | **0.004** | 9/34 | 0.19(0.10–0.41) | **< 0.001** |  |
| rs17824591 |  |  |  |  |  |  |  |  |  |  |
| Folate |  |  |  |  |  |  |  |  |  | 0.384 |
| GG | 48/52 | 1.00 |  | 53/39 | 1.47(0.82–2.64) | 0.197 | 39/43 | 1.03(0.55–1.90) | 0.937 |  |
| GA + AA | 33/31 | 1.15(0.59–2.21) | 0.685 | 27/32 | 0.92(0.48–1.77) | 0.799 | 29/32 | 0.98(0.50–1.90) | 0.952 |  |
| Vitamin B_2_ |  |  |  |  |  |  |  |  |  | 0.049 |
| GG | 46/55 | 1.00 |  | 59/38 | 1.74(1.02–2.99) | 0.043 | 35/41 | 0.94(0.49–1.80) | 0.860 |  |
| GA + AA | 39/28 | 1.57(0.83–2.96) | 0.165 | 32/33 | 1.11(0.56–2.19) | 0.759 | 18/34 | 0.58(0.28–1.19) | 0.139 |  |
| Vitamin B_6_ |  |  |  |  |  |  |  |  |  | 0.077 |
| GG | 35/40 | 1.00 |  | 61/53 | 1.29(0.74–2.28) | 0.372 | 44/41 | 1.23(0.63–2.39) | 0.549 |  |
| GA + AA | 33/27 | 1.33(0.67–2.62) | 0.411 | 38/32 | 1.30(0.66–2.53) | 0.446 | 18/36 | 0.50(0.22–1.10) | 0.086 |  |
| Vitamin B_12_ |  |  |  |  |  |  |  |  |  | 0.115 |
| GG | 37/45 | 1.00 |  | 48/40 | 1.41(0.78–2.56) | 0.255 | 55/49 | 1.36(0.76–2.44) | 0.301 |  |
| GA + AA | 33/28 | 1.35(0.69–2.62) | 0.382 | 26/40 | 0.78(0.40–1.52) | 0.464 | 30/27 | 1.38(0.67–2.85) | 0.377 |  |
| Met |  |  |  |  |  |  |  |  |  | 0.020 |
| GG | 41/45 | 1.00 |  | 52/53 | 1.04(0.60–1.80) | 0.894 | 47/36 | 1.47(0.81–2.68) | 0.206 |  |
| GA + AA | 31/31 | 1.04(0.52–2.08) | 0.911 | 37/25 | 1.75(0.85–3.60) | 0.130 | 21/39 | 0.62(0.31–1.22) | 0.163 |  |
| Choline |  |  |  |  |  |  |  |  |  | 0.165 |
| GG | 58/47 | 1.00 |  | 47/41 | 0.97(0.54–1.76) | 0.931 | 35/46 | 0.60(0.33–1.10) | 0.099 |  |
| GA + AA | 47/28 | 1.39(0.74–2.61) | 0.304 | 28/36 | 0.66(0.34–1.26) | 0.205 | 14/31 | 0.38(0.18–0.82) | 0.013 |  |
| Betaine |  |  |  |  |  |  |  |  |  | 0.716 |
| GG | 94/50 | 1.00 |  | 23/43 | 0.28(0.14–0.55) | **< 0.001** | 23/41 | 0.33(0.18–0.64) | **0.001** |  |
| GA + AA | 56/27 | 1.22(0.66–2.24) | 0.522 | 18/32 | 0.32(0.15–0.68) | **0.003** | 15/36 | 0.25(0.12–0.51) | **< 0.001** |  |
| *MTRR* (Chr 5) | | | | | | | | | | |
| rs1801394 |  |  |  |  |  |  |  |  |  |  |
| Folate |  |  |  |  |  |  |  |  |  | 0.568 |
| GG | 25/31 | 1.00 |  | 24/18 | 1.71(0.74–3.99) | 0.211 | 14/17 | 1.07(0.45–2.55) | 0.871 |  |
| GA + AA | 56/52 | 1.39(0.70–2.75) | 0.342 | 56/53 | 1.36(0.70–2.65) | 0.357 | 53/58 | 1.19(0.62–2.31) | 0.599 |  |
| Vitamin B_2_ |  |  |  |  |  |  |  |  |  | 0.156 |
| GG | 23/32 | 1.00 |  | 28/16 | 2.17(1.00–4.72) | 0.051 | 12/18 | 0.79(0.32–1.94) | 0.602 |  |
| GA + AA | 62/51 | 1.66(0.87–3.16) | 0.124 | 63/55 | 1.57(0.83–2.96) | 0.163 | 40/57 | 0.95(0.48–1.87) | 0.875 |  |
| Vitamin B_6_ |  |  |  |  |  |  |  |  |  | 0.125 |
| GG | 16/26 | 1.00 |  | 32/25 | 1.98(0.86–4.57) | 0.107 | 15/15 | 1.51(0.59–3.89) | 0.392 |  |
| GA + AA | 52/41 | 2.14(0.99–4.63) | 0.054 | 67/60 | 1.83(0.90–3.71) | 0.095 | 46/62 | 1.19(0.56–2.53) | 0.656 |  |
| Vitamin B_12_ |  |  |  |  |  |  |  |  |  | 0.872 |
| GG | 20/21 | 1.00 |  | 22/27 | 0.84(0.36–1.97) | 0.688 | 21/18 | 1.22(0.51–2.95) | 0.652 |  |
| GA + AA | 50/52 | 0.99(0.46–2.17) | 0.989 | 52/53 | 1.03(0.49–2.14) | 0.943 | 63/58 | 1.14(0.55–2.37) | 0.717 |  |
| Met |  |  |  |  |  |  |  |  |  | 0.187 |
| GG | 20/16 | 1.00 |  | 21/27 | 0.65(0.28–1.55) | 0.333 | 22/23 | 0.82(0.35–1.94) | 0.651 |  |
| GA + AA | 52/60 | 0.75(0.36–1.56) | 0.438 | 68/51 | 1.13(0.53–2.38) | 0.751 | 45/52 | 0.73(0.35–1.54) | 0.411 |  |
| Choline |  |  |  |  |  |  |  |  |  | 0.877 |
| GG | 29/21 | 1.00 |  | 20/24 | 0.60(0.26–1.37) | 0.223 | 14/21 | 0.46(0.19–1.10) | 0.081 |  |
| GA + AA | 76/54 | 0.98(0.51–1.89) | 0.950 | 54/53 | 0.72(0.37–1.40) | 0.331 | 35/56 | 0.42(0.20–0.88) | 0.021 |  |
| Betaine^e^ |  |  |  |  |  |  |  |  |  | 0.130 |
| GG | 45/25 | 1.00 |  | 13/20 | 0.30(0.12–0.78) | **0.011** | 5/21 | 0.12(0.04–0.40) | **0.001** |  |
| GA + AA | 104/52 | 0.90(0.50–1.70) | 0.775 | 28/55 | 0.26(0.14–0.51) | **0.001** | 33/56 | 0.33<(0.16–0.64) | **0.001** |  |
| rs10380 |  |  |  |  |  |  |  |  |  |  |
| Folate |  |  |  |  |  |  |  |  |  | 0.009 |
| CC | 68/65 | 1.00 |  | 62/49 | 1.22(0.74–2.02) | 0.441 | 45/65 | 0.69(0.42–1.14) | 0.147 |  |
| CT + TT | 13/18 | 0.69(0.31–1.52) | 0.353 | 18/22 | 0.77(0.36–1.66) | 0.505 | 22/10 | 2.32(0.97–5.56) | 0.059 |  |
| VitaminB_2_ |  |  |  |  |  |  |  |  |  | 0.208 |
| CC | 68/63 | 1.00 |  | 68/52 | 1.19(0.75–1.91) | 0.460 | 39/64 | 0.53(0.30–0.91) | 0.022 |  |
| CT + TT | 17/20 | 0.76(0.36–1.60) | 0.471 | 22/19 | 1.12(0.56–2.27) | 0.744 | 14/11 | 1.11(0.45–2.74) | 0.813 |  |
| VitaminB_6_ |  |  |  |  |  |  |  |  |  | 0.717 |
| CC | 51/52 | 1.00 |  | 78/65 | 1.18(0.72–1.91) | 0.513 | 46/62 | 0.73(0.41–1.29) | 0.282 |  |
| CT + TT | 17/15 | 1.14(0.51–2.51) | 0.752 | 21/20 | 1.01(0.48–2.14) | 0.982 | 15/15 | 0.97(0.42–2.20) | 0.933 |  |
| VitaminB_12_ |  |  |  |  |  |  |  |  |  | 0.828 |
| CC | 58/59 | 1.00 |  | 53/60 | 0.89(0.53–1.48) | 0.643 | 64/60 | 1.13(0.67–1.91) | 0.645 |  |
| CT + TT | 12/14 | 0.87(0.38–2.01) | 0.746 | 20/20 | 1.01(0.49–2.09) | 0.979 | 21/16 | 1.35(0.64–2.86) | 0.437 |  |
| Met |  |  |  |  |  |  |  |  |  | 0.516 |
| CC | 54/59 | 1.00 |  | 64/61 | 1.13(0.67–1.89) | 0.647 | 57/59 | 1.05(0.63–1.77) | 0.846 |  |
| CT + TT | 18/17 | 1.10(0.51–2.34) | 0.813 | 24/17 | 1.53(0.73–3.19) | 0.261 | 11/16 | 0.68(0.27–1.74) | 0.420 |  |
| Choline |  |  |  |  |  |  |  |  |  | 0.311 |
| CC | 84/56 | 1.00 |  | 54/61 | 0.60(0.36–1.01) | 0.053 | 37/62 | 0.40(0.23–0.69) | **0.001** |  |
| CT + TT | 20/19 | 0.70(0.31–1.57) | 0.392 | 21/16 | 0.91(0.43–1.92) | 0.802 | 12/15 | 0.52(0.22–1.22) | 0.131 |  |
| Betaine^d^ |  |  |  |  |  |  |  |  |  | 0.712 |
| CC | 111/59 | 1.00 |  | 35/61 | 0.22(0.19–0.45) | **< 0.001** | 29/59 | 0.28(0.19–0.50) | **< 0.001** |  |
| CT + TT | 38/18 | 0.93(0.45–1.73) | 0.805 | 6/14 | 0.27(0.12–0.78) | 0.018 | 9/18 | 0.33(0.13–0.69) | 0.009 |  |

Abbreviations: A, adenine; C, cytosine; Chr, chromosome; CI, confidence interval; G, guanine; Met, methionine; rs, reference single nucleotide polymorphism; OR, odds ratio; SNP, single nucleotide polymorphism; T, thymine.

^a^The most frequent genotype (homozygous) was considered the reference group.

^b^Model I, analysis was performed using crude conditional logistic regression.

^c^A value of *P* < 0.005 was considered significant after the Bonferroni correction. Significant results are highlighted in bold.

^d^Tertiles of nutrient intake: folate (µg/d), for males, T1 ≤ 220.0, T2 220.1–289.0, T3 > 289.0, and females, T1 ≤ 245.0, T2 245.1–300.0, T3: > 300.0; vitamin B_2_(mg/d), T1 ≤ 1.3, T2 1.4–1.7, T3 > 1.70; vitamin B_6_(mg/d), T1 ≤ 1.5, T2 1.6–2.0, T3 > 2.0; vitamin B_12_(µg/d), T1 ≤ 3.9, T2 4.0–5.3, T3 > 5.3; Met (mg/d), for males, T1 ≤ 1324.0, T2 1324.1–1985.0, T3 > 1985.0, and females, T1 ≤ 1564.0, T2 1564.1–2623.0, T3 > 2623.0; choline (mg/d), T1 ≤ 114.0, T2 114.1–190.0, T3 > 190.0; betaine (mg/d), T1 ≤ 119.0, T2 119.1–165.0, T3 > 165.0.

^e^Conditional exact logistic regression.

**Supplementary Table S6** Associations between SNP genotypes and colorectal cancer risk, stratified by dietary factors (adjusted model).

| Genes,  SNP ID (rs),  genotypes^a^,  stratified by  dietary factors | Model II^b^ | | | | | | | | | *P*_interaction_^c^ |
| --- | --- | --- | --- | --- | --- | --- | --- | --- | --- | --- |
|  | T1^d^ | | | T2^d^ | | | T3^d^ | | |  |
|  | Cases/  Controls, n | OR(95%CI) | *P^c^* | Cases / Controls, n | OR(95%CI) | *P^c^* | Cases / Controls, n | OR(95%CI) | *P^c^* |  |
| *DNMT3B* (Chr 20) | | | | | | | | | | |
| rs2424913 |  |  |  |  |  |  |  |  |  |  |
| Folate |  |  |  |  |  |  |  |  |  | 0.698 |
| CC | 36/36 | 1.00 |  | 49/28 | 2.12(0.82–5.47) | 0.125 | 26/27 | 2.02(0.58–7.21) | 0.292 |  |
| CT + TT | 45/47 | 1.44(0.56–3.47) | 0.479 | 31/43 | 0.96(0.35–2.64) | 0.948 | 42/48 | 1.48(0.46–4.71) | 0.518 |  |
| Vitamin B_2_ |  |  |  |  |  |  |  |  |  | 0.889 |
| CC | 44/36 | 1.00 |  | 42/27 | 0.77(0.30–1.88) | 0.525 | 25/28 | 0.54(0.19–1.63) | 0.285 |  |
| CT + TT | 41/47 | 0.77(0.31–1.80) | 0.530 | 49/44 | 0.74(0.29–1.89) | 0.528 | 28/47 | 0.24(0.08–0.80) | 0.015 |  |
| Vitamin B_6_ |  |  |  |  |  |  |  |  |  | 0.582 |
| CC | 32/33 | 1.00 |  | 52/33 | 1.13(0.44–2.90) | 0.805 | 27/25 | 0.99(0.32–2.80) | 0.994 |  |
| CT + TT | 36/34 | 1.32(0.52–3.53) | 0.533 | 47/52 | 0.72(0.27–1.83) | 0.540 | 35/52 | 0.48(0.12–1.30) | 0.181 |  |
| Vitamin B_12_ |  |  |  |  |  |  |  |  |  | 0.701 |
| CC | 33/32 | 1.00 |  | 36/30 | 1.10(0.46–2.67) | 0.870 | 42/29 | 2.00(0.62–6.35) | 0.260 |  |
| CT + TT | 37/41 | 1.12(0.43–2.86) | 0.830 | 38/50 | 0.91(0.34–2.21) | 0.880 | 43/47 | 0.92(0.23–2.55) | 0.790 |  |
| Met |  |  |  |  |  |  |  |  |  | 0.849 |
| CC | 31/30 | 1.00 |  | 44/31 | 0.99(0.38–2.56) | 0.986 | 36/30 | 0.33(0.13–0.98) | 0.048 |  |
| CT + TT | 41/46 | 0.54(0.22–1.29) | 0.193 | 45/47 | 0.73(0.30–1.72) | 0.457 | 32/45 | 0.33(0.14–0.95) | 0.037 |  |
| Choline |  |  |  |  |  |  |  |  |  | 0.333 |
| CC | 52/30 | 1.00 |  | 37/26 | 0.30(0.16–0.92) | 0.035 | 22/35 | 0.26(0.08–0.90) | 0.018 |  |
| CT + TT | 53/45 | 0.40(0.16–0.99) | 0.048 | 38/51 | 0.24(0.12–0.72) | 0.007 | 27/42 | 0.34(0.13–0.92) | 0.023 |  |
| Betaine |  |  |  |  |  |  |  |  |  | 0.055 |
| CC | 73/26 | 1.00 |  | 23/27 | 0.23(0.11–0.82) | 0.015 | 15/38 | 0.12(0.03–0.30) | **< 0.001** |  |
| CT + TT | 77/51 | 0.43(0.19–1.02) | 0.055 | 18/48 | 0.12(0.05–0.44) | **< 0.001** | 23/39 | 0.20(0.09–0.52) | **0.001** |  |
| rs406193 |  |  |  |  |  |  |  |  |  |  |
| Folate |  |  |  |  |  |  |  |  |  | 0.282 |
| CC | 59/57 | 1.00 |  | 58/49 | 1.00(0.44–2.04) | 0.950 | 46/57 | 0.94(0.34–2.50) | 0.920 |  |
| CT + TT | 21/26 | 0.61(0.25–1.58) | 0.290 | 21/22 | 1.20(0.45–3.30) | 0.710 | 22/18 | 2.02(0.60–6.79) | 0.279 |  |
| Vitamin B_2_ |  |  |  |  |  |  |  |  |  | 0.460 |
| CC | 61/56 | 1.00 |  | 64/55 | 0.76(0.36–1.64) | 0.442 | 38/52 | 0.21(0.11–0.71) | 0.010 |  |
| CT + TT | 22/27 | 0.60(0.25–1.44) | 0.240 | 27/16 | 0.98(0.40–2.50) | 0.951 | 15/23 | 0.61(0.25–2.02) | 0.480 |  |
| Vitamin B_6_ |  |  |  |  |  |  |  |  |  | 0.361 |
| CC | 51/45 | 1.00 |  | 67/64 | 0.62(0.23–1.34) | 0.270 | 45/54 | 0.52(0.20–1.10) | 0.099 |  |
| CT + TT | 16/22 | 0.70(0.25–1.82) | 0.444 | 31/21 | 0.88(0.33–2.09) | 0.740 | 17/23 | 0.60(0.12–1.89) | 0.345 |  |
| Vitamin B_12_ |  |  |  |  |  |  |  |  |  | 0.925 |
| CC | 52/51 | 1.00 |  | 57/59 | 0.89(0.43–1.80) | 0.720 | 54/53 | 0.98(0.40–2.51) | 0.989 |  |
| CT + TT | 17/22 | 0.78(0.28–2.10) | 0.610 | 17/21 | 0.87(0.32–2.50) | 0.799 | 30/23 | 1.32(0.44–3.25) | 0.55 |  |
| Met |  |  |  |  |  |  |  |  |  | 0.755 |
| CC | 54/56 | 1.00 |  | 63/51 | 1.27(0.59–2.73) | 0.555 | 46/56 | 0.51(0.22–1.09) | 0.080 |  |
| CT + TT | 17/20 | 1.19(0.42–3.21) | 0.741 | 25/27 | 1.15(0.44–2.83) | 0.860 | 22/19 | 0.62(0.21–1.91) | 0.435 |  |
| Choline |  |  |  |  |  |  |  |  |  | 0.988 |
| CC | 74/51 | 1.00 |  | 55/58 | 0.64(0.32–1.29) | 0.216 | 34/54 | 0.67(0.32–1.40) | 0.288 |  |
| CT + TT | 30/24 | 1.77(0.66–4.60) | 0.215 | 19/19 | 0.62(0.22–1.52) | 0.292 | 15/23 | 0.42(0.11–1.30) | 0.140 |  |
| Betaine^e^ |  |  |  |  |  |  |  |  |  | 0.098 |
| CC | 107/59 | 1.00 |  | 25/50 | 0.45(0.22–0.99) | 0.049 | 31/54 | 0.38(0.15–0.73) | 0.004 |  |
| CT + TT | 41/18 | 2.24(0.81–6.12) | 0.143 | 16/25 | 0.32(0.11–0.90) | 0.032 | 7/23 | 0.12(0.08–0.61) | **0.002** |  |
| *DNMT1*(Chr 19) | | | | | | | | | | |
| rs2228612 |  |  |  |  |  |  |  |  |  |  |
| Folate^e^ |  |  |  |  |  |  |  |  |  | 0.298 |
| TT | 71/77 | 1.00 |  | 69/66 | 1.20(0.62–2.49) | 0.699 | 61/68 | 1.63(0.63–4.19) | 0.380 |  |
| TC + CC | 9/6 | 1.53(0.30–7.85) | 0.623 | 11/4 | 2.45(0.51–12.32) | 0.240 | 7/7 | 1.31(0.39–5.42) | 0.753 |  |
| Vitamin B_2_^e^ |  |  |  |  |  |  |  |  |  | 0.798 |
| TT | 75/77 | 1.00 |  | 79/64 | 1.12(0.63–1.99) | 0.865 | 47/70 | 0.45(0.22–1.02) | 0.053 |  |
| TC + CC | 9/6 | 2.03(0.53–10.00) | 0.319 | 12/7 | 0.90(0.60–1.89) | 0.690 | 6/4 | 0.95(0.75–5.28) | 0.915 |  |
| Vitamin B_6_^e^ |  |  |  |  |  |  |  |  |  | 0.893 |
| TT | 58/63 | 1.00 |  | 89/76 | 0.84(0.45–1.67) | 0.562 | 54/72 | 0.60(0.30–1.40) | 0.198 |  |
| TC + CC | 9/4 | 1.50(0.32–7.45) | 0.636 | 10/9 | 0.83(0.21–3.35) | 0.767 | 8/4 | 0.73(0.22–3.61) | 0.672 |  |
| Vitamin B_12_^e^ |  |  |  |  |  |  |  |  |  | 0.510 |
| TT | 61/66 | 1.00 |  | 62/77 | 0.88(0.46–1.65) | 0.686 | 78/68 | 1.22(0.55–2.81) | 0.683 |  |
| TC + CC | 8/7 | 0.82(0.25–3.10) | 0.850 | 12/3 | 2.18(0.55–9.93) | 0.310 | 7/7 | 0.98(0.26–4.47) | 0.932 |  |
| Met^e^ |  |  |  |  |  |  |  |  |  | 0.199 |
| TT | 59/68 | 1.00 |  | 80/70 | 1.33(0.67–2.59) | 0.482 | 62/73 | 0.43(0.21–0.95) | 0.041 |  |
| TC + CC | 12/7 | 1.20(0.32–4.71) | 0.793 | 9/8 | 0.70(0.22–2.45) | 0.470 | 6/2 | 2.03(0.40–15.01) | 0.442 |  |
| Choline^e^ |  |  |  |  |  |  |  |  |  | 0.282 |
| TT | 93/71 | 1.00 |  | 62/70 | 0.70(0.34–1.21) | 0.133 | 46/70 | 0.62(0.37–1.35) | 0.162 |  |
| TC + CC | 11/3 | 5.81(0.65–53.50) | 0.121 | 13/7 | 0.77(0.22–2.60) | 0.666 | 3/7 | 0.36(0.10–2.20) | 0.301 |  |
| Betaine^e^ |  |  |  |  |  |  |  |  |  | 0.816 |
| TT | 134/72 | 1.00 |  | 36/69 | 0.36(0.17–0.73) | **0.002** | 31/70 | 0.22(0.09–0.52) | **< 0.001** |  |
| TC + CC | 15/5 | 1.52(0.33–7.93) | 0.613 | 5/5 | 0.56(0.07–3.11) | 0.521 | 7/7 | 0.35(0.10–1.35) | 0.132 |  |
| *MTHFR(*Chr 1) | | | | | | | | | | |
| rs1476413 |  |  |  |  |  |  |  |  |  |  |
| Folate |  |  |  |  |  |  |  |  |  | 0.915 |
| CC | 46/45 | 1.00 |  | 49/44 | 1.78(0.82–4.01) | 0.173 | 31/35 | 1.73(0.60–5.00) | 0.332 |  |
| CT + TT | 35/38 | 1.64(0.71–3.90) | 0.260 | 31/27 | 1.33(0.53–3.50) | 0.644 | 37/39 | 2.24(0.68–8.00) | 0.200 |  |
| Vitamin B_2_ |  |  |  |  |  |  |  |  |  | 0.840 |
| CC | 51/43 | 1.00 |  | 46/39 | 0.92(0.38–2.12) | 0.820 | 29/42 | 0.42(0.13–1.05) | 0.064 |  |
| CT + TT | 34/40 | 1.05(0.45–2.60) | 0.901 | 45/32 | 0.99(0.30–2.34) | 0.987 | 24/32 | 0.43(0.13–1.35) | 0.148 |  |
| Vitamin B_6_ |  |  |  |  |  |  |  |  |  | 0.623 |
| CC | 40/36 | 1.00 |  | 52/47 | 0.82(0.29–1.93) | 0.598 | 34/41 | 0.54(0.22–1.51) | 0.232 |  |
| CT + TT | 28/31 | 1.12(0.43–3.00) | 0.780 | 47/37 | 0.86(0.42–2.27) | 0.775 | 28/36 | 0.52(0.12–1.67) | 0.240 |  |
| Vitamin B_12_ |  |  |  |  |  |  |  |  |  | 0.896 |
| CC | 38/34 | 1.00 |  | 41/45 | 0.92(0.37–2.22) | 0.789 | 47/45 | 0.88(0.31–2.55) | 0.816 |  |
| CT + TT | 32/39 | 0.88(0.33–2.12) | 0.740 | 33/34 | 0.92(0.39–2.40) | 0.892 | 38/31 | 1.27(0.42–4.00) | 0.790 |  |
| Met |  |  |  |  |  |  |  |  |  | 0.259 |
| CC | 36/45 | 1.00 |  | 50/37 | 1.35(0.64–3.04) | 0.480 | 40/42 | 0.70(0.32–1.66) | 0.422 |  |
| CT + TT | 36/30 | 1.62(0.67–3.75) | 0.299 | 39/41 | 1.25(0.54–3.12) | 0.621 | 28/33 | 0.46(0.12–1.33) | 0.154 |  |
| Choline |  |  |  |  |  |  |  |  |  | 0.012 |
| CC | 66/44 | 1.00 |  | 42/31 | 0.67(0.37–1.32) | 0.200 | 18/49 | 0.25(0.11–0.66) | **0.003** |  |
| CT + TT | 39/31 | 0.60(0.30–1.44) | 0.248 | 33/45 | 0.33(0.15–0.96) | 0.038 | 31/28 | 1.15(0.42–3.12) | 0.805 |  |
| Betaine |  |  |  |  |  |  |  |  |  | 0.514 |
| CC | 87/39 | 1.00 |  | 20/41 | 0.22(0.09–0.64) | 0.005 | 19/44 | 0.20(0.09–0.52) | **0.001** |  |
| CT + TT | 63/38 | 0.97(0.43–2.21) | 0.932 | 21/34 | 0.42(0.15–1.02) | 0.052 | 19/32 | 0.32(0.12–0.88) | 0.025 |  |
| rs1801131 |  |  |  |  |  |  |  |  |  |  |
| Folate |  |  |  |  |  |  |  |  |  | 0.550 |
| TT | 44/43 | 1.00 |  | 48/33 | 2.11(0.91–4.90) | 0.088 | 25/33 | 1.46(0.46–4.80) | 0.521 |  |
| TG + GG | 36/40 | 1.18(0.53–2.73) | 0.652 | 32/37 | 0.76(0.32–1.99) | 0.535 | 43/42 | 1.72(0.59–5.14) | 0.348 |  |
| Vitamin B_2_ |  |  |  |  |  |  |  |  |  | 0.774 |
| TT | 46/40 | 1.00 |  | 45/31 | 1.21(0.51–2.90) | 0.797 | 26/38 | 0.42(0.16–1.22) | 0.125 |  |
| TG + GG | 38/43 | 0.93(0.41–2.17) | 0.866 | 46/40 | 0.66(0.28–1.72) | 0.410 | 27/36 | 0.40(0.16–1.10) | 0.077 |  |
| Vitamin B_6_ |  |  |  |  |  |  |  |  |  | 0.966 |
| TT | 38/33 | 1.00 |  | 49/37 | 1.05(0.42–2.44) | 0.987 | 30/39 | 0.53(0.18–1.55) | 0.229 |  |
| TG + GG | 29/34 | 0.94(0.40–2.36) | 0.888 | 50/48 | 0.58(0.24–1.47) | 0.249 | 32/37 | 0.46(0.19–1.42) | 0.180 |  |
| Vitamin B_12_ |  |  |  |  |  |  |  |  |  | 0.899 |
| TT | 36/35 | 1.00 |  | 38/37 | 1.09(0.42–2.84) | 0.875 | 43/37 | 1.49(0.49–4.45) | 0.515 |  |
| TG + GG | 33/38 | 0.84(0.34–2.12) | 0.723 | 36/43 | 0.90(0.38–2.15) | 0.775 | 42/38 | 0.93(0.33–2.62) | 0.865 |  |
| Met |  |  |  |  |  |  |  |  |  | 0.025 |
| TT | 32/42 | 1.00 |  | 47/34 | 1.55(0.66–3.67) | 0.344 | 38/33 | 1.04(0.40–2.80) | 0.946 |  |
| TG + GG | 39/33 | 1.33(0.56–3.23) | 0.489 | 42/44 | 1.33(0.55–3.27) | 0.545 | 30/42 | 0.40(0.17–1.09) | 0.055 |  |
| Choline |  |  |  |  |  |  |  |  |  | 0.130 |
| TT | 61/40 | 1.00 |  | 39/28 | 0.66(0.30–1.46) | 0.290 | 17/41 | 0.30(0.12–0.75) | 0.009 |  |
| TG + GG | 43/34 | 0.53(0.23–1.24) | 0.145 | 36/49 | 0.25(0.10–0.65) | **0.004** | 32/36 | 0.55(0.21–1.40) | 0.201 |  |
| Betaine |  |  |  |  |  |  |  |  |  | 0.701 |
| TT | 78/32 | 1.00 |  | 19/38 | 0.24(0.06–0.61) | **0.003** | 20/39 | 0.27(0.09–0.65) | **0.003** |  |
| TG + GG | 71/45 | 0.59(0.23–1.40) | 0.221 | 22/36 | 0.28(0.12–0.74) | 0.008 | 18/38 | 0.12(0.04–0.35) | **< 0.001** |  |
| rs1801133 |  |  |  |  |  |  |  |  |  |  |
| Folate |  |  |  |  |  |  |  |  |  | 0.870 |
| CC | 33/32 | 1.00 |  | 25/23 | 1.11(0.36–3.42) | 0.857 | 34/28 | 1.67(0.51–5.48) | 0.393 |  |
| CT + TT | 48/51 | 0.82(0.34–1.92) | 0.632 | 54/48 | 0.98(0.40–2.42) | 0.947 | 34/47 | 0.90(0.22–2.80) | 0.801 |  |
| Vitamin B_2_ |  |  |  |  |  |  |  |  |  | 0.233 |
| CC | 36/29 | 1.00 |  | 39/32 | 0.98(0.37–2.71) | 0.979 | 17/22 | 0.58(0.18–1.99) | 0.369 |  |
| CT + TT | 49/54 | 0.88(0.36–2.10) | 0.766 | 51/39 | 0.79(0.34–1.85) | 0.569 | 36/53 | 0.34(0.14–0.92) | 0.032 |  |
| Vitamin B_6_ |  |  |  |  |  |  |  |  |  | 0.313 |
| CC | 28/21 | 1.00 |  | 44/38 | 0.85(0.25–2.39) | 0.795 | 20/24 | 0.70(0.20–2.60) | 0.590 |  |
| CT + TT | 40/46 | 0.86(0.34–2.21) | 0.737 | 54/47 | 0.69(0.25–1.70) | 0.380 | 42/53 | 0.41(0.14–1.25) | 0.115 |  |
| Vitamin B_12_ |  |  |  |  |  |  |  |  |  | 0.466 |
| CC | 34/27 | 1.00 |  | 30/34 | 0.56(0.23–1.45) | 0.230 | 28/22 | 1.01(0.31–3.33) | 0.978 |  |
| CT + TT | 36/46 | 0.42(0.15–1.03) | 0.056 | 43/46 | 0.65(0.28–1.52) | 0.313 | 57/54 | 0.72(0.24–1.88) | 0.470 |  |
| Met |  |  |  |  |  |  |  |  |  | 0.379 |
| CC | 33/27 | 1.00 |  | 31/32 | 0.88(0.34–2.26) | 0.786 | 28/24 | 0.49(0.18–1.34) | 0.150 |  |
| CT + TT | 39/49 | 0.60(0.25–1.35) | 0.214 | 57/46 | 0.80(0.33–1.87) | 0.590 | 40/51 | 0.31(0.12–0.80) | 0.015 |  |
| Choline |  |  |  |  |  |  |  |  |  | 0.469 |
| CC | 44/27 | 1.00 |  | 25/31 | 0.66(0.25–1.73) | 0.381 | 23/25 | 0.90(0.31–2.59) | 0.830 |  |
| CT + TT | 61/48 | 1.05(0.45–2.50) | 0.939 | 49/46 | 0.52(0.24–1.16) | 0.112 | 26/52 | 0.42(0.12–1.02) | 0.051 |  |
| Betaine |  |  |  |  |  |  |  |  |  | 0.288 |
| CC | 62/28 | 1.00 |  | 17/25 | 0.33(0.11–0.97) | 0.044 | 13/30 | 0.18(0.05–0.53) | **0.002** |  |
| CT + TT | 87/49 | 0.64(0.27–1.53) | 0.260 | 24/50 | 0.23(0.10–0.60) | **0.002** | 25/47 | 0.15(0.06–0.48) | **< 0.001** |  |
| *MTHFD1*(Chr 14) | | | | | | | | | | |
| rs8003379 |  |  |  |  |  |  |  |  |  |  |
| Folate |  |  |  |  |  |  |  |  |  | 0.056 |
| AA | 44/53 | 1.00 |  | 48/33 | 2.12(0.88–5.01) | 0.109 | 35/39 | 1.57(0.55–4.50) | 0.411 |  |
| AC + CC | 36/30 | 1.45(0.64–3.50) | 0.370 | 31/38 | 0.84(0.34–2.11) | 0.738 | 33/36 | 1.64(0.59–4.57) | 0.360 |  |
| Vitamin B_2_ |  |  |  |  |  |  |  |  |  | 0.080 |
| AA | 43/50 | 1.00 |  | 56/38 | 1.33(0.60–3.09) | 0.504 | 28/37 | 0.54(0.22–1.52) | 0.250 |  |
| AC + CC | 41/33 | 1.52(0.65–3.33) | 0.314 | 35/33 | 0.84(0.36–2.06) | 0.667 | 24/38 | 0.42(0.16–1.15) | 0.101 |  |
| Vitamin B_6_ |  |  |  |  |  |  |  |  |  | 0.027 |
| AA | 34/39 | 1.00 |  | 56/45 | 1.16(0.49–2.83) | 0.712 | 37/41 | 0.78(0.29–2.16) | 0.630 |  |
| AC + CC | 34/28 | 1.85(0.75–4.50) | 0.187 | 42/40 | 0.79(0.31–2.01) | 0.600 | 24/36 | 0.45(0.14–1.32) | 0.145 |  |
| Vitamin B_12_ |  |  |  |  |  |  |  |  |  | 0.245 |
| AA | 40/44 | 1.00 |  | 36/41 | 1.20(0.52–2.94) | 0.690 | 51/40 | 1.90(0.74–4.90) | 0.193 |  |
| AC + CC | 29/29 | 1.73(0.71–4.19) | 0.230 | 38/39 | 1.22(0.52–2.74) | 0.649 | 33/36 | 0.87(0.32–2.52) | 0.771 |  |
| Met |  |  |  |  |  |  |  |  |  | 0.620 |
| AA | 37/42 | 1.00 |  | 49/40 | 1.21(0.55–2.73) | 0.626 | 41/43 | 0.50(0.20–1.22) | 0.125 |  |
| AC + CC | 34/34 | 0.93(0.39–2.21) | 0.860 | 39/38 | 0.91(0.40–2.16) | 0.836 | 27/32 | 0.45(0.17–1.22) | 0.114 |  |
| Choline |  |  |  |  |  |  |  |  |  | 0.199 |
| AA | 57/43 | 1.00 |  | 37/43 | 0.40(0.19–0.95) | 0.030 | 33/39 | 0.56(0.27–1.30) | 0.170 |  |
| AC + CC | 47/32 | 0.74(0.34–1.67) | 0.445 | 38/34 | 0.54(0.54–1.29) | 0.152 | 15/38 | 0.40(0.15–0.95) | 0.035 |  |
| Betaine^e^ |  |  |  |  |  |  |  |  |  | 0.383 |
| AA | 78/43 | 1.00 |  | 20/39 | 0.23(0.12–0.62) | **0.002** | 29/43 | 0.36(0.15–0.82) | 0.016 |  |
| AC + CC | 70/34 | 0.96(0.40–2.12) | 0.802 | 21/36 | 0.45(0.16–1.25) | 0.140 | 9/34 | 0.12(0.04–0.45) | **< 0.001** |  |
| rs17824591 |  |  |  |  |  |  |  |  |  |  |
| Folate |  |  |  |  |  |  |  |  |  | 0.466 |
| GG | 48/52 | 1.00 |  | 53/39 | 1.22(0.55–2.77) | 0.632 | 39/43 | 1.45(0.55–4.00) | 0.549 |  |
| GA + AA | 33/31 | 1.12(0.47–2.55) | 0.789 | 27/32 | 1.34(0.56–3.29) | 0.543 | 29/32 | 1.35(0.33–3.88) | 0.610 |  |
| Vitamin B_2_ |  |  |  |  |  |  |  |  |  | 0.013 |
| GG | 46/55 | 1.00 |  | 59/38 | 1.30(0.60–3.00) | 0.520 | 35/41 | 0.59(0.22–1.66) | 0.307 |  |
| GA + AA | 39/28 | 1.73(0.78–4.01) | 0.188 | 32/33 | 1.03(0.41–2.67) | 0.945 | 18/34 | 0.48(0.18–1.77) | 0.166 |  |
| Vitamin B_6_ |  |  |  |  |  |  |  |  |  | 0.023 |
| GG | 35/40 | 1.00 |  | 61/53 | 0.89(0.38–2.08) | 0.750 | 44/41 | 0.99(0.35–2.75) | 0.975 |  |
| GA + AA | 33/27 | 1.80(0.77–4.42) | 0.207 | 38/32 | 1.27(0.48–3.32) | 0.640 | 18/36 | 0.37(0.12–1.22) | 0.101 |  |
| Vitamin B_12_ |  |  |  |  |  |  |  |  |  | **0.003** |
| GG | 37/45 | 1.00 |  | 48/40 | 2.48(1.04–5.00) | 0.047 | 55/49 | 1.65(0.98–4.45) | 0.051 |  |
| GA + AA | 33/28 | 2.50(1.02–6.00) | 0.049 | 26/40 | 0.74(0.30–1.91) | 0.500 | 30/27 | 2.83(0.88–8.07) | 0.090 |  |
| Met |  |  |  |  |  |  |  |  |  | 0.100 |
| GG | 41/45 | 1.00 |  | 52/53 | 1.03(0.52–2.10) | 0.939 | 47/36 | 0.74(0.32–1.70) | 0.470 |  |
| GA + AA | 31/31 | 1.22(0.50–2.96) | 0.664 | 37/25 | 1.79(0.72–4.38) | 0.233 | 21/39 | 0.42(0.16–1.06) | 0.061 |  |
| Choline |  |  |  |  |  |  |  |  |  | 0.213 |
| GG | 58/47 | 1.00 |  | 47/41 | 0.66(0.32–1.55) | 0.356 | 35/46 | 0.75(0.33–1.67) | 0.465 |  |
| GA + AA | 47/28 | 1.86(0.77–4.59) | 0.180 | 28/36 | 0.68(0.32–1.57) | 0.320 | 14/31 | 0.54(0.21–1.48) | 0.234 |  |
| Betaine |  |  |  |  |  |  |  |  |  | 0.614 |
| GG | 94/50 | 1.00 |  | 23/43 | 0.32(0.12–0.69) | 0.010 | 23/41 | 0.42(0.19–0.92) | 0.030 |  |
| GA + AA | 56/27 | 2.09(0.85–5.22) | 0.116 | 18/32 | 0.64(0.24–1.65) | 0.352 | 15/36 | 0.21(0.13–0.72) | **0.001** |  |
| *MTRR(*Chr 5) |  |  |  |  |  |  |  |  |  |  |
| rs1801394 |  |  |  |  |  |  |  |  |  |  |
| Folate |  |  |  |  |  |  |  |  |  | 0.822 |
| GG | 20/23 | 1.00 |  | 24/18 | 1.32(0.39–4.57) | 0.655 | 14/17 | 1.51(0.40.–5.89) | 0.550 |  |
| GA + AA | 61/60 | 1.34(0.50–3.63) | 0.570 | 56/53 | 1.55(0.54–4.40) | 0.399 | 53/58 | 1.72(0.53–5.62) | 0.380 |  |
| Vitamin B_2_ |  |  |  |  |  |  |  |  |  | 0.612 |
| GG | 25/31 | 1.00 |  | 28/16 | 1.31(0.47–3.93) | 0.636 | 12/18 | 0.46(0.14–1.61) | 0.199 |  |
| GA + AA | 56/52 | 1.51(0.63–3.76) | 0.391 | 63/55 | 1.20(0.48–2.99) | 0.717 | 40/57 | 0.60(0.21–1.70) | 0.319 |  |
| Vitamin B_6_ |  |  |  |  |  |  |  |  |  | 0.544 |
| GG | 23/32 | 1.00 |  | 32/25 | 1.26(0.38–4.27) | 0.716 | 15/15 | 0.99(0.26–3.79) | 0.981 |  |
| GA + AA | 62/51 | 2.15(0.75–6.50) | 0.169 | 67/60 | 1.39(0.47–4.04) | 0.563 | 46/62 | 0.90(0.27–2.)79 | 0.845 |  |
| Vitamin B_12_ |  |  |  |  |  |  |  |  |  | 0.701 |
| GG | 16/26 | 1.00 |  | 22/27 | 0.76(0.22–2.56) | 0.651 | 21/18 | 0.52(0.12–2.25) | 0.380 |  |
| GA + AA | 52/41 | 0.72(0.25–2.33) | 0.604 | 52/53 | 0.77(0.26–2.33) | 0.653 | 63/58 | 1.08(0.32–3.68) | 0.896 |  |
| Met |  |  |  |  |  |  |  |  |  | 0.403 |
| GG | 20/21 | 1.00 |  | 21/27 | 0.46(0.12–1.67) | 0.229 | 22/23 | 0.31(0.08–1.31) | 0.109 |  |
| GA + AA | 50/52 | 0.63(0.22–1.97) | 0.433 | 68/51 | 0.99(0.33–2.94) | 0.919 | 45/52 | 0.34(0.15–1.12) | 0.078 |  |
| Choline |  |  |  |  |  |  |  |  |  | 0.890 |
| GG | 20/16 | 1.00 |  | 20/24 | 0.51(0.18–1.59) | 0.251 | 14/21 | 0.52(0.15–1.81) | 0.305 |  |
| GA + AA | 52/60 | 1.25(0.49–3.21) | 0.664 | 54/53 | 0.67(0.25–1.66) | 0.379 | 35/56 | 0.66(0.25–1.82) | 0.410 |  |
| Betaine^e^ |  |  |  |  |  |  |  |  |  | 0.122 |
| GG | 29/21 | 1.00 |  | 13/20 | 0.43(0.15–1.67) | 0.225 | 5/21 | 0.10(0.08–0.30) | **<0.001** |  |
| GA + AA | 76/54 | 0.99(0.38–2.30) | 0.991 | 28/55 | 0.31(0.13–0.95) | 0.041 | 33/56 | 0.36(0.13–0.96) | 0.042 |  |
| rs10380 |  |  |  |  |  |  |  |  |  |  |
| Folate^e^ |  |  |  |  |  |  |  |  |  | 0.160 |
| CC | 68/65 | 1.00 |  | 62/49 | 1.12(0.60–2.39) | 0.715 | 45/65 | 1.03(0.41–2.89) | 0.796 |  |
| CT + TT | 13/18 | 0.95(0.36–2.43) | 0.859 | 18/22 | 1.35(0.53–3.58) | 0.588 | 22/10 | 2.60(0.80–10.09) | 0.136 |  |
| Vitamin B_2_ |  |  |  |  |  |  |  |  |  | 0.195 |
| CC | 68/63 | 1.00 |  | 68/52 | 0.95(0.44–1.90) | 0.812 | 39/64 | 0.32(0.13–0.79) | 0.010 |  |
| CT + TT | 17/20 | 0.89(0.30–2.15) | 0.712 | 22/19 | 0.90(0.32–2.49) | 0.839 | 14/11 | 0.99(0.37–4.00) | 0.789 |  |
| Vitamin B_6_ |  |  |  |  |  |  |  |  |  | 0.505 |
| CC | 51/52 | 1.00 |  | 78/65 | 0.97(0.44–2.11) | 0.931 | 46/62 | 0.62(0.24–1.60) | 0.323 |  |
| CT + TT | 17/15 | 1.70(0.65–4.77) | 0.321 | 21/20 | 0.85(0.30–2.60) | 0.777 | 15/15 | 0.72(0.20–2.50) | 0.567 |  |
| Vitamin B_12_ |  |  |  |  |  |  |  |  |  | 0.950 |
| CC | 58/59 | 1.00 |  | 53/60 | 0.81(0.38–1.72) | 0.580 | 64/60 | 1.11(0.43–2.84) | 0.829 |  |
| CT + TT | 12/14 | 0.81(0.25–2.49) | 0.732 | 20/20 | 1.12(0.46–2.83) | 0.850 | 21/16 | 1.76(0.57–5.47) | 0.327 |  |
| Met |  |  |  |  |  |  |  |  |  | 0.890 |
| CC | 54/59 | 1.00 |  | 64/61 | 0.93(0.46–1.87) | 0.834 | 57/59 | 0.45(0.21–0.98) | 0.043 |  |
| CT + TT | 18/17 | 0.99(0.34–282) | 0.977 | 24/17 | 1.44(0.55–3.73) | 0.501 | 11/16 | 0.52(0.18–1.90) | 0.333 |  |
| Choline |  |  |  |  |  |  |  |  |  | 0.110 |
| CC | 84/56 | 1.00 |  | 54/61 | 0.48(0.25–0.93) | 0.030 | 37/62 | 0.58(0.29–1.12) | 0.115 |  |
| CT + TT | 20/19 | 1.11(0.31–3.93) | 0.862 | 21/16 | 0.89(0.32–2.29) | 0.799 | 12/15 | 0.60(0.20–1.89) | 0.385 |  |
| Betaine^e^ |  |  |  |  |  |  |  |  |  | 0.304 |
| CC | 111/59 | 1.00 |  | 35/61 | 0.38(0.24–0.83) | 0.019 | 29/59 | 0.29(0.18–0.61) | **0.001** |  |
| CT + TT | 38/18 | 1.04(0.50–2.88) | 0.912 | 6/14 | 0.40(0.19–1.28) | 0.156 | 9/18 | 0.33(0.17–0.91) | 0.033 |  |

Abbreviations: A, adenine; C, cytosine; Chr, chromosome; CI, confidence interval; Met, methionine; G, guanine; rs, reference single nucleotide polymorphism; OR, odds ratio; SNP, single nucleotide polymorphism; T, thymine; T1, first tertile; T2, second tertile; T3, third tertile.

^a^The most frequent genotype (homozygous) was considered the reference group.

^b^Model II, analyses were performed using conditional logistic regression analysis adjusted for the following variables (reference categories are underlined): sex (women, men) age (50–59 y old, 60–69 y old), BMI (normal weight, overweight/obesity), physical exercise (< 15 min/d min/d of cycling/sports, ≥15 min/d), smoking status (never, past/currently: smoker: ≤ 15 cigarettes/d, > 15 cigarettes/d), Deprivation Index (quintile 1–3,quintile 4–5), Predictive Risk Modelling (level 1-2, level 3-4), energy intake (kcal/day), dietary fibre (g/d), alcohol intake (g/d), antiplatelet (including non-steroidal anti-inflammatory drugs) and anticoagulants use (dichotomised variable, yes vs no),including SNPs separately; participants with missing data for the confounding variables were included as a separate category for these variables.

^c^A value of *P* < 0.005 was considered significant after the Bonferroni correction.Significant results are highlighted in bold.

^d^Tertiles of nutrient intake: folate (µg/d), for males, T1 ≤ 220.0, T2 220.1–289.0, T3 > 289.0, and females, T1 ≤ 245.0, T2 245.1–300.0, T3: > 300.0; vitamin B_2_ (mg/d), T1 ≤ 1.3, T2 1.4–1.7, T3 >1.70; vitamin B_6_ (mg/d), T1 ≤ 1.5, T2 1.6–2.0, T3 > 2.0; vitamin B_12_ (µg/d), T1 ≤ 3.9, T2 4.0–5.3, T3 >5.3; Met (mg/d), for males, T1 ≤ 1324.0, T2 1324.1–1985.0, T3 > 1985.0, and females, T1 ≤ 1564.0, T2 1564.1–2623.0, T3 > 2623.0; choline (mg/d), T1 ≤ 114.0, T2 114.1–190.0, T3 > 190.0; betaine (mg/d), T1 ≤ 119.0, T2 119.1–165.0, T3 > 165.0.

^e^Conditional exact logistic regression.

**Supplementary Table S7** Associations between SNP genotypes and distal colorectal cancer risk, stratified by dietary factors (unadjusted model).

| Genes,  SNP ID (rs),  genotypes^a^,  stratified by  dietary factors | Model I^b^ | | | | | | | | | | | | | | | *P*_interaction_^c^ |  |  |
| --- | --- | --- | --- | --- | --- | --- | --- | --- | --- | --- | --- | --- | --- | --- | --- | --- | --- | --- |
|  | T1^d^ | | | | | | | T2^d^ | | | | | T3^d^ | | |  |  |  |
|  | Cases / Controls, n | | OR(95% CI) | | *P^c^* | | | Cases / Controls, n | OR(95% CI) | | *P^c^* | | Cases / Controls, n | OR(95% CI) | *P^c^* |  |  |  |
| *DNMT3B* (Chr 20) | | | | | | | | | | | | | | | | | | |
| rs2424913 | | |  | |  | |  |  | | |  | |  |  |  |  |  | |
| Folate | | |  | |  | |  |  | | |  | |  |  |  |  | 0.160 | |
| CC | | | 27/26 | | 1.00 | |  | 37/23 | | | 1.54(0.81–2.88) | | 0.202 | 16/23 | 1.00(0.42–2.00) | 0.915 |  | |
| CT + TT | | | 35/32 | | 1.03(0.65–1.72) | | 0.862 | 27/37 | | | 0.72(0.37–1.47) | | 0.259 | 36/37 | 0.95(0.44–1.76) | 0.623 |  | |
| Vitamin B_2_ | | |  | |  | |  |  | | |  | |  |  |  |  | 0.730 | |
| CC | | | 31/24 | | 1.00 | |  | 30/23 | | | 1.29(0.64–2.32) | | 0.645 | 19/25 | 0.70(0.45–1.28) | 0.280 |  | |
| CT + TT | | | 32/31 | | 0.77(0.40–1.44) | | 0.267 | 43/36 | | | 0.93(0.53–1.73) | | 0.692 | 23/39 | 0.61(0.24–0.93) | 0.041 |  | |
| Vitamin B_6_ | | |  | |  | |  |  | | |  | |  |  |  |  | 0.293 | |
| CC | | | 23/21 | | 1.00 | |  | 37/28 | | | 1.46(0.81–2.60) | | 0.232 | 20/23 | 1.04(0.57–2.23) | 0.915 |  | |
| CT + TT | | | 28/21 | | 1.08(0.65–2.08) | | 0.767 | 41/44 | | | 0.91(0.60–1.73) | | 0.573 | 29/41 | 0.86(0.40–1.41) | 0.315 |  | |
| Vitamin B_12_ | | |  | |  | |  |  | | |  | |  |  |  |  | 0.602 | |
| CC | | | 21/26 | | 1.00 | |  | 28/23 | | | 1.04(0.69–2.00) | | 0.888 | 31/23 | 1.40(0.73–3.03) | 0.313 |  | |
| CT + TT | | | 28/37 | | 0.98(0.58–1.73) | | 0.687 | 35/37 | | | 0.90(0.44–1.40) | | 0.380 | 35/37 | 1.06(0.60–2.01) | 0.719 |  | |
| Met | | |  | |  | |  |  | | |  | |  |  |  |  | 0.729 | |
| CC | | | 21/28 | | 1.00 | |  | 32/25 | | | 1.30(0.71–2.83) | | 0.277 | 27/19 | 1.21(0.61–2.41) | 0.599 |  | |
| CT + TT | | | 22/43 | | 0.92(0.45–1.82) | | 0.699 | 39/32 | | | 0.97(0.48–1.90) | | 0.835 | 26/31 | 0.90(0.30–1.15) | 0.628 |  | |
| Choline | | |  | |  | |  |  | | |  | |  |  |  |  | 0.202 | |
| CC | | | 37/21 | | 1.00 | |  | 25/20 | | | 0.83(0.45–1.56) | | 0.477 | 18/31 | 0.39(0.22–0.77) | **0.004** |  | |
| CT + TT | | | 47/32 | | 0.65(0.44–1.23) | | 0.193 | 47/38 | | | 0.48(0.29–1.04) | | 0.952 | 20/36 | 0.40(0.26–0.69) | **0.004** |  | |
| Betaine | | |  | |  | |  |  | | |  | |  |  |  |  | 0.009 | |
| CC | | | 50/19 | | 1.00 | |  | 17/21 | | | 0.45(0.26–0.88) | | 0.007 | 13/36 | 0.20(0.08–0.33) | **< 0.001** |  | |
| CT + TT | | | 64/36 | | 0.70(0.36–1.02) | | 0.987 | 15/42 | | | 0.20(0.13–0.54) | | **0.001** | 19/28 | 0.19 (0.07–0.57) | **<0.001** |  | |
| rs406193 | | |  | |  | |  |  | | |  | |  |  |  |  |  | |
| Folate | | |  | |  | |  |  | | |  | |  |  |  |  | 0.302 | |
| CC | | | 47/40 | | 1.00 | | - | 47/41 | | | 1.11(0.64–2.08) | | 0.714 | 38/46 | 0.82(0.50–1.21) | 0.371 |  | |
| CT + TT | | | 14/18 | | 0.92(0.50–1.78) | | 0.523 | 17/19 | | | 1.09(0.55–2.01) | | 0.879 | 14/14 | 1.17(0.63–2.05) | 0.583 |  | |
| Vitamin B_2_^e^ | | |  | |  | |  |  | | |  | |  |  |  |  | 0.399 | |
| CC | | | 47/38 | | 1.00 | | - | 53/44 | | | 1.01(0.79–1.65) | | 0.960 | 32/45 | 0.78(0.30–1.19) | 0.221 |  | |
| CT + TT | | | 15/17 | | 0.79(0.45–1.44) | | 0.323 | 20/15 | | | 1.45(0.82–3.07) | | 0.309 | 10/19 | 0.76(0.34–1.26) | 0.140 |  | |
| Vitamin B_6_^e^ | | |  | |  | |  |  | | |  | |  |  |  |  | 0.182 | |
| CC | | | 40/29 | | 1.00 | | - | 53/53 | | | 0.98(0.48–1.50) | | 0.995 | 39/45 | 0.91(0.43–1.33) | 0.798 |  | |
| CT + TT | | | 10/13 | | 0.72(0.35–1.45) | | 0.319 | 25/13 | | | 1.30(0.70–2.19) | | 0.540 | 10/19 | 0.77(0.44–1.31) | 0.515 |  | |
| Vitamin B_12_^e^ | | |  | |  | |  |  | | |  | |  |  |  |  | 0.543 | |
| CC | | | 41/41 | | 1.00 | | - | 48/45 | | | 1.00(0.60–1.62) | | 0.988 | 43/41 | 1.01(0.48–1.87) | 0.923 |  | |
| CT + TT | | | 8/17 | | 0.75(0.46–1.41) | | 0.520 | 15/15 | | | 0.97(0.55–1.51) | | 0.986 | 22/19 | 1.14(0.61–1.98) | 0.606 |  | |
| Met^e^ | | |  | |  | |  |  | | |  | |  |  |  |  | 0.461 | |
| CC | | | 44/53 | | 1.00 | | - | 52/38 | | | 1.34(0.73–2.35) | | 0.413 | 36/3 | 0.91(0.60–1.62) | 0.715 |  | |
| CT + TT | | | 10/18 | | 0.94(0.44–1.90) | | 0.801 | 18/19 | | | 1.00(0.60–1.82) | | 0.995 | 17/14 | 1.04(0.64–2.40) | 0.635 |  | |
| Choline | | |  | |  | |  |  | | |  | |  |  |  |  | 0.856 | |
| CC | | | 38/61 | | 1.00 | | - | 44/43 | | | 1.03(0.44–1.35) | | 0.828 | 27/46 | 0.63(0.33–0.099) | 0.049 |  | |
| CT + TT | | | 23/15 | | 1.05(0.49–1.92) | | 0.862 | 11/21 | | | 0.93(0.46–1.73) | | 0.470 | 11/21 | 0.41(0.25–1.01) | 0.998 |  | |
| Betaine^e^ | | |  | |  | |  |  | | |  | |  |  |  |  | 0.008 | |
| CC | | | 107/59 | | 1.00 | |  | 25/50 | | | 0.40(0.26–0.82) | | **0.004** | 31/54 | 0.30(0.26–0.61) | **0.001** |  | |
| CT + TT | | | 41/18 | | 1.20(0.60–2.37) | | 0.665 | 16/25 | | | 0.34(0.25–0.83) | | **0.004** | 7/23 | 0.16(0.05–0.35) | **< 0.001** |  | |
| *DNMT1* (Chr 19) | | | | | | | | | | | | | | | | | | |
| rs2228612 | | |  | |  | |  |  | | |  | |  |  |  |  |  | |
| Folate^e^ | | |  | |  | |  |  | | |  | |  |  |  |  | 0.562 | |
| TT | | | 56/53 | | 1.00 | | - | 55/55 | | | 1.00(0.80–1.70) | | 0.955 | 46/54 | 1.02(0.65–1.49) | 0.850 |  | |
| TC + CC | | | 5/5 | | 1.45(0.66–4.77) | | 0.340 | 9/4 | | | 2.83(0.77–12.02) | | 0.069 | 6/6 | 1.09(0.41–3.00) | 0.880 |  | |
| Vitamin B_2_^e^ | | |  | |  | |  |  | | |  | |  |  |  |  | 0.830 | |
| TT | | | 57/50 | | 1.00 | | - | 64/52 | | | 1.33(0.90–1.87) | | 0.293 | 36/60 | 0.65(0.41–1.09) | 0.127 |  | |
| TC + CC | | | 5/5 | | 1.30(0.62–4.4) | | 0.331 | 9/7 | | | 1.52(0.80–4.30) | | 0.267 | 6/3 | 1.60(0.55–7.99) | 0.540 |  | |
| Vitamin B_6_^e^ | | |  | |  | |  |  | | |  | |  |  |  |  | 0.293 | |
| TT | | | 44/38 | | 1.00 | | - | 70/64 | | | 1.35(0.90–2.02) | | 0.502 | 70/60 | 0.90(0.45–1.51) | 0.333 |  | |
| TC + CC | | | 6/4 | | 2.65(0.80–8.89) | | 0.121 | 8/8 | | | 1.17(0.5–3.08 | | 0.791 | 6/3 | 2.22(0.72–7.00) | 0.240 |  | |
| Vitamin B_12_^e^ | | |  | |  | |  |  | | |  | |  |  |  |  | 0.189 | |
| TT | | | 44/51 | | 1.00 | | - | 53/57 | | | 0.80(0.52–1.6) | | 0.691 | 60/54 | 1.(0.77–2.00) | 0.502 |  | |
| TC + CC | | | 4/7 | | 1.15(0.45–3.33) | | 0.760 | 10/3 | | | 3.30(0.99–14.80) | | 0.050 | 6/5 | 1.25(0.66–3.49) | 0.792 |  | |
| Met^e^ | | |  | |  | |  |  | | |  | |  |  |  |  | 0.377 | |
| TT | | | 46/63 | | 1.00 | | - | 63/51 | | | 1.34(0.79–2.00) | | 0.480 | 48/48 | 0.99(0.67–1.51) | 0.991 |  | |
| TC + CC | | | 7/7 | | 1.79 (0.68–4.88) | | 0.332 | 8/6 | | | 1.50(0.73–3.88) | | 0.470 | 5/2 | 2.79(0.3–16.00) | 0.099 |  | |
| Choline^e^ | | |  | |  | |  |  | | |  | |  |  |  |  | 0.287 | |
| TT | | | 74/50 | | 1.00 | | - | 46/52 | | | 0.80(0.50–1.22) | | 0.165 | 37/60 | 0.52(0.34–0.99) | 0.005 |  | |
| TC + CC | | | 9/2 | | 4.00(1.00–23.98) | | 0.005 | 10/6 | | | 1.76(0.64–4.80) | | 0.302 | 1/7 | 0.30(0.09–1.22) | 0.109 |  | |
| Betaine^e^ | | |  | |  | |  |  | | |  | |  |  |  |  | 0.798 | |
| TT | | | 102/50 | | 1.00 | | - | 29/58 | | | 0.39(0.28–0.83) | | **0.004** | 26/54 | 0.280.10–0.41) | **< 0.001** |  | |
| TC + CC | | | 11/5 | | 1.550(0.46–4.70) | | 0.612 | 3/4 | | | 0.63(0.14–2.12) | | 0.470 | 6/6 | 0.93(0.26–1.93) | 0.367 |  | |
| *MTHFR* (Chr 1) | | | | | | | | | | | | | | | | | | |
| rs1476413 | | |  | |  | |  |  | | |  | |  |  |  |  |  | |
| Folate | | |  | |  | |  |  | | |  | |  |  |  |  | 0.793 | |
| CC | | | 33/30 | | 1.00 | | - | 36/38 | | | 1.15(0.83–1.99) | | 0.756 | 21/28 | 0.95(0.53–1.54) | 0.766 |  | |
| CT + TT | | | 29/28 | | 0.93(0.45–1.58) | | 0.783 | 28/22 | | | 1.30(0.65–2.89) | | 0.667 | 31/31 | 0.93(0.55–1.73) | 0.783 |  | |
| Vitamin B_2_ | | |  | |  | |  |  | | |  | |  |  |  |  | 0.512 | |
| CC | | | 35/28 | | 1.00 | |  | 34/31 | | | 1.01(0.55–1.83) | | 0.857 | 21/37 | 0.64(0.29–1.11) | 0.780 |  | |
| CT + TT | | | 28/27 | | 0.80(0.45–1.52) | | 0.440 | 39/28 | | | 1.18(0.62–2.05) | | 0.606 | 21/26 | 0.65(0.33–1.20) | 0.112 |  | |
| Vitamin B_6_ | | |  | |  | |  |  | | |  | |  |  |  |  | 0.601 | |
| CC | | | 28/22 | | 1.00 | |  | 37/39 | | | 0.97(0.56–2.00) | | 0.983 | 25/35 | 0.72(0.35–1.36) | 0.302 |  | |
| CT + TT | | | 28/31 | | 0.93(0.44–1.81) | | 0.596 | 41/32 | | | 1.20(0.60–2.09) | | 0.690 | 24/29 | 0.73(0.35–1.17) | 0.259 |  | |
| Vitamin B12 | | | v | |  | |  |  | | |  | |  |  |  |  | 0.560 | |
| CC | | | 23/26 | | 1.00 | | - | 32/35 | | | 1.00(0.47–1.79) | | 0.912 | 35/35 | 0.97(0.48–1.77) | 0.870 |  | |
| CT + TT | | | 26/32 | | 0.80(0.6–1.37 | | 0.303 | 31/24 | | | 0.99(0.42–1.72) | | 0.909 | 31/25 | 1.20(0.60–2.12) | 0.735 |  | |
| Met | | |  | |  | |  |  | | |  | |  |  |  |  | 0.244 | |
| CC | | | 25/42 | | 1.00 | | - | 38/42 | | | 1.75(0.91–3.13) | | 0.126 | 38/29 | 1.15(0.59–2.27) | 0.530 |  | |
| CT + TT | | | 29/28 | | 1.62(0.60–2.92) | | 0.289 | 33/32 | | | 1.21(0.62–2.20) | | 0.775 | 26/21 | 1.11(0.56-2.21) | 0.793 |  | |
| Choline | | |  | |  | |  |  | | |  | |  |  |  |  | 0.014 | |
| CC | | | 47/29 | | 1.00 | | - | 31/24 | | | 1.04(0.56–1.90) | | 0.883 | 12/43 | 0.35(0.19–0.69) | **0.002** |  | |
| CT + TT | | | 37/24 | | 0.93(0.50–1.8) | | 0.883 | 25/33 | | | 0.67(0.22–1.02) | | 0.053 | 26/24 | 0.76(0.35–1.49) | 0.493 |  | |
| Betaine | | |  | |  | |  |  | | |  | |  |  |  |  | 0.007 | |
| CC | | | 62/28 | | 1.00 | | - | 15/35 | | | 0.37(0.18–0.69) | | **0.002** | 13/33 | 0.23(0.10–0.45) | **< 0.001** |  | |
| CT + TT | | | 52/27 | | 1.01(0.55–1.62) | | 0.901 | 17/28 | | | 0.44(0.30–0.88) | | 0.005 | 19/26 | 0.37(0.19–0.70) | **0.002** |  | |
| rs1801131 | | |  | |  | |  |  | | |  | |  |  |  |  |  | |
| Folate | | |  | |  | |  |  | | |  | |  |  |  |  | 0.162 | |
| TT | | | 33/31 | | 1.00 | | - | 35/28 | | | 1.45(0.83–2.32) | | 0.344 | 18/26 | 0.93(0.45–1.60) | 0.515 |  | |
| TG + GG | | | 28/26 | | 0.92(0.50–1.92) | | 0.678 | 29/27 | | | 0.91(0.50–1.78) | | 0.602 | 34/34 | 1.14(0.65–2.03) | 0.699 |  | |
| Vitamin B_2_ | | |  | |  | |  |  | | |  | |  |  |  |  | 0.606 | |
| TT | | | 32/26 | | 1.00 | | - | 35/25 | | | 1.30(0.73–2.62) | | 0.422 | 19/34 | 0.68(0.36–1.20) | 0.142 |  | |
| TG + GG | | | 30/29 | | 0.90(0.46–1.55) | | 0.512 | 38/34 | | | 1.02(0.75-1.53) | | 0.972 | 23/29 | 0.65(0.43–1.15) | 0.160 |  | |
| Vitamin B_6_ | | |  | |  | |  |  | | |  | |  |  |  |  | 0.733 | |
| TT | | | 27/22 | | 1.00 | | - | 37/30 | | | 1.16(0.65–2.02) | | 0.646 | 22/33 | 0.70(0.36–1.22) | 0.230 |  | |
| TG + GG | | | 23/20 | | 1.08(0.40–1.65) | | 0.459 | 23/43 | | | 0.95(0.55–1.78) | | 0.901 | 27/30 | 0.80(0.40–1.60) | 0.420 |  | |
| Vitamin B_12_ | | |  | |  | |  |  | | |  | |  |  |  |  | 0.767 | |
| TT | | | 24/27 | | 1.00 | | - | 31/29 | | | 1.05(0.52–1.93) | | 0.890 | 31/29 | 1.11(0.62–2.23) | 0.735 |  | |
| TG + GG | | | 24/31 | | 1.03(0.52–1.99) | | 0.803 | 32/31 | | | 1.00(0.49–1.58) | | 0.997 | 35/30 | 1.13(0.74–2.15) | 0.680 |  | |
| Met | | |  | |  | |  |  | | |  | |  |  |  |  | 0.103 | |
| TT | | | 24/39 | | 1.00 | |  | 35/23 | | | 1.76(0.92-3.02) | | 0.105 | 27/23 | 1.29(0.72–2.93) | 0.297 |  | |
| TG + GG | | | 29/31 | | 1.82(0.77–2.96) | | 0.250 | 36/34 | | | 1.10(0.60–2.45) | | 0.551 | 26/27 | 1.03(0.48–2.01) | 0.898 |  | |
| Choline | | |  | |  | |  |  | | |  | |  |  |  |  | 0.051 | |
| TT | | | 45/27 | | 1.00 | | - | 29/22 | | | 1.02(0.55–2.01) | | 0.890 | 12/36 | 0.40(0.27–0.73) | **0.003** |  | |
| TG + GG | | | 38/25 | | 0.83(0.37–1.58) | | 0.450 | 27/36 | | | 0.48(0.22–0.98) | | 0.049 | 26/31 | 0.6(0.23–1.03) | 0.059 |  | |
| Betaine | | |  | |  | |  |  | | |  | |  |  |  |  | 0.280 | |
| TT | | | 57/23 | | 1.00 | | - | 14/32 | | | 0.36(0.22–0.65) | | **0.001** | 15/30 | 0.35(0.23–0.63) | **0.001** |  | |
| TG + GG | | | 56/32 | | 1.11(0.42–1.43) | | 0.320 | 18/30 | | | 0.32(0.13–0.63) | | **0.001** | 17/30 | 0.32(0.17–0.52) | **< 0.001** |  | |
| rs1801133 | | |  | |  | |  |  | | |  | |  |  |  |  |  | |
| Folate | | |  | |  | |  |  | | |  | |  |  |  |  | 0.390 | |
| CC | | | 22/24 | | 1.00 | | - | 27/17 | | | 1.10(0.55–2.38) | | 0.954 | 24/34 | 1.37(0.68–2.48) | 0.517 |  | |
| CT + TT | | | 40/43 | | 1.03(0.63–1.89) | | 0.902 | 44/34 | | | 1.13(0.60–2.40) | | 0.812 | 28/35 | 0.82(0.40–1.43) | 0.340 |  | |
| Vitamin B_2_ | | |  | |  | |  |  | | |  | |  |  |  |  | 0.703 | |
| CC | | | 22/19 | | 1.00 | | - | 30/27 | | | 1.00(0.40–2.01) | | 0.900 | 14/20 | 0.60(0.25–1.28) | 0.140 |  | |
| CT + TT | | | 41/36 | | 0.71(0.52-1.44) | | 0.310 | 43/32 | | | 1.05(0.60–2.03) | | 0.893 | 28/44 | 0.65(0.39–1.10) | 0.110 |  | |
| Vitamin B_6_ | | |  | |  | |  |  | | |  | |  |  |  |  | 0.540 | |
| CC | | | 19/12 | | 1.00 | | - | 31/33 | | | 0.94(0.45–1.77) | | 0.597 | 16/21 | 0.60(0.28–1.41) | 0.218 |  | |
| CT + TT | | | 32/30 | | 0.72(0.32–1.32) | | 0.201 | 47/39 | | | 0.96(0.48–1.70) | | 0.600 | 33/43 | 0.60(0.23–1.21) | 0.137 |  | |
| Vitamin B_12_ | | |  | |  | |  |  | | |  | |  |  |  |  | 0.416 | |
| CC | | | 20/21 | | 1.00 | | - | 25/27 | | | 0.76(0.42–1.50) | | 0.301 | 21/18 | 1.03(0.51–2.14) | 0.905 |  | |
| CT + TT | | | 29/37 | | 0.73(0.35–1.2) | | 0.146 | 38/33 | | | 1.09(0.45–1.46) | | 0.292 | 45/42 | 1.010.54–1.69) | 0.937 |  | |
| Met | | |  | |  | |  |  | | |  | |  |  |  |  | 0.110 | |
| CC | | | 21/25 | | 1.00 | | - | 23/25 | | | 0.99(0.42–1.82) | | 0.716 | 22/16 | 1.05(0.42-1.95) | 0.933 |  | |
| CT + TT | | | 33/32 | | 1.06(0.47–1.23) | | 0.988 | 48/46 | | | 1.04(0.56–2.03) | | 0.930 | 31/34 | 0.82(0.32–1.19) | 0.234 |  | |
| Choline | | |  | |  | |  |  | | |  | |  |  |  |  | 0.193 | |
| CC | | | 32/20 | | 1.00 | | - | 17/25 | | | 0.67(0.32–1.02) | | 0.993 | 17/21 | 0.42(0.24–1.19) | 0.110 |  | |
| CT + TT | | | 52/33 | | 1.28(0.60–1.76) | | 0.410 | 39/33 | | | 0.95(0.36–1.08) | | 0.062 | 21/46 | 0.47(0.20–0.84) | 0.007 |  | |
| Betaine | | |  | |  | |  |  | | |  | |  |  |  |  | 0.015 | |
| CC | | | 42/20 | | 1.00 | | - | 12/20 | | | 0.51(0.27–0.99) | | 0.994 | 12/26 | 0.23(0.16–0.66) | **0.001** |  | |
| CT + TT | | | 72/35 | | 1.60(1.03–2.08) | | 0.048 | 20/43 | | | 0.35(0.23–0.74) | | **0.003** | 20/34 | 0.30(0.15–0.60) | **0.001** |  | |
| *MTHFD1* (Chr 14) | | | | | | | | | | | | | | | | | | |
| rs8003379 | | |  | |  | |  |  | | |  | |  |  |  |  |  | |
| Folate | | |  | |  | |  |  | | |  | |  |  |  |  | 0.156 | |
| AA | | | 33/35 | | 1.00 | | - | 37/28 | | | 1.85(0.90–2.82) | | 0.880 | 26/29 | 1.13(0.66–2.81) | 0.650 |  | |
| AC + CC | | | 28/23 | | 1.24(0.85–1.99) | | 0.332 | 27/32 | | | 1.08(0.49–2.02) | | 0.699 | 26/31 | 1.14(0.60–2.39) | 0.76 |  | |
| Vitamin B_2_ | | |  | |  | |  |  | | |  | |  |  |  |  | 0.235 | |
| AA | | | 33/33 | | 1.00 | | - | 42/28 | | | 1.45(1.08–2.50) | | 0.060 | 21/31 | 0.93(0.40–1.62) | 0.550 |  | |
| AC + CC | | | 30/22 | | 1.23(0.82–2.03) | | 0.262 | 31/31 | | | 1.04(0.90–2.05) | | 0.381 | 20/33 | 0.71(0.50–1.67) | 0.340 |  | |
| Vitamin B_6_ | | |  | |  | |  |  | | |  | |  |  |  |  | 0.337 | |
| AA | | | 27/24 | | 1.00 | | - | 40/35 | | | 1.51(0.75–2.26) | | 0.317 | 29/33 | 1.02(0.64–1.89) | 0.960 |  | |
| AC + CC | | | 24/18 | | 1.55(0.92–2.56) | | 0.333 | 38/37 | | | 1.16(0.62–2.15) | | 0.633 | 19/31 | 0.80(0.31–1.63) | 0.540 |  | |
| Vitamin B_12_ | | |  | |  | |  |  | | |  | |  |  |  |  | 0.645 | |
| AA | | | 27/35 | | 1.00 | | - | 30/27 | | | 1.09(0.79–1.93) | | 0.926 | 39/30 | 1.44(0.82–1.95) | 0.260 |  | |
| AC + CC | | | 22/23 | | 1.17(0.70–2.14) | | 0.767 | 33/33 | | | 1.04(0.80–1.85) | | 0.945 | 26/30 | 1.19(0.65–1.92) | 0.777 |  | |
| Met | | |  | |  | |  |  | | |  | |  |  |  |  | 0.729 | |
| AA | | | 25/37 | | 1.00 | | - | 41/28 | | | 1.36(0.92–2.70) | | 0.310 | 30/27 | 1.06(0.57–1.99) | 0.729 |  | |
| AC + CC | | | 29/34 | | 0.90(0.65–1.97) | | 0.050 | 29/29 | | | 1.10(0.69–1.90) | | 0.789 | 23/23 | 0.82(0.45–1.70) | 0.808 |  | |
| Choline | | |  | |  | |  |  | | |  | |  |  |  |  | 0.220 | |
| AA | | | 45/29 | | 1.00 | | - | 26/29 | | | 0.95(0.47–1.21) | | 0.250 | 35/34 | 0.76(0.47–1.03) | 0.057 |  | |
| AC + CC | | | 39/24 | | 1.15(0.71–2.02) | | 0.696 | 30/24 | | | 0.80(0.55–1.47) | | 0.333 | 12/33 | 0.62(0.35–0.90) | 0.018 |  | |
| Betaine^e^ | | |  | |  | |  |  | | |  | |  |  |  |  | 0.101 | |
| AA | | | 57/29 | | 1.00 | | - | 15/32 | | | 0.43(0.23–0.87) | | 0.007 | 24/31 | 0.40(0.25–0.80) | 0.018 |  | |
| AC + CC | | | 56/26 | | 1.32(0.66–2.49) | | 0.412 | 17/31 | | | 0.41(0.22–0.91) | | 0.006 | 8/29 | 0.13(0.08–0.30) | **<0.001** |  | |
| rs17824591 | | |  | |  | |  |  | | |  | |  |  |  |  |  | |
| Folate | | |  | |  | |  |  | | |  | |  |  |  |  | 0.402 | |
| GG | | | 35/35 | | 1.00 | | - | 42/32 | | | 1.42(0.80–2.60) | | 0.320 | 32/33 | 1.12(0.64**–**1.95**)** | 0.82 |  | |
| GA + AA | | | 27/23 | | 1.17(0.68–2.25) | | 0.580 | 22/28 | | | 0.99(0.60–1.82) | | 0.990 | 20/27 | 0.36(0.22–1.23) | 0.712 |  | |
| Vitamin B_2_ | | |  | |  | |  |  | | |  | |  |  |  |  | 0.101 | |
| GG | | | 36/34 | | 1.00 | | - | 45/30 | | | 1.75(1.08–2.80) | | 0.048 | 28/36 | 0.98(0.60–1.93) | 0.819 |  | |
| GA + AA | | | 27/21 | | 1.39(0.83–2.81) | | 0.177 | 28/29 | | | 1.26(0.67–2.48) | | 0.788 | 14/28 | 0.68(0.31–1.45) | 0.180 |  | |
| Vitamin B_6_ | | |  | |  | |  |  | | |  | |  |  |  |  | 0.051 | |
| GG | | | 25/23 | | 1.00 | | - | 50/43 | | | 1.35(0.76–2.40) | | 0.404 | 34/34 | 1.35(0.72–2.20) | 0.561 |  | |
| GA + AA | | | 26/19 | | 1.50(0.77–2.81) | | 0.420 | 28/29 | | | 1.43(0.60–2.4) | | 0.509 | 15/30 | 0.59(0.20–1.39) | 0.120 |  | |
| Vitamin B_12_ | | |  | |  | |  |  | | |  | |  |  |  |  | 0.060 | |
| GG | | | 25/27 | | 1.00 | | - | 40/27 | | | 1.54(0.70–2.52) | | 0.303 | 44/39 | 1.44(0.69–2.46) | 0.330 |  | |
| GA + AA | | | 24/24 | | 1.12(0.52–2.55) | | 0.390 | 23/33 | | | 0.92(0.46–1.81) | | 0.501 | 22/21 | 1.15(0.61–2.30) | 0.309 |  | |
| Met | | |  | |  | |  |  | | |  | |  |  |  |  | 0.030 | |
| GG | | | 30/40 | | 1.00 | | - | 43/39 | | | 1.09(0.68–1.92) | | 0.874 | 36/21 | 1.50(0.84–2.50) | 0.216 |  | |
| GA + AA | | | 24/31 | | 1.10(0.62–2.05) | | 0.420 | 37/25 | | | 1.42(0.93–3.34) | | 0.226 | 21/39 | 0.77(0.35–1.30) | 0.202 |  | |
| Choline | | |  | |  | |  |  | | |  | |  |  |  |  | 0.098 | |
| GG | | | 48/33 | | 1.00 | | - | 48/33 | | | 1.07(0.65–1.91) | | 0.965 | 26/39 | 0.65(0.31–1.11) | 0.110 |  | |
| GA + AA | | | 36/20 | | 1.50(0.76–2.70) | | 0.523 | 28/36 | | | 0.88(0.35–1.24) | | 0.212 | 12/28 | 0.40(0.21–0.98) | 0.047 |  | |
| Betaine | | |  | |  | |  |  | | |  | |  |  |  |  | 0.005 | |
| GG | | | 74/36 | | 1.00 | | - | 16/34 | | | 0.43(0.23–0.70) | | **0.002** | 19/30 | 0.35(0.22–0.70) | **0.002** |  | |
| GA + AA | | | 40/19 | | 1.36(0.71–2.23) | | 0.550 | 16/29 | | | 0.51(0.25–0.90) | | 0.005 | 13/30 | 0.30(0.21–0.72) | **0.002** |  | |
| *MTRR* (Chr 5) | | | | | | | | | | | | | | | | | | |
| rs1801394 | | |  | |  | |  |  | | |  | |  |  |  |  |  | |
| Folate^e^ | | |  | |  | |  |  | | |  | |  |  |  |  | 0.527 | |
| GG | | | 19/18 | | 1.00 | | - | 20/14 | | | 1.72(0.71–2.88) | | 0.230 | 9/15 | 0.91(0.33–2.70) | 0.788 |  | |
| GA + AA | | | 43/40 | | 1.43(0.69–2.62) | | 0.337 | 44/46 | | | 1.00(0.30–2.22) | | 0.981 | 42/45 | 1.40(0.78–2.11) | 0.619 |  | |
| Vitamin B_2_^e^ | | |  | |  | |  |  | | |  | |  |  |  |  | 0.202 | |
| GG | | | 22/19 | | 1.00 | | - | 22/12 | | | 2.10(1.11–4.20) | | 0.089 | 7/16 | 0.82(0.35–1.90) | 0.555 |  | |
| GA + AA | | | 44/36 | | 1.77(0.91–2.98) | | 0.150 | 51/47 | | | 1.73(0.88–2.81) | | 0.220 | 34/48 | 0.92(0.53–1.84) | 0.755 |  | |
| Vitamin B_6_^e^ | | |  | |  | |  |  | | |  | |  |  |  |  | 0.108 | |
| GG | | | 12/13 | | 1.00 | | - | 27/22 | | | 1.90(0.81–4.40) | | 0.145 | 9/12 | 0.89(0.43–3.57) | 0.420 |  | |
| GA + AA | | | 39/29 | | 2.20(1.02–3.13) | | 0.055 | 51/50 | | | 1.89(0.9–3.33) | | 0.139 | 39/52 | 0.91(0.6–2.62) | 0.691 |  | |
| Vitamin B_12_ | | |  | |  | |  |  | | |  | |  |  |  |  | 0.662 | |
| GG | | | 13/14 | | 1.00 | | - | 18/20 | | | 0.94(0.46–1.88) | | 0.733 | 17/13 | 1.15(0.48–2.27) | 0.647 |  | |
| GA + AA | | | 36/44 | | 1.09(0.60–1.98) | | 0.929 | 45/47 | | | 1.10(0.51–2.00) | | 0.780 | 48/47 | 1.09(0.51–2.33) | 0.710 |  | |
| Met | | |  | |  | |  |  | | |  | |  |  |  |  | 0.140 | |
| GG | | | 16/13 | | 1.00 | | - | 16/21 | | | 0.75(0.40–1.65) | | 0.342 | 16/13 | 0.88(0.40–2.01) | 0.630 |  | |
| GA + AA | | | 38/58 | | 0.76(0.33–1.65) | | 0.460 | 55/37 | | | 1.21(0.61–2.49) | | 0.703 | 36/37 | 0.89(0.40–1.62) | 0.433 |  | |
| Choline | | |  | |  | |  |  | | |  | |  |  |  |  | 0.660 | |
| GG | | | 24/13 | | 1.00 | | - | 13/17 | | | 0.72(0.25–1.48) | | 0.250 | 11/17 | 0.59(0.25–1.16) | 0.105 |  | |
| GA + AA | | | 60/40 | | 1.09(0.94–1.93) | | 0.825 | 42/41 | | | 0.77(0.30–1.46) | | 0.332 | 27/50 | 0.53(0.26–1.20) | 0.101 |  | |
| Betaine^e^ | | |  | |  | |  |  | | |  | |  |  |  |  | 0.010 | |
| GG | | | 35/18 | | 1.00 | | - | 8/15 | | | 0.34(0.19–0.96) | | 0.033 | 5/14 | 0.21(0.15–0.55) | **0.001** |  | |
| GA + AA | | | 78/37 | | 1.34(0.91–1.57) | | 0.155 | 24/48 | | | 0.34(0.18–0.79) | | 0.012 | 27/46 | 0.31(0.18–0.70) | **0.002** |  | |
| rs10380 | | |  | |  | |  |  | | |  | |  |  |  |  |  | |
| Folate^e^ | | |  | |  | |  |  | | |  | |  |  |  |  | 0.102 | |
| CC | | | 54/44 | | 1.00 | | - | 51/42 | | | 1.26(0.77–2.13) | | 0.409 | 34/53 | 0.72(0.40–1.16) | 0.183 |  | |
| CT + TT | | | 34/14 | | 1.23(0.63–1.93) | | 0.299 | 13/18 | | | 0.82(0.38–1.67) | | 0.512 | 17/7 | 2.35(0.95–5.13) | 0.119 |  | |
| Vitamin B_2_^e^ | | |  | |  | |  |  | | |  | |  |  |  |  | 0.202 | |
| CC | | | 54/42 | | 1.00 | | - | 55/42 | | | 1.30(0.80–2.00) | | 0.588 | 30/55 | 0.56(0.22–1.00) | 0.050 |  | |
| CT + TT | | | 9/13 | | 0.90(0.44–1.75) | | 0.434 | 17/17 | | | 1.22(0.70–2.23) | | 0.679 | 12/9 | 1.19(0.46–2.80) | 0.812 |  | |
| Vitamin B_6_^e^ | | |  | |  | |  |  | | |  | |  |  |  |  | 0.666 | |
| CC | | | 40/33 | | 1.00 | | - | 64/55 | | | 1.23(0.83–2.00) | | 0.451 | 35/51 | 0.79(0.53–1.32) | 0.303 |  | |
| CT + TT | | | 11/9 | | 1.12(0.60–2.60) | | 0.758 | 14/17 | | | 1.12(0.60–2.22) | | 0.940 | 13/13 | 0.90(0.50–2.10) | 0.809 |  | |
| Vitamin B_12_^e^ | | |  | |  | |  |  | | |  | |  |  |  |  | 0.801 | |
| CC | | | 41/48 | | 1.00 | | - | 45/45 | | | 1.00(0.55–1.37) | | 0.967 | 53/46 | 1.13(0.74–1.99) | 0.549 |  | |
| CT + TT | | | 12/14 | | 1.01(0.41–1.99) | | 0.814 | 20/20 | | | 1.01(0.48–2.04) | | 0.977 | 21/16 | 1.23(0.72–2.86) | 0.551 |  | |
| Met^e^ | | |  | |  | |  |  | | |  | |  |  |  |  | 0.533 | |
| CC | | | 54/59 | | 1.00 | |  | 64/61 | | | 1.15(0.72–1.95) | | 0.601 | 57/59 | 1.13(0.65–1.81) | 0.808 |  | |
| CT + TT | | | 8/10 | | 0.95(0.52–2.30) | | 0.829 | 17/15 | | | 1.14(0.77–3.15) | | 0.202 | 13/14 | 0.74(0.21–1.78) | 0.430 |  | |
| Choline^e^ | | |  | |  | |  |  | | |  | |  |  |  |  | 0.350 | |
| CC | | | 42/55 | | 1.00 | | - | 41/45 | | | 1.25(0.89–1.80) | | 0.202 | 12/16 | 0.44(0.25–0.86) | **0.003** |  | |
| CT + TT | | | 12/16 | | 0.98(0.34–1.68) | | 0.583 | 19/11 | | | 1.40(0.65–1.70) | | 0.666 | 7/11 | 0.52(0.19–1.09) | 0.060 |  | |
| Betaine^e^ | | |  | |  | |  |  | | |  | |  |  |  |  | 0.012 | |
| CC | | | 68/39 | | 1.00 | | - | 42/46 | | | 0.36(0.25–0.82) | | **0.003** | 29/54 | 0.16(0.02–0.33) | **<0.001** |  | |
| CT + TT | | | 15/14 | | 0.99(0.45–1.90) | | 0.991 | 14/13 | | | 1.03(0.25–1.02) | | 0.057 | 9/13 | 0.30(0.15–0.71) | **0.001** |  | |

Abbreviations: A, adenine; C, cytosine; Chr, chromosome; CI, confidence interval; G, guanine; Met, methionine; rs, reference single nucleotide polymorphism; OR, odds ratio; SNP, single nucleotide polymorphism; T, thymine.

^a^The most frequent genotype (homozygous) was considered the reference group.

^b^Model I, analysis was performed using crude conditional logistic regression.

^c^A value of *P* < 0.005 was considered significant after the Bonferroni correction. Significant results are highlighted in bold.

^d^Nutrient intake was categorised into tertiles based on the distribution in the control group (only those controls matched with distal colorectal cancer patients), taking into account sex differences when they were significant. Specifically, different cutoff points were applied to estimate tertiles in men and women when significant sex differences were identified. Tertiles of nutrient intake: folate (µg/day), for males, T1 < 219.0, T2 219.0- 288.0, T3 > 288.0, and females, T1 < 245.0, T2 245.0-300.0, T3 > 300.0; vitamin B_2_ (mg/day), T1 < 1.2, T2 1.2-1.6, T3 > 1.6; vitamin B_6_ (mg/day), T1 < 1.4, T2 1.4-2.1, T3 > 2.1; vitamin B_12_ (µg/day), T1 ≤ 3.8, T2 3.9-5.3, T3 > 5.3; Met (mg/day), for males, T1 < 1322.0, T2 1322.0-1986.0, T3 > 1986.0, and females, T1 < 1565.0, T2 1565.0-2623.0, T3 > 2623.0; choline (mg/day), T1 < 114.0, T2 114.0-188.0, T3 > 188.0; betaine (mg/day), T1 < 117.0, T3 117.0-162.0, T3 > 162.0.

^e^Conditional exact logistic regression.

**Supplementary Table S8** Associations between SNP genotypes and distal colorectal cancer risk, stratified by dietary factors (adjusted model).

| Genes,  SNP ID (rs),  genotypes^a^,  stratified by  dietary factors | Model II^b^ | | | | | | | | |  | *P*_interaction_^c^ |
| --- | --- | --- | --- | --- | --- | --- | --- | --- | --- | --- | --- |
|  | T1^d^ | | | | T2^d^ | | | T3^d^ | |  |  |
|  | Cases / Controls, n | | OR(95% CI) | *P^c^* | Cases / Controls, n | OR(95% CI) | *P^c^* | Cases / Controls, n | OR(95% CI) | *P^c^* |  |
| *DNMT3B* (Chr 20) | | | | | | | | | | | |
| rs2424913 | |  |  |  |  |  |  |  |  |  |  |
| Folate | |  |  |  |  |  |  |  |  |  | 0.160 |
| CC | | 27/26 | 1.00 |  | 37/23 | 1.63(0.90–2.97) | 0.183 | 16/23 | 1.02(0.50–2.03) | 0.980 |  |
| CT + TT | | 35/32 | 1.01(0.62–1.76) | 0.919 | 27/37 | 0.72(0.36–1.44) | 0.235 | 36/37 | 0.83(0.41–1.65) | 0.608 |  |
| Vitamin B_2_ | |  |  |  |  |  |  |  |  |  | 0.830 |
| CC | | 31/24 | 1.00 |  | 30/23 | 1.22(0.55–2.30) | 0.579 | 19/25 | 0.78(0.43–1.40) | 0.311 |  |
| CT + TT | | 32/31 | 0.93(0.32–1.42) | 0.237 | 43/36 | 1.02(0.52–1.72) | 0.675 | 23/39 | 0.72(0.34–1.09) | 0.055 |  |
| Vitamin B_6_ | |  |  |  |  |  |  |  |  |  | 0.322 |
| CC | | 23/21 | 1.00 |  | 37/28 | 1.37(0.81–2.62) | 0.190 | 20/23 | 1.09(0.52–2.32) | 0.867 |  |
| CT + TT | | 28/21 | 1.04(0.50–2.11) | 0.810 | 41/44 | 0.93(0.56–1.79) | 0.680 | 29/41 | 0.80(0.37–1.41) | 0.221 |  |
| Vitamin B_12_ | |  |  |  |  |  |  |  |  |  | 0.658 |
| CC | | 21/26 | 1.00 |  | 28/23 | 1.15(0.68–2.13) | 0.629 | 31/23 | 1.36(0.73–3.01) | 0.283 |  |
| CT + TT | | 28/37 | 0.93(0.54–1.95) | 0.698 | 35/37 | 0.76(0.42–1.40) | 0.380 | 35/37 | 0.95(0.63–2.05) | 0.680 |  |
| Met | |  |  |  |  |  |  |  |  |  | 0.668 |
| CC | | 21/28 | 1.00 |  | 32/25 | 1.49(0.77–2.78) | 0.339 | 27/19 | 1.20(0.60–2.52) | 0.539 |  |
| CT + TT | | 22/43 | 0.95(0.56–1.72) | 0.669 | 39/32 | 1.03(0.47–1.92) | 0.820 | 26/31 | 0.85(0.33–1.20) | 0.332 |  |
| Choline | |  |  |  |  |  |  |  |  |  | 0.289 |
| CC | | 37/21 | 1.00 |  | 25/20 | 0.921(0.40–1.60) | 0.338 | 18/31 | 0.32(0.21–0.76) | **0.004** |  |
| CT + TT | | 47/32 | 0.72(0.36–1.28) | 0.160 | 47/38 | 1.01(0.42–1.13) | 0.901 | 20/36 | 0.36(0.19–0.70) | **0.004** |  |
| Betaine | |  |  |  |  |  |  |  |  |  | 0.009 |
| CC | | 50/19 | 1.00 |  | 17/21 | 0.40(0.22–0.71) | **0.004** | 13/36 | 0.18(0.04–0.29) | **< 0.001** |  |
| CT + TT | | 64/36 | 1.02(0.36–1.04) | 0.056 | 15/42 | 0.11(0.02–0.31) | **<0.001** | 19/28 | 0.29 (0.09–0.65) | **0.001** |  |
| rs406193 | |  |  |  |  |  |  |  |  |  |  |
| Folate | |  |  |  |  |  |  |  |  |  | 0.210 |
| CC | | 47/40 | 1.00 | - | 47/41 | 1.17(0.62–2.00) | 0.681 | 38/46 | 0.93(0.46–1.30) | 0.250 |  |
| CT + TT | | 14/18 | 0.83(0.44–1.67) | 0.483 | 17/19 | 0.98(0.56–2.10) | 0.866 | 14/14 | 1.09(0.45–1.96) | 0.461 |  |
| Vitamin B_2_^e^ | |  |  |  |  |  |  |  |  |  | 0.440 |
| CC | | 47/38 | 1.00 | - | 53/44 | 1.07(0.82–1.88) | 0.920 | 32/45 | 0.77(0.38–1.13) | 0.108 |  |
| CT + TT | | 15/17 | 0.80(0.40–1.60) | 0.370 | 20/15 | 1.45(0.75–3.18) | 0.320 | 10/19 | 0.75(0.35–1.24) | 0.140 |  |
| Vitamin B_6_ ^e^ | |  |  |  |  |  |  |  |  |  | 0.180 |
| CC | | 40/29 | 1.00 | - | 53/53 | 1.01(0.55–1.50) | 0.899 | 39/45 | 0.95(0.40–1.30) | 0.666 |  |
| CT + TT | | 10/13 | 0.68(0.30–1.55) | 0.333 | 25/13 | 1.24(0.64–2.29) | 0.601 | 10/19 | 0.80(0.42–1.38) | 0.316 |  |
| Vitamin B_12_^e^ | |  |  |  |  |  |  |  |  |  | 0.601 |
| CC | | 41/41 | 1.00 | - | 48/45 | 0.93(0.56–1.70) | 0.744 | 43/41 | 1.05(0.54–1.82) | 0.920 |  |
| CT + TT | | 8/17 | 0.72(0.42–1.63) | 0.451 | 15/15 | 0.88(0.34–1.62) | 0.573 | 22/19 | 1.20(0.65–2.33) | 0.515 |  |
| Met ^e^ | |  |  |  |  |  |  |  |  |  | 0.444 |
| CC | | 44/53 | 1.00 | - | 52/38 | 1.31(0.74–2.36) | 0.410 | 36/3 | 0.91(0.51–1.62) | 0.599 |  |
| CT + TT | | 10/18 | 0.93(0.45–2.01) | 0.831 | 18/19 | 1.01(0.55–1.88) | 0.930 | 17/14 | 1.11(0.62–2.30) | 0.651 |  |
| Choline | |  |  |  |  |  |  |  |  |  | 0.530 |
| CC | | 38/61 | 1.00 | - | 44/43 | 0.99(0.51–1.36) | 0.882 | 27/46 | 0.63(0.35–0.91) | 0.035 |  |
| CT + TT | | 23/15 | 1.11(0.54–1.79) | 0.779 | 11/21 | 0.79(0.42–1.59) | 0.427 | 11/21 | 0.46(0.22–1.00) | 0.049 |  |
| Betaine^e^ | |  |  |  |  |  |  |  |  |  | 0.299 |
| CC | | 107/59 | 1.00 |  | 25/50 | 0.040(0.29–0.73) | **0.002** | 31/54 | 0.32(0.21–0.69) | **<0.001** |  |
| CT + TT | | 41/18 | 1.23(0.62–2.33) | 0.649 | 16/25 | 0.32(0.20–0.76) | **0.002** | 7/23 | 0.22(0.10–0.55) | **< 0.001** |  |
| *DNMT1* (Chr 19) | | | | | | | | | | | |
| rs2228612 | |  |  |  |  |  |  |  |  |  |  |
| Folate^e^ | |  |  |  |  |  |  |  |  |  | 0.592 |
| TT | | 56/53 | 1.00 | - | 55/55 | 1.03(0.73–1.92) | 0.646 | 46/54 | 1.08(0.70–1.70) | 0.856 |  |
| TC + CC | | 5/5 | 1.40(0.55–4.88) | 0.315 | 9/4 | 3.50(1.01–12.05) | 0.049 | 6/6 | 1.07(0.42–3.09) | 0.805 |  |
| Vitamin B_2_^e^ | |  |  |  |  |  |  |  |  |  | 0.833 |
| TT | | 57/50 | 1.00 | - | 64/52 | 1.31(0.84–1.95) | 0.320 | 36/60 | 0.70(0.43–1.08) | 0.105 |  |
| TC + CC | | 5/5 | 1.33(0.55–4.40) | 0.347 | 9/7 | 1.70(0.70–4.40) | 0.320 | 6/3 | 1.68(0.50–7.73) | 0.559 |  |
| Vitamin B_6_^e^ | |  |  |  |  |  |  |  |  |  | 0.298 |
| TT | | 44/38 | 1.00 | - | 70/64 | 1.39(0.85–2.01) | 0.345 | 70/60 | 0.83(0.46–1.38) | 0.380 |  |
| TC + CC | | 6/4 | 2.70(0.80–8.89) | 0.115 | 8/8 | 1.20(0.35–2.96) | 0.803 | 6/3 | 2.25(0.61–7.93) | 0.213 |  |
| Vitamin B_12_^e^ | |  |  |  |  |  |  |  |  |  | 0.212 |
| TT | | 44/51 | 1.00 | - | 53/57 | 0.82(0.48–1.46) | 0.612 | 60/54 | 1.26(0.82–2.13) | 0.599 |  |
| TC + CC | | 4/7 | 1.17(0.42–3.24) | 0.709 | 10/3 | 3.27(1.13–14.98) | 0.036 | 6/5 | 1.22(0.55–3.69) | 0.786 |  |
| Met^e^ | |  |  |  |  |  |  |  |  |  | 0.376 |
| TT | | 46/63 | 1.00 | - | 63/51 | 1.29(0.77–2.07) | 0.525 | 48/48 | 1.04(0.65–1.70) | 0.979 |  |
| TC + CC | | 7/7 | 2.01(0.72–5.25) | 0.202 | 8/6 | 1.43(0.67–3.92) | 0.490 | 5/2 | 3.02(0.52–16.63) | 0.150 |  |
| Choline^e^ | |  |  |  |  |  |  |  |  |  | 0.297 |
| TT | | 74/50 | 1.00 | - | 46/52 | 0.76(0.50–1.22) | 0.163 | 37/60 | 0.56(0.36–0.93) | 0.005 |  |
| TC + CC | | 9/2 | 3.83(1.05–24.01) | 0.045 | 10/6 | 1.79(0.70–4.71) | 0.308 | 1/7 | 0.36(0.15–1.23) | 0.122 |  |
| Betaine^e^ | |  |  |  |  |  |  |  |  |  | 0.877 |
| TT | | 102/50 | 1.00 | - | 29/58 | 0.32(0.18–0.72) | **0.002** | 26/54 | 0.36(0.16–0.62) | **0.001** |  |
| TC + CC | | 11/5 | 1.45(0.46–4.57) | 0.550 | 3/4 | 0.60(0.19–2.02) | 0.490 | 6/6 | 0.93(0.20–1.90) | 0.280 |  |
| *MTHFR* (Chr 1) | | | | | | | | | | | |
| rs1476413 | |  |  |  |  |  |  |  |  |  |  |
| Folate | |  |  |  |  |  |  |  |  |  | 0.755 |
| CC | | 33/30 | 1.00 | - | 36/38 | 1.14(0.72–1.90) | 0.712 | 21/28 | 0.89(0.49–1.75) | 0.744 |  |
| CT + TT | | 29/28 | 1.00(0.41–1.80) | 0.997 | 28/22 | 1.20(0.63–2.35) | 0.676 | 31/31 | 1.03(0.60–1.81) | 0.790 |  |
| Vitamin B_2_ | |  |  |  |  |  |  |  |  |  | 0.482 |
| CC | | 35/28 | 1.00 |  | 34/31 | 0.99(0.42–1.74) | 0.995 | 21/37 | 0.60(0.33–1.08) | 0.102 |  |
| CT + TT | | 28/27 | 0.83(0.46–1.55) | 0.389 | 39/28 | 1.25(0.66–2.27) | 0.603 | 21/26 | 0.62(0.30–1.35) | 0.182 |  |
| Vitamin B_6_ | |  |  |  |  |  |  |  |  |  | 0.722 |
| CC | | 28/22 | 1.00 |  | 37/39 | 0.99(0.59–1.90) | 0.901 | 25/35 | 0.73(0.31–1.43) | 0.288 |  |
| CT + TT | | 28/31 | 0.92(0.46–1.63) | 0.635 | 41/32 | 1.12(0.62–2.21) | 0.650 | 24/29 | 0.78(0.30–1.33) | 0.280 |  |
| Vitamin B_12_ | |  |  |  |  |  |  |  |  |  | 0.560 |
| CC | | 23/26 | 1.00 | - | 32/35 | 0.89(0.40–1.68) | 0.526 | 35/35 | 0.93(0.35–1.79) | 0.709 |  |
| CT + TT | | 26/32 | 0.83(0.44–1.56) | 0.399 | 31/24 | 1.02(0.55–1.80) | 0.906 | 31/25 | 1.08(0.60–2.26) | 0.796 |  |
| Met | |  |  |  |  |  |  |  |  |  | 0.202 |
| CC | | 25/42 | 1.00 | - | 38/42 | 1.70(0.90–3.02) | 0.123 | 38/29 | 1.27(0.60–2.25) | 0.517 |  |
| CT + TT | | 29/28 | 1.54(0.59–2.83) | 0.289 | 33/32 | 1.02(0.54–2.1) | 0.815 | 26/21 | 1.13(0.60-2.23) | 0.923 |  |
| Choline | |  |  |  |  |  |  |  |  |  | 0.012 |
| CC | | 47/29 | 1.00 | - | 31/24 | 1.03(0.63–1.83) | 0.977 | 12/43 | 0.38(0.20–0.64) | **0.002** |  |
| CT + TT | | 37/24 | 1.02(0.47–1.7) | 0.670 | 25/33 | 0.64(0.30–1.03) | 0.055 | 26/24 | 0.90(0.44–1.48) | 0.270 |  |
| Betaine | |  |  |  |  |  |  |  |  |  | **0.004** |
| CC | | 62/28 | 1.00 | - | 15/35 | 0.32(0.15–0.63) | **0.002** | 13/33 | 0.22(0.09–0.49) | **< 0.001** |  |
| CT + TT | | 52/27 | 1.05(0.41–1.62) | 0.610 | 17/28 | 0.41(0.23–0.98) | 0.043 | 19/26 | 0.45(0.21–0.82) | 0.010 |  |
| rs1801131 | |  |  |  |  |  |  |  |  |  |  |
| Folate | |  |  |  |  |  |  |  |  |  | 0.230 |
| TT | | 33/31 | 1.00 | - | 35/28 | 1.33(0.83–2.35) | 0.292 | 18/26 | 0.84(0.45–1.63) | 0.487 |  |
| TG + GG | | 28/26 | 1.18(0.48–1.75) | 0.721 | 29/27 | 0.99(0.40–1.73) | 0.497 | 34/34 | 1.05(0.70–2.13) | 0.699 |  |
| Vitamin B_2_ | |  |  |  |  |  |  |  |  |  | 0.718 |
| TT | | 32/26 | 1.00 | - | 35/25 | 1.40(0.78–2.60) | 0.386 | 19/34 | 0.62(0.33–1.20) | 0.130 |  |
| TG + GG | | 30/29 | 1.00(0.50–1.60) | 0.460 | 38/34 | 1.03(0.69–1.63) | 0.979 | 23/29 | 0.67(0.48–1.10) | 0.140 |  |
| Vitamin B_6_ | |  |  |  |  |  |  |  |  |  | 0.616 |
| TT | | 27/22 | 1.00 | - | 37/30 | 1.15(0.64–2.02) | 0.697 | 22/33 | 0.90(0.23–1.25) | 0.150 |  |
| TG + GG | | 23/20 | 1.04(0.39–1.72) | 0.376 | 23/43 | 0.89(0.48–1.60) | 0.665 | 27/30 | 0.80(0.43–1.68) | 0.393 |  |
| Vitamin B_12_ | |  |  |  |  |  |  |  |  |  | 0.873 |
| TT | | 24/27 | 1.00 | - | 31/29 | 1.03(0.49–1.99) | 0.994 | 31/29 | 1.13(0.60–2.20) | 0.710 |  |
| TG + GG | | 24/31 | 0.92(0.49–1.82) | 0.802 | 32/31 | 1.0(0.51–1.58) | 0.998 | 35/30 | 1.08(0.63–2.11) | 0.788 |  |
| Met | |  |  |  |  |  |  |  |  |  | 0.202 |
| TT | | 24/39 | 1.00 |  | 35/23 | 1.27(0.92-3.02) | 0.108 | 27/23 | 1.37(0.83–2.81) | 0.305 |  |
| TG + GG | | 29/31 | 1.38(0.63–2.98) | 0.252 | 36/34 | 1.1(0.60–2.52) | 0.549 | 26/27 | 1.08(0.51–1.97) | 0.893 |  |
| Choline | |  |  |  |  |  |  |  |  |  | 0.061 |
| TT | | 45/27 | 1.00 | - | 29/22 | 1.05(0.50–1.92) | 0.955 | 12/36 | 0.32(0.19–0.69) | **0.002** |  |
| TG + GG | | 38/25 | 0.80(0.45–1.60) | 0.436 | 27/36 | 0.56(0.24–1.01) | 0.050 | 26/31 | 0.50(0.19–0.92) | 0.020 |  |
| Betaine | |  |  |  |  |  |  |  |  |  | 0.055 |
| TT | | 57/23 | 1.00 | - | 14/32 | 0.36(0.19–0.64) | **0.001** | 15/30 | 0.39(0.13–0.61) | **< 0.001** |  |
| TG + GG | | 56/32 | 1.13(0.44–1.52) | 0.303 | 18/30 | 0.40(0.19–0.70) | **0.002** | 17/30 | 0.40(0.23–0.70) | **0.002** |  |
| rs1801133 | |  |  |  |  |  |  |  |  |  |  |
| Folate | |  |  |  |  |  |  |  |  |  | 0.291 |
| CC | | 22/24 | 1.00 | - | 27/17 | 1.11(0.54–2.46) | 0.830 | 24/34 | 1.32(0.67–2.48) | 0.502 |  |
| CT + TT | | 40/43 | 0.96(0.54–1.97) | 0.866 | 44/34 | 1.13(0.52–2.50) | 0.732 | 28/35 | 0.80(0.43–1.51) | 0.330 |  |
| Vitamin B_2_ | |  |  |  |  |  |  |  |  |  | 0.652 |
| CC | | 22/19 | 1.00 | - | 30/27 | 0.98(0.40–2.00) | 0.978 | 14/20 | 0.56(0.22–1.33) | 0.140 |  |
| CT + TT | | 41/36 | 0.81(0.49-1.45) | 0.276 | 43/32 | 1.02(0.59–2.0) | 0.868 | 28/44 | 0.63(0.30–1.03) | 0.060 |  |
| Vitamin B_6_ | |  |  |  |  |  |  |  |  |  | 0.517 |
| CC | | 19/12 | 1.00 | - | 31/33 | 0.95(0.42–1.73) | 0.596 | 16/21 | 0.63(0.20–1.35) | 0.311 |  |
| CT + TT | | 32/30 | 0.75(0.31–1.35) | 0.203 | 47/39 | 1.01(0.49–1.77) | 0.588 | 33/43 | 0.50(0.29–1.15) | 0.130 |  |
| Vitamin B_12_ | |  |  |  |  |  |  |  |  |  | 0.530 |
| CC | | 20/21 | 1.00 | - | 25/27 | 0.81(0.40–1.50) | 0.498 | 21/18 | 1.05(0.50–2.14) | 0.855 |  |
| CT + TT | | 29/37 | 0.66(0.35–1.26) | 0.163 | 38/33 | 1.13(0.52–1.49) | 0.315 | 45/42 | 1.01(0.60–1.81) | 0.989 |  |
| Met | |  |  |  |  |  |  |  |  |  | 0.301 |
| CC | | 21/25 | 1.00 | - | 23/25 | 0.93(0.41–1.80) | 0.606 | 22/16 | 1.11(0.43-2.03) | 0.890 |  |
| CT + TT | | 33/32 | 0.91(0.34–1.33) | 0.879 | 48/46 | 1.02(0.55–2.00) | 0.935 | 31/34 | 0.77(0.39–1.32) | 0.241 |  |
| Choline | |  |  |  |  |  |  |  |  |  | 0.199 |
| CC | | 32/20 | 1.00 | - | 17/25 | 0.63(0.30–1.00) | 0.049 | 17/21 | 0.45(0.30–1.20) | 0.082 |  |
| CT + TT | | 52/33 | 1.27(0.60–1.67) | 0.431 | 39/33 | 1.00(0.32–1.26) | 0.997 | 21/46 | 0.40(0.21–0.90) | **0.004** |  |
| Betaine | |  |  |  |  |  |  |  |  |  | 0.047 |
| CC | | 42/20 | 1.00 | - | 12/20 | 0.41(0.14–0.98) | 0.047 | 12/26 | 0.15(0.09–0.55) | **<0.001** |  |
| CT + TT | | 72/35 | 1.52(0.90–1.95) | 0.375 | 20/43 | 0.32(0.19–0.70) | **0.003** | 20/34 | 0.25(0.13–0.51) | **<0.001** |  |
| *MTHFD1* (Chr 14) | | | | | | | | | | | |
| rs8003379 | |  |  |  |  |  |  |  |  |  |  |
| Folate | |  |  |  |  |  |  |  |  |  | 0.115 |
| AA | | 33/35 | 1.00 | - | 37/28 | 1.32(1.01–2.97) | 0.049 | 26/29 | 1.15(0.72–2.91) | 0.698 |  |
| AC + CC | | 28/23 | 1.11(0.70–1.95) | 0.301 | 27/32 | 1.09(0.58–1.96) | 0.890 | 26/31 | 1.03(0.56–2.60) | 0.867 |  |
| Vitamin B_2_ | |  |  |  |  |  |  |  |  |  | 0.226 |
| AA | | 33/33 | 1.00 | - | 42/28 | 1.53(1.05–3.00) | 0.041 | 21/31 | 0.90(0.40–1.58) | 0.590 |  |
| AC + CC | | 30/22 | 1.15(0.70–1.93) | 0.255 | 31/31 | 1.12(0.85–2.56) | 0.520 | 20/33 | 0.75(0.34–1.32) | 0.280 |  |
| Vitamin B_6_ | |  |  |  |  |  |  |  |  |  | 0.367 |
| AA | | 27/24 | 1.00 | - | 40/35 | 1.56(0.80–2.60) | 0.341 | 29/33 | 1.02(0.49–2.03) | 0.960 |  |
| AC + CC | | 24/18 | 1.80(0.93–2.90) | 0.354 | 38/37 | 1.10(0.64–2.01) | 0.630 | 19/31 | 0.74(0.36–1.63) | 0.440 |  |
| Vitamin B_12_ | |  |  |  |  |  |  |  |  |  | 0.525 |
| AA | | 27/35 | 1.00 | - | 30/27 | 1.09(0.58–1.85) | 0.946 | 39/30 | 1.50(0.88–2.26) | 0.320 |  |
| AC + CC | | 22/23 | 1.14(0.62–1.92) | 0.838 | 33/33 | 1.10(0.59–1.80) | 0.920 | 26/30 | 1.10(0.48–1.93) | 0.960 |  |
| Met | |  |  |  |  |  |  |  |  |  | 0.696 |
| AA | | 25/37 | 1.00 | - | 41/28 | 1.35(0.78–2.60) | 0.312 | 30/27 | 1.12(0.44–2.13) | 0.746 |  |
| AC + CC | | 29/34 | 1.02(0.56–2.13) | 0.832 | 29/29 | 1.05(0.68–2.11) | 0.650 | 23/23 | 0.99(0.40–1.80) | 0.545 |  |
| Choline | |  |  |  |  |  |  |  |  |  | 0.177 |
| AA | | 45/29 | 1.00 | - | 26/29 | 0.81(0.40–1.26) | 0.210 | 35/34 | 0.99(0.24–1.23) | 0.170 |  |
| AC + CC | | 39/24 | 1.09(0.62–2.10) | 0.650 | 30/24 | 1.02(0.46–1.72) | 0.633 | 12/33 | 0.45(0.25–0.83) | 0.005 |  |
| Betaine^e^ | |  |  |  |  |  |  |  |  |  | 0.047 |
| AA | | 57/29 | 1.00 | - | 15/32 | 0.30(0.18–0.81) | 0.005 | 24/31 | 0.49(0.28–0.85) | 0.007 |  |
| AC + CC | | 56/26 | 1.43(0.77–2.55) | 0.346 | 17/31 | 0.47(0.20–0.80) | 0.005 | 8/29 | 0.15(0.03–0.32) | **<0.001** |  |
| rs17824591 | |  |  |  |  |  |  |  |  |  |  |
| Folate | |  |  |  |  |  |  |  |  |  | 0.415 |
| GG | | 35/35 | 1.00 | - | 42/32 | 1.53(0.95–2.82) | 0.175 | 32/33 | 0.98(0.55–1.93) | 0.913 |  |
| GA + AA | | 27/23 | 1.18(0.55–2.22) | 0.510 | 22/28 | 0.97(0.45–1.76) | 0.988 | 20/27 | 0.96(0.54–1.90) | 0.880 |  |
| Vitamin B_2_ | |  |  |  |  |  |  |  |  |  | 0.046 |
| GG | | 36/34 | 1.00 | - | 45/30 | 1.26(1.10–2.93) | 0.025 | 28/36 | 0.92(0.50–2.00) | 0.860 |  |
| GA + AA | | 27/21 | 1.22(0.83–2.72) | 0.269 | 28/29 | 1.09(0.60–2.57) | 0.810 | 14/28 | 0.55(0.24–1.30) | 0.140 |  |
| Vitamin B_6_ | |  |  |  |  |  |  |  |  |  | 0.041 |
| GG | | 25/23 | 1.00 | - | 50/43 | 1.19(0.70–2.19) | 0.383 | 34/34 | 1.10(0.59–2.39) | 0.423 |  |
| GA + AA | | 26/19 | 1.31(0.67–2.69) | 0.417 | 28/29 | 1.30(0.72–2.32) | 0.510 | 15/30 | 1.20(0.19–1.30) | 0.089 |  |
| Vitamin B_12_ | |  |  |  |  |  |  |  |  |  | 0.207 |
| GG | | 25/27 | 1.00 | - | 40/27 | 1.65(0.80–2.58) | 0.260 | 44/39 | 1.33(0.76–2.51) | 0.329 |  |
| GA + AA | | 24/24 | 1.54(0.53–2.57) | 0.367 | 23/33 | 0.90(0.50–1.71) | 0.499 | 22/21 | 1.16(0.68–2.91) | 0.409 |  |
| Met | |  |  |  |  |  |  |  |  |  | 0.012 |
| GG | | 30/40 | 1.00 | - | 43/39 | 1.08(0.68–1.93) | 0.915 | 36/21 | 1.39(0.78–2.52) | 0.197 |  |
| GA + AA | | 24/31 | 1.02(0.47–2.07) | 0.799 | 37/25 | 1.57(0.95–3.47) | 0.223 | 21/39 | 0.62(0.20–1.30) | 0.130 |  |
| Choline | |  |  |  |  |  |  |  |  |  | 0.125 |
| GG | | 48/33 | 1.00 | - | 48/33 | 1.08(0.67–1.82) | 0.893 | 26/39 | 0.78(0.46–1.04) | 0.070 |  |
| GA + AA | | 36/20 | 1.50(0.77–2.65) | 0.477 | 28/36 | 0.77(0.35–1.32) | 0.220 | 12/28 | 0.25(0.12–0.90) | 0.030 |  |
| Betaine | |  |  |  |  |  |  |  |  |  | 0.213 |
| GG | | 74/36 | 1.00 | - | 16/34 | 0.40(0.21–0.66) | **0.001** | 19/30 | 0.40(0.25–0.74) | **0.003** |  |
| GA + AA | | 40/19 | 1.22(0.66–2.25) | 0.525 | 16/29 | 0.45(0.21–0.73) | **0.003** | 13/30 | 0.18(0.07–0.61) | **<0.001** |  |
| *MTRR* (Chr 5) | | | | | | | | | | | |
| rs1801394 | |  |  |  |  |  |  |  |  |  |  |
| Folate^e^ | |  |  |  |  |  |  |  |  |  | 0.552 |
| GG | | 19/18 | 1.00 | - | 20/14 | 1.73(0.77–3.05) | 0.220 | 9/15 | 0.90(0.40–2.52) | 0.880 |  |
| GA + AA | | 43/40 | 1.39(0.73–2.26) | 0.343 | 44/46 | 0.89(0.27–2.40) | 0.748 | 42/45 | 1.30(0.77–2.19) | 0.610 |  |
| Vitamin B_2_^e^ | |  |  |  |  |  |  |  |  |  | 0.172 |
| GG | | 22/19 | 1.00 | - | 22/12 | 2.08(1.05–4.63) | 0.055 | 7/16 | 0.83(0.34–1.92) | 0.593 |  |
| GA + AA | | 44/36 | 1.78(0.89–3.09) | 0.132 | 51/47 | 1.60(0.92–2.93) | 0.201 | 34/48 | 0.95(0.60–1.92) | 0.856 |  |
| Vitamin B_6_^e^ | |  |  |  |  |  |  |  |  |  | 0.125 |
| GG | | 12/13 | 1.00 | - | 27/22 | 1.87(0.86–4.60) | 0.102 | 9/12 | 0.92(0.46–3.78) | 0.380 |  |
| GA + AA | | 39/29 | 2.08(0.05–4.54) | 0.046 | 51/50 | 1.90(0.92–3.54) | 0.102 | 39/52 | 0.92(0.65–2.63) | 0.668 |  |
| Vitamin B_12_ | |  |  |  |  |  |  |  |  |  | 0.678 |
| GG | | 13/14 | 1.00 | - | 18/20 | 0.95(0.44–2.08) | 0.670 | 17/13 | 1.24(0.58–2.90) | 0.640 |  |
| GA + AA | | 36/44 | 1.10(0.60–2.20) | 0.979 | 45/47 | 1.05(0.52–2.17) | 0.953 | 48/47 | 1.02(0.55–2.37) | 0.619 |  |
| Met | |  |  |  |  |  |  |  |  |  | 0.210 |
| GG | | 16/13 | 1.00 | - | 16/21 | 0.74(0.31–1.62) | 0.323 | 16/13 | 0.80(0.35–1.91) | 0.647 |  |
| GA + AA | | 38/58 | 0.80(0.42–1.70) | 0.455 | 55/37 | 1.18(0.65–2.34) | 0.740 | 36/37 | 0.90(0.42–1.60) | 0.430 |  |
| Choline | |  |  |  |  |  |  |  |  |  | 0.578 |
| GG | | 24/13 | 1.00 | - | 13/17 | 0.65(0.27–1.52) | 0.230 | 11/17 | 0.60(0.19–1.21) | 0.150 |  |
| GA + AA | | 60/40 | 1.07(0.98–1.92) | 0.982 | 42/41 | 0.80(0.36–1.47) | 0.341 | 27/50 | 0.60(0.20–1.10) | 0.105 |  |
| Betaine^e^ | |  |  |  |  |  |  |  |  |  | **0.004** |
| GG | | 35/18 | 1.00 | - | 8/15 | 0.42(0.13–0.76) | **0.003** | 5/14 | 0.19(0.11–0.52) | **<0.001** |  |
| GA + AA | | 78/37 | 1.45(1.02–1.80) | 0.890 | 24/48 | 0.35(0.14–0.70) | **0.002** | 27/46 | 0.32(0.20–0.70) | **0.002** |  |
| rs10380 | |  |  |  |  |  |  |  |  |  |  |
| Folate^e^ | |  |  |  |  |  |  |  |  |  | 0.020 |
| CC | | 54/44 | 1.00 | - | 51/42 | 1.25(0.78–2.05) | 0.435 | 34/53 | 0.73(0.44–1.16) | 0.160 |  |
| CT + TT | | 34/14 | 1.30(0.45–1.90) | 0.303 | 13/18 | 0.80(0.40–1.70) | 0.489 | 17/7 | 2.25(0.90–5.02) | 0.063 |  |
| Vitamin B_2_^e^ | |  |  |  |  |  |  |  |  |  | 0.190 |
| CC | | 54/42 | 1.00 | - | 55/42 | 1.32(0.78–1.95) | 0.497 | 30/55 | 0.60(0.25–0.90) | 0.019 |  |
| CT + TT | | 9/13 | 0.78(0.40–1.72) | 0.450 | 17/17 | 1.08(0.55–2.20) | 0.710 | 12/9 | 1.09(0.40–2.74) | 0.812 |  |
| Vitamin B_6_^e^ | |  |  |  |  |  |  |  |  |  | 0.689 |
| CC | | 40/33 | 1.00 | - | 64/55 | 1.18(0.83–2.05) | 0.510 | 35/51 | 0.75(0.40–1.32) | 0.301 |  |
| CT + TT | | 11/9 | 1.19(0.65–2.60) | 0.778 | 14/17 | 1.03(0.49–2.17) | 0.989 | 13/13 | 1.01(0.48–2.14) | 0.945 |  |
| Vitamin B_12_ | |  |  |  |  |  |  |  |  |  | 0.789 |
| CC | | 41/48 | 1.00 | - | 45/45 | 1.10(0.57–1.52) | 0.899 | 53/46 | 1.12(0.76–2.00) | 0.645 |  |
| CT + TT | | 12/14 | 1.02(0.38–2.11) | 0.749 | 20/20 | 1.06(0.48–2.15) | 0.890 | 21/16 | 1.40(0.62–2.90) | 0.406 |  |
| Met^e^ | |  |  |  |  |  |  |  |  |  | 0.535 |
| CC | | 54/59 | 1.00 |  | 64/61 | 1.08(0.66–1.89) | 0.644 | 57/59 | 1.06(0.62–1.81) | 0.840 |  |
| CT + TT | | 8/10 | 1.06(0.52–2.35) | 0.809 | 17/15 | 1.37(0.71–3.39) | 0.238 | 13/14 | 0.67(0.28–1.92) | 0.415 |  |
| Choline^e^ | |  |  |  |  |  |  |  |  |  | 0.319 |
| CC | | 42/55 | 1.00 | - | 41/45 | 1.42(0.07–1.82) | 0.211 | 12/16 | 0.46(0.28–0.75) | **0.002** |  |
| CT + TT | | 12/16 | 0.97(0.34–1.75) | 0.602 | 19/11 | 1.30(0.60–1.80) | 0.720 | 7/11 | 0.55(0.22–1.22) | 0.120 |  |
| Betaine^e^ | |  |  |  |  |  |  |  |  |  | 0.825 |
| CC | | 68/39 | 1.00 | - | 42/46 | 0.33(0.20–0.72) | **0.003** | 29/54 | 0.25(0.08–0.36) | **<0.001** |  |
| CT + TT | | 15/14 | 1.02(0.50–1.85) | 0.935 | 14/13 | 1.01(0.30–1.05) | 0.910 | 9/13 | 0.51(0.23–0.85) | 0.007 |  |

Abbreviations: A, adenine; C, cytosine; Chr, chromosome; CI, confidence interval; G, guanine; Met, methionine; rs, reference single nucleotide polymorphism; OR, odds ratio; SNP, single nucleotide polymorphism; T, thymine.

^a^The most frequent genotype (homozygous) was considered the reference group.

^b^Model II, analyses were performed using conditional logistic regression analysis adjusted for the following variables (reference categories are underlined): sex (women, men) age (50–59 y old, 60–69 y old), BMI (normal weight, overweight/obesity), physical exercise (< 15 min/d min/d of cycling/sports, ≥15 min/d), smoking status (never, past/currently: smoker: ≤ 15 cigarettes/d, > 15 cigarettes/d), Deprivation Index (quintile 1–3, quintile 4–5), Predictive Risk Modelling (level 1-2, level 3-4), energy intake (kcal/day), dietary fibre (g/d), alcohol intake (g/d), antiplatelet (including non-steroidal anti-inflammatory drugs) and anticoagulants use (dichotomised variable, yes vs no), including SNPs separately; participants with missing data for the confounding variables were included as a separate category for these variables.

^c^A value of *P* < 0.005 was considered significant after the Bonferroni correction. Significant results are highlighted in bold.

^d^Nutrient intake was categorised into tertiles based on the distribution in the control group (only those controls matched with distal colorectal cancer patients), taking into account sex differences when they were significant. Specifically, different cutoff points were applied to estimate tertiles in men and women when significant sex differences were identified. Tertiles of nutrient intake: folate (µg/day), for males, T1 < 219.0, T2 219.0- 288.0, T3 > 288.0, and females, T1 < 245.0, T2 245.0-300.0, T3: > 300.0; vitamin B_2_ (mg/day), T1 < 1.2, T2 1.2-1.6, T3 > 1.6; vitamin B_6_ (mg/day), T1 < 1.4, T2 1.4-2.1, T3 > 2.1; vitamin B_12_ (µg/day), T1 ≤3.8, T2 3.8-5.3, T3 > 5.3; Met (mg/day), for males, T1 < 1322.0, T2 1322.0-1986.0, T3 > 1986.0, and females, T1 < 1565.0, T2 1565.0-2623.0, T3 >2623.0; choline (mg/day), T1 < 114.0, T2 114.0-188.0, T3 >188.0; betaine (mg/day), T1 <117.0, T3 117.0-162.0, T3 > 162.0.

^e^Conditional exact logistic regression.
